# Regulation of CIRP by genetic factors of SP1 related to cold sensitivity

**DOI:** 10.3389/fimmu.2022.994699

**Published:** 2022-09-16

**Authors:** Soo Yeon Kim, Hyo-Jeong Ban, Siwoo Lee, Hee-Jeong Jin

**Affiliations:** Korean Medicine (KM) Data Division, Korea Institute of Oriental Medicine, Daejeon, South Korea

**Keywords:** Sp1, cold sensitivity, genetic factor, inflammatory response, CIRP, CIRPB

## Abstract

Cold-inducible RNA-binding-protein (CIRP) is a cold shock protein that plays a protective role in genotoxic stress response. CIRP modulates inflammation in human diseases, inhibits cell proliferation, and protects cells from genotoxic damage during cellular stress. The mild cold responsive element and specificity protein 1 (SP1) play a role in *Cirp* expression at low temperatures. Although previous studies have provided insights into the immune functions of SP1 or CIRP, the mechanisms by which CIRP and SP1 me diate inflammatory responses remain largely unknown. Therefore, in the current study, we examined whether *Cirp* expression is affected by genetic factors related to temperature sensitivity as well as under low temperature. We performed a genome-wide association study on cold sensitivity in 2,000 participants. Fifty-six genome-wide significant trait-locus pairs were identified (*p*<1×10^-5^, false discovery rate < 0.05). Among these variants, rs1117050 and rs11170510 had a strong linkage disequilibrium (r^2^ > 0.8) relationship and expression quantitative trait locus-associated signals with the nearest *Sp1* gene. We confirmed that the minor alleles of rs11170510 and rs58123204 were associated with increased *Sp1* expression. Additionally, *Sp1* overexpression led to CIRP translocation from the nucleus to the cytoplasm. CIRP protein levels increased in serum samples that had minor alleles of rs11170510 and rs58123204. Levels of various pro-inflammatory cytokines were also significantly increased in human peripheral blood mononuclear cells with minor alleles of rs11170510 and rs58123204. These results suggest that genetic factors related to cold sensitivity regulate *CIRP* expression and function and provide valuable insights into prediction of potential diseases through analysis of inherent genetic factors in humans.

## Introduction

Exposure to cold factors increases adrenocorticotropic hormone, adrenaline, noradrenaline, cortisol, and glucose levels in the blood (1–3). It also induces metabolic conditions, such as increased systemic energy expenditure and peripheral tissue energy use (4), which are more severe in men than in women (5). Upon exposure to cold conditions, protein synthesis is inhibited during cell preservation (6); however, various proteins such as cold-inducible RNA-binding-protein (CIRP, also known as CIRBP), show increased synthesis (6).

CIRP is one of the first identified cold shock proteins that is upregulated after exposure to mild cold-shock, UV radiation, hypoxia, and glucose deprivation in mammals (7–10). CIRP, which plays a protective role in the genotoxic stress response by stabilizing the transcripts of genes involved in cell survival, has been reported to be involved in various cellular processes such as proliferation, survival, circadian modulation, telomere maintenance, and tumorigenesis (11–14). Hypoxia, endotoxin, and alcohol stress can increase the expression of intracellular CIRP *via* pathways involving HIF-α, TLR4, NF-κB, Egr-1, AP-1, and STAT1, and cause extracellular CIRP (eCIRP) release in immune cells (15–17). Recent studies have indicated that eCIRP functions as a damage-associated molecule that binds directly to TLR4/MD2 and TREM-1 in various cells (15, 18, 19) and contributes to inflammatory diseases (15, 20–23).

Specificity protein 1 (SP1), a zinc finger transcriptional regulator, belongs to a large family of specificity protein/Kruppel-like factor transcription factors (24). It binds to GC-rich motifs of promoters in many genes and plays a critical role in transcriptional activation and repression related to various cellular processes (25, 26). SP1 also contributes to differentiation, growth, apoptosis, angiogenesis, and inflammation (27–30). Its overexpression increases *Cirp* expression in connection with SP1 in the 5′ flanking region of the *Cirp* gene (9). Although these reports have provided insights into the immune function of SP1 or CIRP, the mechanisms by which CIRP and SP1 mediate inflammatory responses remain largely unknown.

Therefore, in the present study, we investigated whether genetic factors regulate *Cirp* and *Sp1* expression and function.

## Material and methods

We performed a genome-wide association study (GWAS) on cold sensitivity involving 2,000 Koreans. Our study workflow is as shown in **Supplementary Figure 1**.

### Participants

The 2000 participants (611 males and 1389 females) were selected from the Korean Medicine Daejeon Citizen Cohort (KDCC) study (31), which is an ongoing cohort study assessing the associations between lifestyle factors and chronic diseases. The Institutional Review Board of the Korea Institute of Oriental Medicine and Dunsan Korean Medicine Hospital of Daejeon University reviewed and approved this study (IRB No. DJDSKH-17-BM-12) along with the regional ethics board. Written informed consent was obtained from all participants. Self-administered questionnaires on typical symptoms were used to evaluate cold and heat patterns experienced by the participants. The questionnaire consisted of 15 items (8 items on cold patterns and 7 items on heat patterns). All items were rated on a five-point Likert scale. Scores of the cold and heat patterns were the sums of responses to the relevant items, with a higher score indicating a higher level of the cold or heat pattern. A previous study suggested the cutoff point for the cold pattern to be 21.5 in both men and women and reported an agreement rate of 87.1% and a kappa value of 0.741 (31).

### Genetic association of cold sensitivity

Blood samples from patients were sent to the Seoul Clinical Laboratories & Seoul Medical Science Institute (SCL) for DNA extraction, after which the DNA quality and quantity were assessed based on the 260/280 nm and 260/230 nm ratios, respectively. The extracted DNA samples were then stored at −80°C until further processing. Further, all the DNA samples were amplified and randomly fragmented into 25–125 bp fragments, which were then purified, re-suspended, and hybridized to an Axiom array (TPMRA chip, Thermo Fisher, Seoul, Korea) that was customized based on the Asian Precision Medicine Research Array (Thermo fisher Scientific, Waltham, Massachusetts, USA). After hybridization, the bound target was washed under stringent conditions to remove non-specific background so as to minimize noise resulting from random ligation events. Finally, according to the manufacturer’s instructions, 820,000 SNPs were genotyped using the Theragen PMRA, which provides genome-wide coverage in five major populations.

To capture signals on unobserved and missing single nucleotide polymorphisms (SNPs) among genotyped data, we performed imputations on the ASN (n=286) reference panel of the 1000 Genome Project Phase I (32) integrated variant dataset using IMPUTE v2 software (33). We used on the NCBI Human Genome Build 37 (hg19) for the annotation and location of genes and SNPs. After imputation, 4,022,022 SNPs were analyzed following quality control (QC) procedures. Thereafter, variants with low call rates <95%, Hardy-Weinberg equilibrium failure <10E-6, and minor allele frequencies <5% were eliminated according to the QC procedures. Next, genome-wide association analyses were performed using PLINK 1.90 (https://www.cog-genomics.org/plink; accessed on October 17, 2021).

Linear regression analysis was performed to determine genetic associations with cold scores in additive models adjusted for sex and age and to determine genome-wide significance, SNPs were selected using a widely applied multiple-correction method, namely, the false discovery rate (FDR) method (Benjamini & Hochberg, 1995; FDR control) < 0.05.

To identify genes affected by the SNP allele type associated with cold sensitivity, we checked the eQTL values corresponding to the SNPs as well as gene expression data based on the GTEx whole blood database v7 (**Supplementary Table 3**). Next, after identifying genes located on the same chromosome (cis-eQTL results), we selected those with q < 0.05. Thus, it was possible to confirm the significance of *Sp1* gene expression regulation in SNPs in relation to cold sensitivity (34).

Additionally, we used the LocusZoom (35) web program to select 1 Mb of signals around the important SNPs. The population used for the LD regions as shown in the figure was a 2012 Asian population (ASN) panel mapped to hg19 genome builds. Further, to determine the LD of the Korean population, the LD block and r^2^ values were taken into consideration using the default options in PLINK (**Supplementary Table 4**).

### Cell culture

HEK 293 (CRL-1573™) cells were obtained from American Type Culture Collection (ATCC, Manassas, MDVA, USA) and cultured in Dulbecco’s modified Eagle’s medium (Gibco™, Waltham, MA, USA; 11965118) with 10% fetal bovine serum (Gibco™, 16000044) and 1% antibiotic-antimycotic (Gibco™, 15240062) in a humidified atmosphere in a 5% CO_2_ incubator at 37°C. THP-1 (TIB-202^™^) cells were obtained from ATCC and maintained in RPMI-1640 Medium (Gibco™, 21875034) containing 10% fetal bovine serum, 1% antibiotic-antimycotic and 0.05 mM 2-mercaptoethanol (Gibco™, 21985023). THP-1 cells were then stimulated with 50 nM phorbol myristate acetate (Sigma-Aldrich, St. Louis, MO, USA; P8139) for 24 h for induction of differentiation into macrophages.

### Preparation of plasmid construction

For the luciferase reporter assays, 100 bp of SNP analyzed (major or minor) sequences were synthesized by Bioneer Corporation (Daejeon, South Korea). The analyzed SNP sequences (major or minor) used were as follows: *Sp1-rs11170510-major*, 5′-CGGGCGTGGTGATGGGCACCTGTAGTCC CAGCTACTAGGGAGGCTGAGGCAGGAGAATGGCGTGAACCCAGGAGGCAGAGCTTGCAGTGAGCGGAGATGGC-3′; *Sp1-rs11170510-minor*, 5′-CGGGCGTGGTGATGGGCACCTGTAG TCCCAGCTACTAGGGAGGCTGAGGCGGGAGAATGGCGTGAACCCAGGAGGCAGAGCTTGCAGTGAGCGGAGATGGC-3′; *Sp1-rs58123204-major, 5*′-CGGTGGCTCACGCTTGTAATCCCAGCACTTTGGGAGGCCGAAGCAGGTGGATCACGAGGTCAGGAGATCCAGACCATCCTGGCTAACACGTTGAAACCCGG-3′; *Sp1-rs58123204-minor*, 5′-CGGTGGCTCACGCTTGTAATCCCA GCACTTTGGGAGGCCGAAGCAGGTGGGTCACGAGGTCAGGAGATCCAGACCATCCTGGCTAACACGTTGAAACCCGG-3′. The synthesized sequences were cloned into the pGL4.23 [luc2/minP] vector (Promega, Madison, WI, USA; E841A) between the MluI and XhoI restriction sites to generate expression vectors. To produce the *Cirp* promoter plasmid, a 3000 bp of 5′-flanking region sequence of the *Cirp* gene was synthesized by Bioneer Corporation, and the *Cirp* promoter sequence was cloned into the pGL4.23 [luc2/minP] vector. The *Sp1* plasmid (24543) was purchased from Addgene (Watertown, MA, USA). The analysis of DNA-binding sites of transcription factors in the promoter region of *Cirp* was identified using a bioinformatic tool (http://www.cbrc.jp/research/db/TFSEARCH.html).

### Luciferase reporter assays

HEK293 cells were transfected using a luciferase gene under the control of the pGL4.23 [luc2/minP] promoter, and for the luciferase reporter gene assays, pGL4.23-major allele sequences, pGL4.23-minor allele sequences, pGL4.23-*Cirp*, and the *Sp1* plasmid were used. HEK293 cells (5x10^5^/well) were plated in 12-well culture plates. After 24 h, the cells were co-transfected with 10 ng of Renilla luciferase (pRL-TK) plasmid and pGL4.23-major allele sequences or pGL4.23-minor allele sequence plasmid (1 μg) using Lipofectamine 2000 (Invitrogen, Waltham, MA, USA; 11668027) according to the manufacturer’s protocol. Further, HEK293 cells (5x10^5^/well, were plated in 12-well culture plates, and after culturing for 24 h, the cells were co-transfected with the pGL4.23-*Cirp* plasmid (1 μg) in the presence or absence of the *Sp1* plasmid (0.1, 0.5, and 1 μg) and 10 ng of Renilla luciferase (pRL-TK) plasmid using Lipofectamine 2000 (Invitrogen, Waltham, MA, USA; 11668027) according to the manufacturer’s protocol. Twenty-four hours after transfection, the cells were lysed with reporter lysis buffer (Promega, E1941), and luciferase activity of the lysates was measured using a GloMax20/20 Luminometer (Promega, E5311) on the Dual-Luciferase^®^ Reporter Assay System (Promega, E1910) according to the manufacturer’s protocols. Luciferase activity was normalized to that of Renilla luciferase activity.

### PBMC RNA extraction and quantitative PCR

Total RNA from peripheral blood mononuclear cells (PBMCs) (1x10^6^) was purified using RNeasy Plus Micro Kit (Qiagen, GmBH, Hilden Germany; 74034) according to the manufacturer’s protocol. cDNA was synthesized with 1 µg of RNA using SuperScript™ First-Strand Synthesis System (Invitrogen, 11904018) according to the manufacturer’ instrument. A total of 5 ng cDNA from each sample was amplified using a Power SYBR™ Green PCR Master Mix (Applied Biosystems™, Waltham, MA, USA; 4367659), primers, and a PCR cycler Rotor-Gene Q 2plex system (Qiagen, 9001620). The PCR conditions were as follows: 35 cycles at 95°C for 10 sec, 55°C for 30 sec, and 72°C for 1 min. qPCR data were analyzed using the 2^ΔΔCt^ method with *Gapdh* as a housekeeping gene; data are expressed as relative fold changes. The following primers were used: *Sp1*, 5′ caagtaatcccacagttcca 3′ (forward) and 5′ ctgagtctgtcctgagagta 3′ (reverse); *Cirp*, 5′ cagatgaaggcaaactttttgt 3′ (forward) and 5′ tgttctcaaaggtgacaaacc 3′ (reverse); *Tnf*-*α*, 5′ cagggacctctctctaatca 3′ (forward) and 5′ tgggagtagatgaggtacag 3′ (reverse); *Il6* 5′ tttaaatctccaggcttccc 3′ (forward) and 5′ agctcatctccacagtatct 3′ (reverse); *Il8* primers 5′ acctttccaccccaaatttat 3′ (forward) and 5′ aaaacttctccacaaccctc 3′ (reverse); *Il-1β*, primers 5′ ccctaaacagatgaagtgct 3′ (forward) and 5′ gttatcccatgtgtcgaaga 3′ (reverse); *Gapdh* primers 5′ aacctgccaaatatgatgaca 3′ (forward) and 5′ ataccaggaaatgagcttgac 3′ (reverse).

### ELISA

Human serum samples were collected and stored at −80°C. Thereafter, CIRP protein levels in serum samples were detected using an ELISA kit (Cusabio, Houston, TX, USA; CSB-EL005440HU) according to the manufacturer’s protocol.

### Immunofluorescence analysis

THP-1 cells (5x10^5^/well) were plated in 12-well culture plates, and after 24 h, the cells were transfected with the pGL4.23-*Cirp* plasmid (1 μg) in the presence or absence of the *Sp1* plasmid (0.1, 0.5, and 1 μg) and 10 ng of Renilla luciferase (pRL-TK) plasmid using Lipofectamine^®^ LTX Reagent (Invitrogen, TIB-202) according to the manufacturer’s protocol. Next, after 24 h, the cells were washed with phosphate-buffered saline (PBS, Gibco™, 10010023), fixed with 4% paraformaldehyde (Santa Cruz Biotechnology, Dallas, TX, USA; sc-281692) for 15 min, and permeabilized with 0.25% Triton X-100 (Sigma-Aldrich, T8787) for 10 min. Cells were then rinsed with PBS, and stained with the primary antibody anti-CIRP (Novus, Littleton, CO, USA; NBP2-15905) for 16 h at 4 °C. After 16 h, primary antibodies were removed and the cells were washed with PBS, followed by staining with anti-Rabbit IgG (H+L) Cross-Adsorbed Secondary Antibody, Alexa Fluor™ 488 (Molecular Probes, Eugene, OR, USA; A-11008) for 2 h at room temperature. Next, the cells were stained with DAPI (Sigma-Aldrich, D8417) for 1 min. Fluorescence images were acquired with a Zeiss confocal microscope (LSM 710, Zeiss, Oberkochen, Germany). The images captured were then analyzed using ZEN 2009 software version 5.5 SP1 (Zeiss). Notably, all the experiments were performed in triplicate on coverslips and the results obtained were presented as the mean ± standard deviation.

### Statistical analysis

Experiment data are expressed as mean ± standard deviation. All experiments were performed at least three times. Mann–Whitney U test was performed to analyze statistical significance between paired groups using SPSS for Windows (ver. 21.0; IBM, Armonk, NY, USA). Differences were considered statistically significant at p<0.05.

## Results

### Genome-wide association study

**Figure 1:**
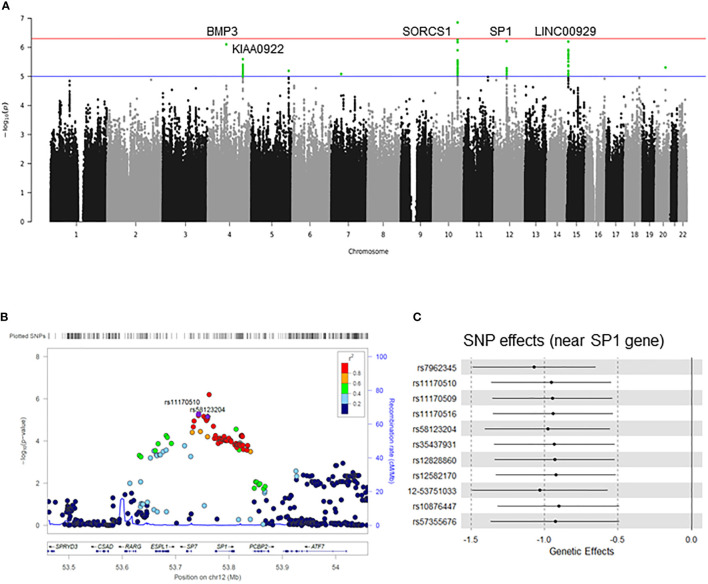
Genome-wide association study (GWAS) on cold sensitivity. **(A)** Manhattan plot and quantile-quantile plot for the GWAS of cold sensitivity. The blue horizontal line indicates P values < 10-5, while the red horizontal line indicates P values < 10-7. The loci with well-characterized genes are indicated close to the association peaks. **(B)** LocusZoom plot of rs11170510 and rs58123204 in the SP1 gene. In these plots, each point represents a –log(P) value in the linear regression analysis as a function of genomic position (NCBI Human Genome Build 37). The color codes of all the other SNPs represent LD, with the lead SNP (estimated based on hg19/1000 Genomes Mar 2021 ASN): red, r2 ≥ 0.5; gold, 0.6 ≤ r2 < 0.8; green, 0.4 ≤ r2 < 0.6; cyan, 0.2 ≤ r2 < 0.4; blue, r2 < 0.2; gray, r2 unknown. **(C)** Forest plot showing the genetic effect size between single nucleotide polymorphisms (SNPs) and cold sensitivity.

We performed a GWAS on cold sensitivity in 2,000 participants (**Supplementary Table 1**) of the KDCC study (**Supplementary Figure 1**) (31). Of the 56 variants that were genome-wide significant (*p*<1×10^-5^, false discovery rate < 0.05) in the discovery GWAS (**Figure 1A**), seven variants (**Table 1**) showed evidence of the presence of eQTL-associated signals with the *Sp1* gene based on the cis-eQTL data from the GTEx database (v7) (36). To identify potentially causative SNPs, we used LocusZoom to identify the most strongly associated SNPs (rs11170510 and rs58123204) and linkage disequilibrium patterns within 1 Mb around the lead SNP (**Figure 1B**). rs11170510 showed a strong linkage disequilibrium (r^2^>0.8) relationship with the nearest *Sp1* gene. Therefore, we selected a candidate locus (chr12: 53,684,619–53,813,402, near the *Sp1* gene) to measure the genetic influence of cold sensitivity and investigated its functional role (**Figure 1C** and **Table 1**).

**Table 1:** Significant genetic loci associated with cold sensitivity.

SNP	CR	BP	BETA	SE	L95	U95	P	FDR	MAF	Alleles	Nearest Gene	eQTL p value (Blood)
rs11170509	12	53743050	-0.9458	0.2087	-1.355	-0.5368	6.17E-06	0.02598	0.24	C>T	SP1	2.38E-08
rs11170510	12	53743734	-0.9534	0.2087	-1.363	-0.5442	5.25E-06	0.02598	0.24	A>G	SP1	3.98E-08
rs11170516	12	53752692	-0.9418	0.2087	-1.351	-0.5327	6.79E-06	0.02598	0.24	G>A	SP1	1.62E-08
rs35437931	12	53756354	-0.932	0.2086	-1.341	-0.5232	8.32E-06	0.02598	0.24	T>C	SP1	1.64E-08
rs12828860	12	53759803	-0.9302	0.2085	-1.339	-0.5216	8.56E-06	0.02598	0.24	G>C	SP1	1.64E-08
rs58123204	12	53760162	-0.9785	0.2171	-1.404	-0.5531	6.95E-06	0.02598	0.22	A>G	SP1	3.57E-08
rs7962345	12	53762887	-1.069	0.2136	-1.488	-0.6503	6.13E-07	0.02598	0.23	C>T	SP1	4.41E-07

BP, base-pair position; CR, Chromosome; eQTL, expression quantitative trait locus; FDR, false discovery rate; GWAS, genome-wide association study; MAF, minor allele frequency; SE, Standard error of Beta; SNP, single nucleotide polymorphism. L95, lower 95 confidence interval; U95, upper 95 confidence interval; P, p value in linear regression for cold sensitivity.

### Functional SNPs rs11170510 and rs58123204 regulate *Sp1* expression

To determine the functional role of the seven variants (**Table 1**) that provided evidence of eQTL-associated signals with the *Sp1* gene, we performed an allele-specific cell-based luciferase reporter assay (**Supplementary Figure 3**). Specifically, we selected the SNPs, rs11170510 and rs58123204, which showed the highest promoter activity among the seven variants.

**Figure 2:**
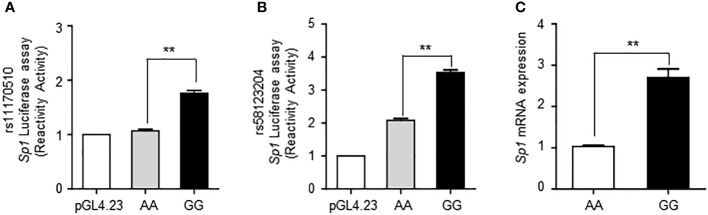
Functional effects of SNPs rs11170510 and rs58123204 on Sp1 expression. **(A)** HEK293 cells were transfected with the mock vector (pGL4.23 vector), major A allele of the rs11170510 or minor G allele of the rs11170510-luciferase reporter constructs. Cells were subjected to a luciferase assay. **(B)** HEK293 cells were transfected with the mock vector (pGL4.23 vector) or major A allele of the rs58123204 or minor G allele of the rs58123204-luciferase reporter constructs. Cells were subjected to a luciferase assay. **(C)** Quantitative PCR analysis of Sp1 mRNA expression in peripheral blood mononuclear cells (PBMCs) with major A allele or minor G allele of rs11170510 and rs58123204 (n = 10, major homo AA; n = 10, minor homo GG). Experiments were repeated at least three times. Graphs are indicative of the mean ± standard deviation (SD). **p < 0.01 (two-tailed unpaired Mann–Whitney).

To confirm the more functional roles of the SNPs rs11170510 and rs58123204, we conducted an allele-specific cell-based luciferase reporter gene assays to determine if SNP-containing sequences played the role of regulators. We replaced the pGL4.23 minimal vector with either the major or minor alleles of rs11170510 or rs58123204, respectively. Luciferase assays showed that the minor G allele had a significantly higher promoter activity than the major A allele at rs11170510 in HEK293 cells (**Figure 2A**). Luciferase assays showed that the minor G allele had a significantly higher promoter activity than the major A allele at rs58123204 in HEK293 cells (**Figure 2B**). The minor G allele of rs11170510 or rs58123204 affected *Sp1* promoter activity (**Figures 2A, B**). Thus, we determined whether rs11170510 and rs58123204 cause *Sp1* mRNA expression in allele-dependent manner in human PBMCs of KDCC (*n* = 10, major homo AA; *n* = 10, minor homo GG). As shown in **Figure 2C**, the minor G allele of rs11170510 and rs58123204 led to an increase in *Sp1* mRNA levels, when compared with PBMCs that had major A allele of rs11170510 and rs58123204. Therefore, the minor alleles of rs11170510 and rs58123204, which are genetic factors, promote transcriptional activation of *Sp1*.

### SP1 upregulates the transcriptional activation of *Cirp* in minor allele of rs11170510 and rs58123204

**Figure 3:**
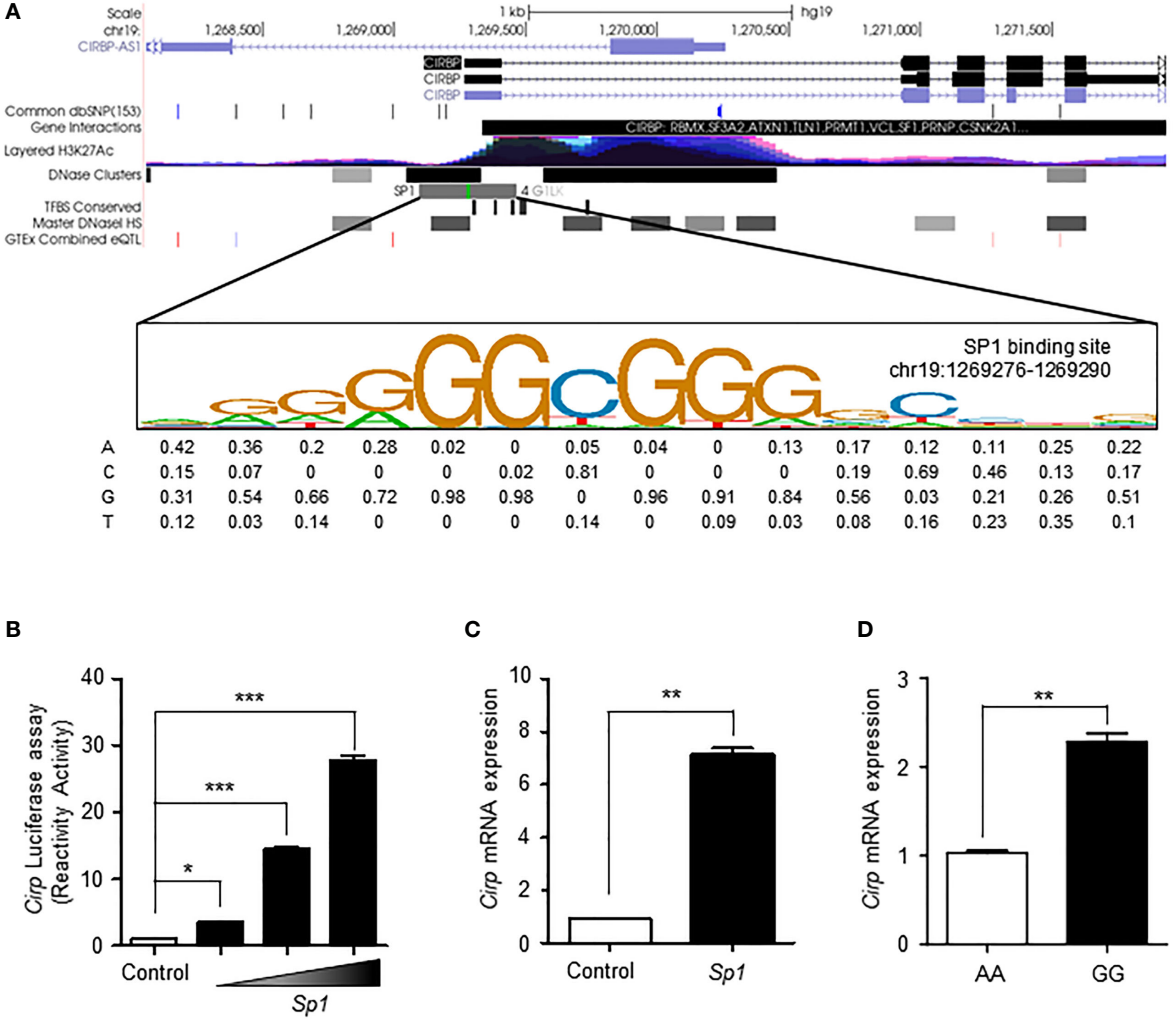
SP1 regulates the transcriptional activation of CIRP by rs11170510 and rs58123204. **(A)** A strong transcription factor cluster appeared in the Cirp transcriptional promoter region. The SP1 transcription factor binding site on the Cirp promoter was located in the region chr19:1269276-1269290. **(B)** HEK293 cells were transfected with a Cirp luciferase reporter construct in the presence or absence of plasmid Sp1 (0.1, 0.5, and 1μg). Cells were subjected to luciferase assays. **(C)** HEK293 cells were transfected plasmid Sp1 (1 µg) and analyzed using quantitative PCR for Cirp mRNA expression. **(D)** PBMCs with major A allele or minor G allele of rs11170510 and rs58123204 were subjected to quantitative PCR for Cirp mRNA using its primer (n = 10, major homo AA; n = 10, minor homo GG). Experiments were repeated at least three times. Graphs are indicative of the mean ± SD. *p < 0.05, **p < 0.01 and ***p < 0.001 (two-tailed unpaired Mann–Whitney).

A previous study reported an enhancer in the 5′ flanking region of the *Cirp* gene, which had a mild cold responsive element that can be recruited by SP1 to activate gene transcription (9). We searched the ENCODE database to determine the degree of transcriptional activity of the *Cirp* transcriptional promoter region. According to the ENCODE ChIP-sequencing data analysis (33), the peak cluster indicating the occupancy of transcription factors showed strong intensity, and DNaseI hypersensitivity clusters and H3K27Ac epigenetic mark were present. The SP1 transcription factor binding site closest to the *Cirp* promoter remained in the high motif consensus in chr19:1269276-1269290 (**Figure 3A**).

Next, we assessed the mechanistic role of SP1 in the transcriptional regulation of *Cirp* by performing luciferase assays with the promoter construct of *Cirp* (**Figure 3B**). The consensus SP1-binding motifs in the promoter regions of *Cirp* are shown in **Figure 3A**. The data showed that the overexpression of SP1 significantly upregulated the activity of promoter constructs of *Cirp* in HEK293 cells (**Figure 3B**). We conducted RT-qPCR to assess the mRNA expression of *Cirp* by SP1 in HEK293 cells. The overexpression of SP1 significantly amplified *Cirp* mRNA levels in HEK293 cells (**Figure 3C**). We confirmed that SP1 upregulates the transcriptional activation of *Cirp* by recruiting SP1 in the 5′ flanking region of the *Cirp* gene. Thus, we questioned whether increased *Sp1* expression by the minor G allele of rs11170510 and rs58123204 affected *Cirp* mRNA expression in human PBMCs. We examined the levels of *Cirp* mRNA using the major or minor alleles of rs11170510 and rs58123204 in human PBMCs of KDCC (*n* = 10, major homo AA; *n* = 10, minor homo GG) and found that the mRNA levels of *Cirp* were increased in the PBMCs that contained the minor alleles of rs11170510 and rs58123204 (*n* = 10, major homo AA), when compared with in the PBMCs that had their major alleles *(n* = 10, minor homo GG) (**Figure 3D**). Further, as shown in **Figure 3D**, increased *Sp1* mRNA levels owing to the minor G allele of rs11170510 and rs58123204 contributed to increase in *Cirp* mRNA levels, when compared with the PBMCs that had major A alleles of rs11170510 and rs58123204.

These results suggest that increases in *Cirp* mRNA levels are regulated by SP1, which is affected by the minor alleles of rs11170510 and rs58123204 in the PBMCs.

### CIRP causes an inflammatory response by the genetic variants

**Figure 4:**
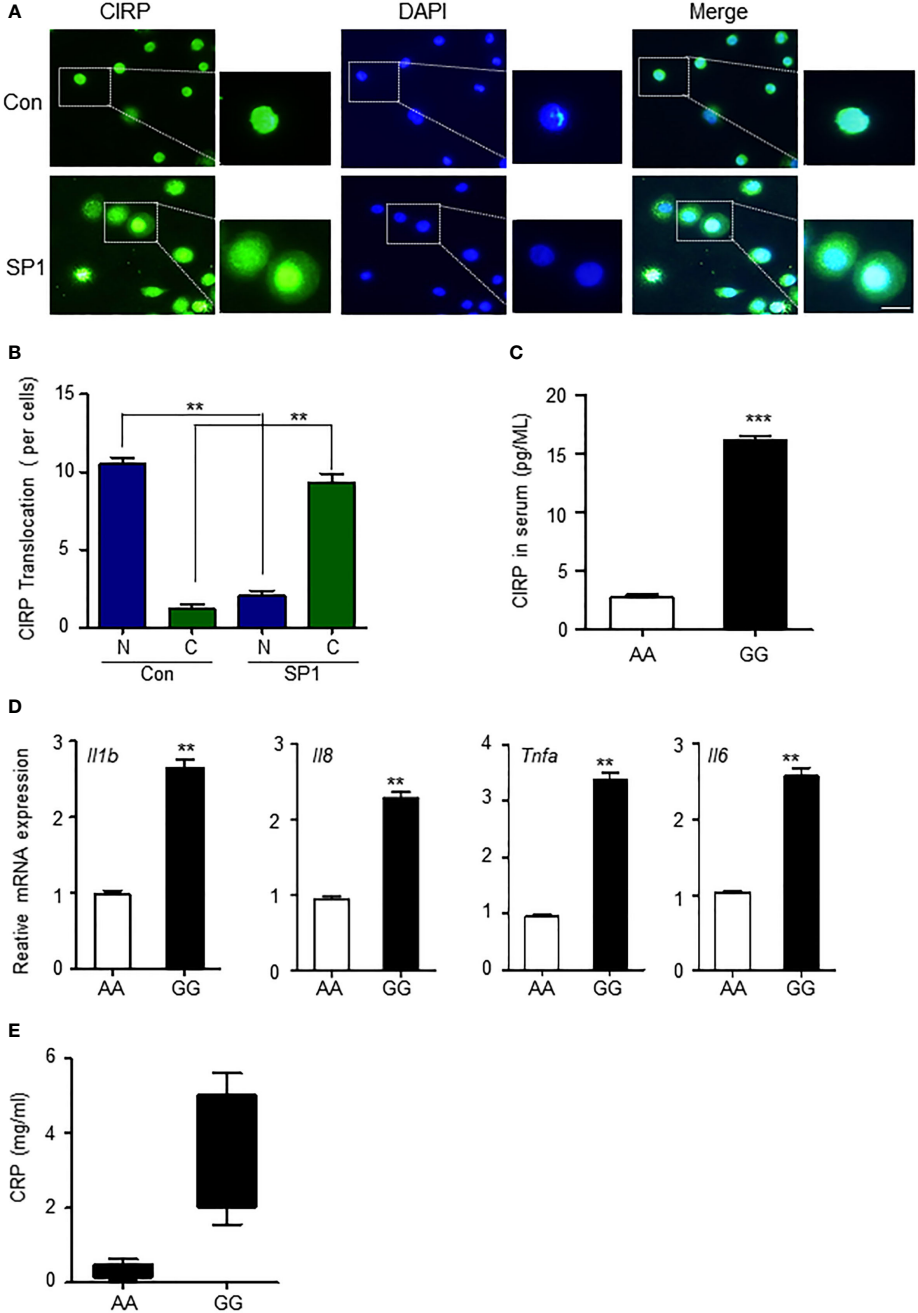
CIRP leads to increased inflammatory process in the PBMCs by rs11170510 and rs58123204. **(A)** THP-1 cells were transfected with the Sp1 plasmid for 24 (h) Immunofluorescence microscopy analysis of CIRP. Merged signals of CIRP (Alexa Fluor 488-conjugated goat anti-rabbit IgG, green) and DAPI (nuclei, blue). scale bar: 10 um. **(B)** Quantification of translocation in then nucleus to cytosol foci per cell. **(C)** CIRP levels were measured in serum with major allele or minor allele of the rs11170510 and rs58123204 (n = 10, major homo AA; n = 10, minor homo GG) using CIRP ELISA kit. **(D)** PBMCs were analyzed using the quantitative PCR of Tnf-α, Il6, Il-1β, and Il8 mRNA levels. **(E)** C-reactive protein levels were measured in serum containing major A allele or minor G allele of rs11170510 and rs58123204 (n = 10, major homo AA; n = 10, minor homo GG). Experiments were repeated at least three times. Graphs are indicative of the mean ± SD. **p < 0.01 and ***p < 0.001 (two-tailed unpaired Mann–Whitney).

CIRPs mainly exist in the nucleus (7), but their cellular location is in a state of flux. It has been reported that during hypoxia, CIRP can be induced and translocated from the nucleus to the cytoplasm and then gradually released into the extracellular space by lysosomes in macrophages (15). Extracellular CIRP further plays an important role in the inflammatory process (21). We wondered whether SP1 affected the translocation of CIRP from the nucleus to the cytoplasm. First, we confirmed whether increased *Sp1* expression by minor alleles of rs11170510 and rs58123204 led to the translocation of CIRP from the nucleus to cytosol to examine the transfection of the plasmid *Sp1* into THP-1 cells. CIRP migrated from the nucleus to the cytosol of SP1-overexpressed THP-1 cells (**Figure 4A**). The proportion of CIRP translocated in the cytoplasm was markedly enhanced compared to that in the nucleus (**Figures 4A, B**). We therefore suggest that increased *Sp1* expression by the minor alleles of rs11170510 and rs58123204 may induce CIRP translocation from the nucleus to the cytoplasm. Cytoplasmic CIRP can further be released in serum. Thus, we examined the possibility of the release of cytoplasmic CIRP into serum that contained major or minor alleles of rs11170510 and rs58123204 (*n* = 10, major homo AA; *n* = 10, minor homo GG). CIRP was detected in serum using a CIRP ELISA kit. The levels of the CIRP protein increased in serum that contained the minor alleles of rs11170510 and rs58123204, when compared with serum that had their major alleles (**Figure 4C**).

Next, we investigated whether increased CIRP in serum that had the minor alleles of rs11170510 and rs58123204 affected the inflammatory response. Pro-inflammatory cytokine generation in the PBMCs with major or minor alleles of rs11170510 and rs58123204 was assessed. We thus investigated pro-inflammatory cytokine generation for major or minor allele of rs11170510 and rs58123204 in the PBMCs. Tumor necrosis factor (*Tnf*)-*α*, interleukin (*Il)-6*, *Il-1β*, and *Il8* mRNA levels were significantly increased in the PBMCs with minor alleles of rs11170510 and rs58123204, compared with those in the PBMCs with their major alleles (**Figure 4D**). We also found that the levels of C-reactive protein, a sensitive marker of inflammation, were increased in the serum that had the minor alleles of rs11170510 and rs58123204 when compared to those in the serum that contained their major alleles (**Figure 4E**).

These results suggest that CIRP regulates inflammatory responses *via* the minor alleles of rs11170510 and rs58123204.

## Discussion

Changes in external temperature are perhaps the most common form of environmental stress that all organisms undergo regularly. Common responses to stress include the inhibition of transcription, translation, and metabolic processes. Cold shock proteins have been identified in *E. coli*, the best-characterized and reported model organism to date (37). CIRP, serving as a representative gene expressed under environmentally induced cold stress, plays various cellular processes such as proliferation, survival, circadian modulation, telomere maintenance, and tumorigenesis. In this work, we investigated whether genetic factors regulate CIRP, independent of the external environment.

To confirm the regulation of temperature-related gene expression by genetic factors in addition to environmental factors in humans, we conducted a survey on cold sensitivity in the population and generated genomic data. The central goal of human genetics is to understand how genetic variations lead to phenotypic differences and complex diseases (38). Numerous recent GWASs have reported genetic variations associated with various human traits and diseases (39). These genetic variations exist in regions that affect gene expression, such as promoters and enhancers, and play a central role in complex traits (40). The eQTL approach has enabled interpretable molecular connections between genetic variations and phenotypes, enabling the study of non-model organisms, such as humans (41). We identified five statistically significant gene regions through a GWAS on cold sensitivity. A strong eQTL relationship was confirmed in all the tissues in the *Sp1* region among the five significant gene regions. Therefore, it was confirmed that genetic factors related to cold sensitivity also affect protein expression.

SP1 is a well-known transcription factor involved in various biological processes. Recently, several studies have reported a role for SP1 in the immune system (42–46). SP1 is also known to be involved in inflammation, which is an element of the immune system (29, 47–51). A previous study revealed that the upregulation of SP1 increased damage to dopaminergic neurons and induced oxidative stress and inflammation in a model of Parkinson’s disease (47). The mineralocorticoid receptor/ERK-SP1 pathways are involved in aldosterone-induced inflammatory and fibrotic responses in vascular smooth muscle cells (50). SP1-TGF-β/Smad3-NFκB-dependent mechanisms have been found to lead to ANG II-mediated renal inflammation and fibrosis in mice (51). These findings indicate that SP1 is a crucial regulator of inflammation.

Here, we found that *Sp1* exhibits genetic variation associated with cold sensitivity, which causes inflammatory responses by regulating CIRPs. Our results showed that SP1 is regulated by the genetic factors rs11170510 and rs58123204, which can upregulate the mRNA expression of *Sp1* in the PBMCs. It is known that various SNPs affect gene expression and are thus involved in a variety of biological processes. Plourde *et al.* (2018) reported that SNPs in *Lrrfip1* are associated with adiposity and inflammation (52). The ADA genetic variant was further found to be associated with central inflammation and clinical presentations in multiple sclerosis (53). Our findings suggest that the minor alleles of rs11170510 and rs58123204 act as key of genetic factors related to cold sensitivity and promote the transcriptional activation of *Sp1*, which regulates the expression and function of *Cirp*. However, given that PBMCs contain various cells other than the monocytes used to verify translocation of CIRP by overexpressed *Sp1* owing to genetic factors, rs11170510 and rs58123204, in this study, we could not interpret all the results related to the PBMCs. Therefore, it is necessary to verify *Cirp* expression and regulation owing to *Sp1*-related genetic factors for each cell type using single-cell ATAC sequencing.

We demonstrated that CIRP is increased at the mRNA level and is translocated from the nucleus to the cytoplasm by overexpressed *Sp1* in cells. SP1 has been strongly implicated in hypoxic gene transcription (54). We therefore suggest that SP1 can induce hypoxia, which may mediate the migration of CIRP from the nucleus to the cytoplasm in cells. Our results also showed that eCIRP levels were higher in the serum with minor homo than in the serum with major homo of rs11170510 and rs58123204. Thus, we suggested that increased serum eCIRP levels in the serum may be associated with elevated *Sp1* expression related to the minor homo (rs11170510 and rs58123204). Qiang *et al.* (2018) reported that CIRP can be released *via* the lysosomal secretion process (15). Based on these studies and our findings, the mechanisms underlying CIRP release need to be further studied in relation to overexpressed *Sp1*. A recent study revealed that eCIRP binds to TLR4 and induces NF-κB activation, leading to the synthesis of pro-inflammatory cytokines and chemokines in immune cells (15). Our findings also show that the minor alleles of rs11170510 and rs58123204 increase pro-inflammatory cytokine levels, such as those of *Tnf-α*, *Il6*, *Il-1β* and *Il8*.

Previous studies have confirmed that CIRP affects the progression of various diseases. It has also been observed that eCIRP can act as a damage-associated molecular pattern (DAMP) that promotes inflammation and injury (55–58). Additionally, CIRP regulates cardiac electrophysiological properties, atrial fibrillation susceptibility, and sinoatrial node function in response to stress (59), and Jang rom also showed that CIRP may be a marker of good prognosis in colon cancer (60). Thus, the usefulness of CIRP in various diseases has been confirmed. Our result in this study showed that in future, it would be necessary to consider congenital genetic factors in relation to various diseases in CIRP-based studies. Further, this is also of great significance given that it widens the scope for future studies in which in addition to environmental factors, other factors are also considered.

Taken together, genetic factors, including SNPs, associated with cold sensitivity regulate SP1, which contributes to the function of CIRP in inflammation. Approaches based on targeting SP1-CIRP through GWASs associated with cold sensitivity may provide a promising new approach for assessing individual traits related to genetic factors. However, we could not identify genetic factors for other ethnicities, and even though for other ethnic groups, genetic studies involving individuals with fever or those exposed to extreme cold are being conducted, general temperature sensitivity has not been investigated. Given that this study has shown that there is a change in the expression of the *Cirp* gene due to a genetic difference even in the absence of external environmental difference, our results are expected to serve as a guide for genetic research on temperature sensitivity.

## Data availability statement

The original contributions presented in the study are included in the article/**Supplementary Material**. Further inquiries can be directed to the corresponding author.

## Ethics statement

The studies involving human participants were reviewed and approved by The Institutional Review Board of the Korea Institute of Oriental Medicine and Dunsan Korean Medicine Hospital of Daejeon University (IRB No. DJDSKH-17-BM-12) along with the regional ethics board. The patients/participants provided their written informed consent to participate in this study.

## Author contributions

Conceptualization: H-JJ, SY, and H-JB. Data curation: H-JB. Experiments and data analysis: SY. GWAS and functional study: H-JB. Resources: SW. Writing - original draft: H-JJ, SY, and H-JB. Writing - review and editing: H-JJ, SY, and H-JB. Project administration: H-JJ. All authors contributed to the article and approved the submitted version.

## Funding

This study was supported by the “Development of Korean Medicine Original Technology for Preventive Treatment based on Integrative Big Data” grant from the Korea Institute of Oriental Medicine [grant number KSN2023120].

## Acknowledgments

We thank the clinical team for of the Korean Medicine Daejeon Citizen Cohort Study (KDCC).

## Conflict of interest

The authors declare that the research was conducted in the absence of any commercial or financial relationships that could be construed as a potential conflict of interest.

## Publisher’s note

All claims expressed in this article are solely those of the authors and do not necessarily represent those of their affiliated organizations, or those of the publisher, the editors and the reviewers. Any product that may be evaluated in this article, or claim that may be made by its manufacturer, is not guaranteed or endorsed by the publisher.

## Supplementary material

Supplementary Figure 1 Workflow of the study design. 2,000 Koreans were recruited and DNA was collected along with a questionnaire about cold sensitivity. The DNA was genotyped using a TPMRA chip and linear regression analysis was performed between genotype and cold sensitivity score. The changes in Sp1 and Cirp gene expression according to the cold sensitivity-related genetic loci were confirmed through an experimental verification experiment for SNPs that were statistically significant (FDR<0.05) from the results.

Supplementary Figure 2 QQ plot of p-values based on GWAS for the cold sensitivity. The results did not show any inflation of the test statistics (lambda = 1.024)

Supplementary Figure 3 Functional effects of SNPs rs11170509, rs11170516, rs35437931, rs7962345, rs12828860, rs11170510, and rs58123204. HEK293 cells were transfected with the mock vector (pGL4.23 vector), major A allele of the rs11170509, rs11170516, rs35437931, rs7962345, rs12828860, rs11170510, and rs58123204 or minor G allele of the rs11170509, rs11170516, rs35437931, rs7962345, rs12828860, rs11170510, and rs58123204-luciferase reporter constructs. Cells were subjected to a luciferase assay. Experiments were repeated at least three times. Graphs are indicative of the mean ± standard deviation (SD). **p < 0.01 (two-tailed unpaired Mann–Whitney).

## Abbreviations

CIRP, Cold-inducible RNA-binding protein; SP1, specificity protein 1; eCIRP, extracellular CIRP; KDCC, Korean Medicine Daejeon Citizen Cohort; SNPs, single nucleotide polymorphisms; GWAS, genome-wide association study; PBMCs, peripheral blood mononuclear cells; PBS, phosphate-buffered saline; eQTLexpression quantitative trait locus.

## References

[1] Gein SV Sharav’eva IL. Immunomodulating effects of cold stress. Biol Bull Rev (2018) 8(6):482–8. doi: 10.1134/s207908641806004x

[2] Cong P Liu Y Liu N Zhang Y Tong C Shi L. Cold exposure induced oxidative stress and apoptosis in the myocardium by inhibiting the Nrf2-Keap1 signaling pathway. BMC Cardiovasc Disord (2018) 18(1):36. doi: 10.1186/s12872-018-0748-x 29448942 PMC5815212

[3] Blondin DP Labbe SM Phoenix S Guerin B Turcotte EE Richard D. Contributions of white and brown adipose tissues and skeletal muscles to acute cold-induced metabolic responses in healthy men. J Physiol (2015) 593(3):701–14. doi: 10.1113/jphysiol.2014.283598 PMC4324714 25384777

[4] Hao Q Yadav R Basse AL Petersen S Sonne SB Rasmussen S. Transcriptome profiling of brown adipose tissue during cold exposure reveals extensive regulation of glucose metabolism. Am J Physiol Endocrinol Metab (2015) 308(5):E380–92. doi: 10.1152/ajpendo.00277.2014 25516548

[5] Solianik R Skurvydas A Vitkauskiene A Brazaitis M. Gender-specific cold responses induce a similar body-cooling rate but different neuroendocrine and immune responses. Cryobiology (2014) 69(1):26–33. doi: 10.1016/j.cryobiol.2014.04.015 24809633

[6] Zhu X Buhrer C Wellmann S. Cold-inducible proteins cirp and Rbm3, a unique couple with activities far beyond the cold. Cell Mol Life Sci (2016) 73(20):3839–59. doi: 10.1007/s00018-016-2253-7 PMC5021741 27147467

[7] Nishiyama H Itoh K Kaneko Y Kishishita M Yoshida O Fujita J. A glycine-rich rna-binding protein mediating cold-inducible suppression of mammalian cell growth. J Cell Biol (1997) 137(4):899–908. doi: 10.1083/jcb.137.4.899 9151692 PMC2139845

[8] Fujita J. Cold shock response in mammalian cells. J Mol Microbiol Biotechnol (1999) 1(2):243–55. 10943555

[9] Sumitomo Y Higashitsuji H Higashitsuji H Liu Y Fujita T Sakurai T. Identification of a novel enhancer that binds Sp1 and contributes to induction of cold-inducible rna-binding protein (Cirp) expression in mammalian cells. BMC Biotechnol (2012) 12:72. doi: 10.1186/1472-6750-12-72 23046908 PMC3534229

[10] Liu Y Liu P Hu Y Cao Y Lu J Yang Y. Cold-induced rna-binding protein promotes glucose metabolism and reduces apoptosis by increasing akt phosphorylation in mouse skeletal muscle under acute cold exposure. Front Mol Biosci (2021) 8:685993. doi: 10.3389/fmolb.2021.685993 34395524 PMC8358400

[11] Yang C Carrier F. The uv-inducible rna-binding protein A18 (A18 hnrnp) plays a protective role in the genotoxic stress response. J Biol Chem (2001) 276(50):47277–84. doi: 10.1074/jbc.M105396200 11574538

[12] Park BM Lee JH Kim SJ. Expression of cold-inducible rna-binding protein in normal skin, actinic keratosis and squamous cell carcinoma. Ann Dermatol (2014) 26(2):256–8. doi: 10.5021/ad.2014.26.2.256 PMC4037683 24882985

[13] Sakurai T Itoh K Higashitsuji H Nonoguchi K Liu Y Watanabe H. Cirp protects against tumor necrosis factor-Alpha-Induced apoptosis *via* activation of extracellular signal-regulated kinase. Biochim Biophys Acta (2006) 1763(3):290–5. doi: 10.1016/j.bbamcr.2006.02.007 16569452

[14] Nishiyama H Xue JH Sato T Fukuyama H Mizuno N Houtani T. Diurnal change of the cold-inducible rna-binding protein (Cirp) expression in mouse brain. Biochem Biophys Res Commun (1998) 245(2):534–8. doi: 10.1006/bbrc.1998.8482 9571190

[15] Qiang X Yang WL Wu R Zhou M Jacob A Dong W. Cold-inducible rna-binding protein (Cirp) triggers inflammatory responses in hemorrhagic shock and sepsis. Nat Med (2013) 19(11):1489–95. doi: 10.1038/nm.3368 PMC3826915 24097189

[16] Mandrekar P Szabo G. Signalling pathways in alcohol-induced liver inflammation. J Hepatol (2009) 50(6):1258–66. doi: 10.1016/j.jhep.2009.03.007 PMC3342816 19398236

[17] Rajayer SR Jacob A Yang WL Zhou M Chaung W Wang P. Cold-inducible rna-binding protein is an important mediator of alcohol-induced brain inflammation. PloS One (2013) 8(11):e79430. doi: 10.1371/journal.pone.0079430 24223948 PMC3815202

[18] Tan C Gurien SD Royster W Aziz M Wang P. Extracellular cirp induces inflammation in alveolar type ii cells *Via* trem-1. Front Cell Dev Biol (2020) 8:579157. doi: 10.3389/fcell.2020.579157 32984356 PMC7484489

[19] Bolourani S Sari E Brenner M Wang P. Extracellular cirp induces an inflammatory phenotype in pulmonary fibroblasts *Via* Tlr4. Front Immunol (2021) 12:721970. doi: 10.3389/fimmu.2021.721970 34367191 PMC8342891

[20] Denning NL Aziz M Murao A Gurien SD Ochani M Prince JM. Extracellular cirp as an endogenous trem-1 ligand to fuel inflammation in sepsis. JCI Insight (2020) 5(5). doi: 10.1172/jci.insight.134172 PMC7141396 32027618

[21] Aziz M Brenner M Wang P. Extracellular cirp (Ecirp) and inflammation. J Leukoc Biol (2019) 106(1):133–46. doi: 10.1002/JLB.3MIR1118-443R PMC6597266 30645013

[22] Khan MM Yang WL Brenner M Bolognese AC Wang P. Cold-inducible rna-binding protein (Cirp) causes sepsis-associated acute lung injury *Via* induction of endoplasmic reticulum stress. Sci Rep (2017) 7:41363. doi: 10.1038/srep41363 28128330 PMC5269663

[23] Godwin A Yang WL Sharma A Khader A Wang Z Zhang F. Blocking cold-inducible rna-binding protein protects liver from ischemia-reperfusion injury. Shock (2015) 43(1):24–30. doi: 10.1097/SHK.0000000000000251 25186836 PMC4270919

[24] Lomberk G Urrutia R. The family feud: Turning off Sp1 by Sp1-like klf proteins. Biochem J (2005) 392(Pt 1):1–11. doi: 10.1042/BJ20051234 16266294 PMC1317658

[25] Imanishi M Imamura C Higashi C Yan W Negi S Futaki S. Zinc finger-zinc finger interaction between the transcription factors, gata-1 and Sp1. Biochem Biophys Res Commun (2010) 400(4):625–30. doi: 10.1016/j.bbrc.2010.08.116 20807505

[26] Lee JA Suh DC Kang JE Kim MH Park H Lee MN. Transcriptional activity of Sp1 is regulated by molecular interactions between the zinc finger DNA binding domain and the inhibitory domain with corepressors, and this interaction is modulated by mek. J Biol Chem (2005) 280(30):28061–71. doi: 10.1074/jbc.M414134200 15878880

[27] Su F Geng J Li X Qiao C Luo L Feng J. Sp1 promotes tumor angiogenesis and invasion by activating vegf expression in an acquired trastuzumabresistant ovarian cancer model. Oncol Rep (2017) 38(5):2677–84. doi: 10.3892/or.2017.5998 PMC5780020 29048687

[28] Xia CP Pan T Zhang N Guo JR Yang BW Zhang D. Sp1 promotes dental pulp stem cell osteoblastic differentiation through regulating noggin. Mol Cell Probes (2020) 50:101504. doi: 10.1016/j.mcp.2019.101504 31904417

[29] Cai LJ Tu L Li T Yang XL Ren YP Gu R. Up-regulation of microrna-375 ameliorates the damage of dopaminergic neurons, reduces oxidative stress and inflammation in parkinson’s disease by inhibiting Sp1. Aging (Albany NY) (2020) 12(1):672–89. doi: 10.18632/aging.102649 PMC6977707 31927536

[30] Sheng L Wu J Gong X Dong D Sun X. Sp1-induced upregulation of lncrna pandar predicts adverse phenotypes in retinoblastoma and regulates cell growth and apoptosis *in vitro* and *in vivo* . Gene (2018) 668:140–5. doi: 10.1016/j.gene.2018.05.065 29778422

[31] Baek Y Seo BN Jeong K Yoo H Lee S. Lifestyle, genomic types and non-communicable diseases in Korea: A protocol for the Korean medicine daejeon citizen cohort study (Kdcc). BMJ Open (2020) 10(4):e034499. doi: 10.1136/bmjopen-2019-034499 PMC7170630 32276954

[32] Delaneau O Marchini J. The 1000 genomes project consortium. integrating sequence and array data to create an improved 1000 genomes project haplotype reference panel. Nat Commun (2014) 5:3934. doi: 10.1038/ncomms4934 25653097 PMC4338501

[33] Howie BN Donnelly P Marchini J. A flexible and accurate genotype imputation method for the next generation of genome-wide association studies. PloS Genet (2009) 5(6):e1000529. doi: 10.1371/journal.pgen.1000529 19543373 PMC2689936

[34] Consortium EP. An integrated encyclopedia of DNA elements in the human genome. Nature (2012) 489(7414):57–74. doi: 10.1038/nature11247 22955616 PMC3439153

[35] Pruim RJ Welch RP Sanna S Teslovich TM Chines PS Gliedt TP. LocusZoom: regional visualization of genome-wide association scan results. Bioinformatics (2010) 26(18):2336–7. doi: 10.1093/bioinformatics/btq419 PMC2935401 20634204

[36] Consortium GT. The gtex consortium atlas of genetic regulatory effects across human tissues. Science (2020) 369(6509):1318–30. doi: 10.1126/science.aaz1776 PMC7737656 32913098

[37] Gualerzi CO Giuliodori AM Pon CL. Transcriptional and post-transcriptional control of cold-shock genes. J Mol Biol (2003) 331(3):527–39. doi: 10.1016/s0022-2836(03)00732-0 12899826

[38] McCarthy MI Abecasis GR Cardon LR Goldstein DB Little J Ioannidis JP. Genome-wide association studies for complex traits: Consensus, uncertainty and challenges. Nat Rev Genet (2008) 9(5):356–69. doi: 10.1038/nrg2344 18398418

[39] Visscher PM Wray NR Zhang Q Sklar P McCarthy MI Brown MA. 10 years of gwas discovery: Biology, function, and translation. Am J Hum Genet (2017) 101(1):5–22. doi: 10.1016/j.ajhg.2017.06.005 28686856 PMC5501872

[40] Perdomo-Sabogal A Nowick K. Genetic variation in human gene regulatory factors uncovers regulatory roles in local adaptation and disease. Genome Biol Evol (2019) 11(8):2178–93. doi: 10.1093/gbe/evz131 PMC6685493 31228201

[41] Albert FW Kruglyak L. The role of regulatory variation in complex traits and disease. Nat Rev Genet (2015) 16(4):197–212. doi: 10.1038/nrg3891 25707927

[42] Zhao JG Zhou L Jin JY Zhao Z Lan J Zhang YB. Antimicrobial activity-specific to gram-negative bacteria and immune modulation-mediated nf-kappab and Sp1 of a medaka beta-defensin. Dev Comp Immunol (2009) 33(4):624–37. doi: 10.1016/j.dci.2008.11.006 19084554

[43] Zhu HF Liu YP Liu DL Ma YD Hu ZY Wang XY. Role of Tgfbeta3-Smads-Sp1 axis in Dcr3-mediated immune escape of hepatocellular carcinoma. Oncogenesis (2019) 8(8):43. doi: 10.1038/s41389-019-0152-0 31409774 PMC6692328

[44] Chang R Chu Q Zheng W Zhang L Xu T. The Sp1-responsive microrna-15b negatively regulates rhabdovirus-triggered innate immune responses in lower vertebrates by targeting Tbk1. Front Immunol (2020) 11:625828. doi: 10.3389/fimmu.2020.625828 33584728 PMC7873567

[45] Yu Y Cao F Xiong Y Zhou H. Sp1 transcriptionally activates Nlrp6 inflammasome and induces immune evasion and radioresistance in glioma cells. Int Immunopharmacol (2021) 98:107858. doi: 10.1016/j.intimp.2021.107858 34147913

[46] Wu X Wan Q Wang J Hou P Zhang Q Wang Q. Epigenetic activation of lncrna Mir155hg mediated by promoter hypomethylation and Sp1 is correlated with immune infiltration in glioma. Onco Targets Ther (2022) 15:219–35. doi: 10.2147/OTT.S349078 PMC8922801 35299997

[47] Wang R Yang Y Wang H He Y Li C. Mir-29c protects against inflammation and apoptosis in parkinson’s disease model *in vivo* and *in vitro* by targeting Sp1. Clin Exp Pharmacol Physiol (2020) 47(3):372–82. doi: 10.1111/1440-1681.13212 31732967

[48] Kekuda R Saha P Sundaram U. Role of Sp1 and Hnf1 transcription factors in Sglt1 regulation during chronic intestinal inflammation. Am J Physiol Gastrointest Liver Physiol (2008) 294(6):G1354–61. doi: 10.1152/ajpgi.00080.2008 18339704

[49] Liu Y Liao R Qiang Z Zhang C. Pro-inflammatory cytokine-driven Pi3k/Akt/Sp1 signalling and H_2_S production facilitates the pathogenesis of severe acute pancreatitis. Biosci Rep (2017) 37(2):BSR20160483. doi: 10.1042/BSR20160483 28396512 PMC5408656

[50] Zhu CJ Wang QQ Zhou JL Liu HZ Hua F Yang HZ. The mineralocorticoid receptor-P38mapk-Nfkappab or erk-Sp1 signal pathways mediate aldosterone-stimulated inflammatory and profibrotic responses in rat vascular smooth muscle cells. Acta Pharmacol Sin (2012) 33(7):873–8. doi: 10.1038/aps.2012.36 PMC4011158 22659623

[51] Liu GX Li YQ Huang XR Wei L Chen HY Shi YJ. Disruption of Smad7 promotes ang ii-mediated renal inflammation and fibrosis via Sp1-TGF-β/Smad3-NF.κB-dependent mechanisms in mice. PloS One (2013) 8(1):e53573. doi: 10.1371/journal.pone.0053573 23301086 PMC3536757

[52] Plourde M Vohl M-C Bellis C Carless M Dyer T Dolley G. A variant in the Lrrfip1 gene is associated with adiposity and inflammation. Obesity (2012) 21(1):185–92. doi: 10.1038/oby.2012.181 23505185

[53] Stampanoni Bassi M Buttari F Simonelli I Gilio L Furlan R Finardi A. A single nucleotide ada genetic variant is associated to central inflammation and clinical presentation in Ms: Implications for cladribine treatment. Genes (Basel) (2020) 11(10):1152. doi: 10.3390/genes11101152 PMC7601054 33007809

[54] Pages G Pouyssegur J. Transcriptional regulation of the vascular endothelial growth factor gene–a concert of activating factors. Cardiovasc Res (2005) 65(3):564–73. doi: 10.1016/j.cardiores.2004.09.032 15664382

[55] Qiang X Yang WL Wu R Zhou M Jacob A Dong W. Cold-inducible RNA-binding protein (CIRP) triggers inflammatory responses in hemorrhagic shock and sepsis. Nat Med (2013) 19:1489–95. doi: 10.1038/nm.3368 PMC3826915 24097189

[56] Zhong P Peng J Yuan M Kong B Huang H. Cold-inducible RNA-binding protein (CIRP) in inflammatory diseases: molecular insights of its associated signalling pathways. Scand J Immunol (2021) 93(1):e12949. doi: 10.1111/sji.12949 32738154

[57] Godwin A Yang WL Sharma A Khader A Wang Z Zhang F. Blocking cold-inducible RNA-binding protein protects liver from ischemia-reperfusion injury. Shock (2015) 43(1):24–30. doi: 10.1097/SHK.0000000000000251 25186836 PMC4270919

[58] Aziz M Brenner M Wang P. (eCIRP) and inflammation. J Leukoc Biol (2019) 106:133–46. doi: 10.1002/JLB.3MIR1118-443R PMC6597266 30645013

[59] Zhong P Peng J Bian Z Huang H. The role of cold inducible RNA-binding protein in cardiac physiology and diseases. Front Pharmacol (2021) 12:610792. doi: 10.3389/fphar.2021.610792 33716740 PMC7943917

[60] Jang HH Lee HN Kim SY Hong S Lee WS. Expression of RNA-binding motif protein 3 (RBM3) and cold-inducible RNA-binding protein (CIRP) is associated with improved clinical outcome in patients with colon cancer. Anticancer Res (2017) 37:1779–85. doi: 10.21873/anticanres.11511 28373441

