# Supplementary figures and images for: Regulation of CIRP by genetic factors of SP1 related to cold sensitivity
Source: Front Immunol. 2022 Sep 16;13:994699. doi: 10.3389/fimmu.2022.994699 (PMC9524288; doi:10.3389/fimmu.2022.994699)

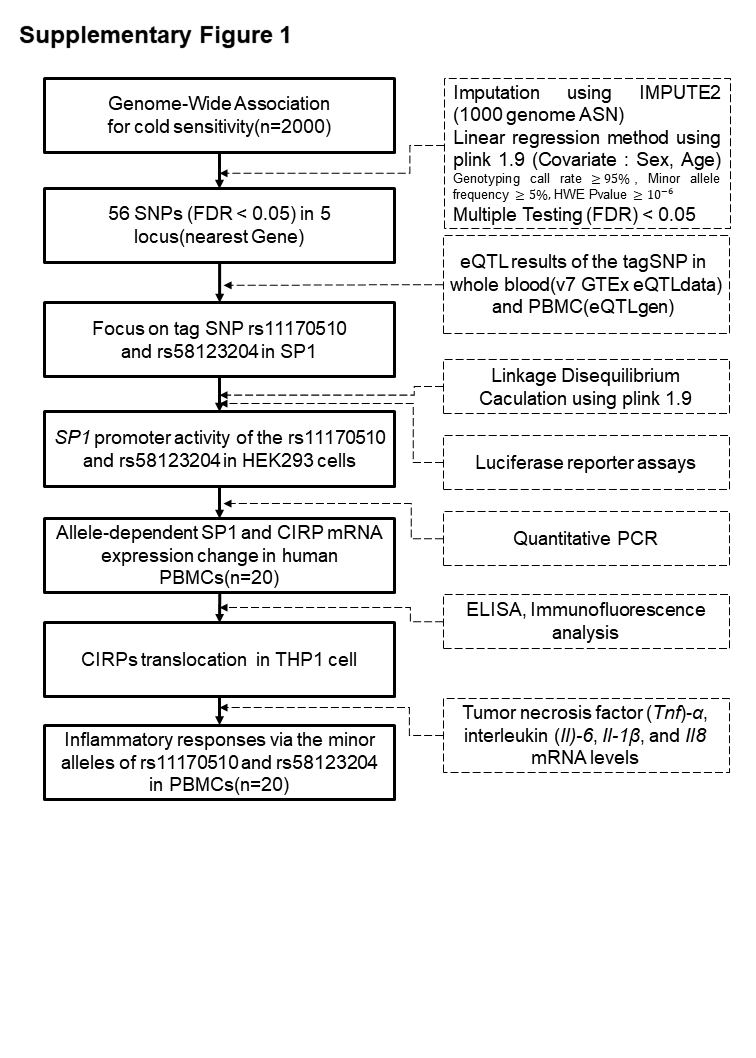

Supplement: Supplementary Figure 1 — Workflow of the study design. 2,000 Koreans were recruited and DNA was collected along with a questionnaire about cold sensitivity. The DNA was genotyped using a TPMRA chip and linear regression analysis was performed between genotype and cold sensitivity score. The changes in Sp1 and Cirp gene expression according to the cold sensitivity-related genetic loci were confirmed through an experimental verification experiment for SNPs that were statistically significant (FDR<0.05) from the results. [file Image_1.tif]

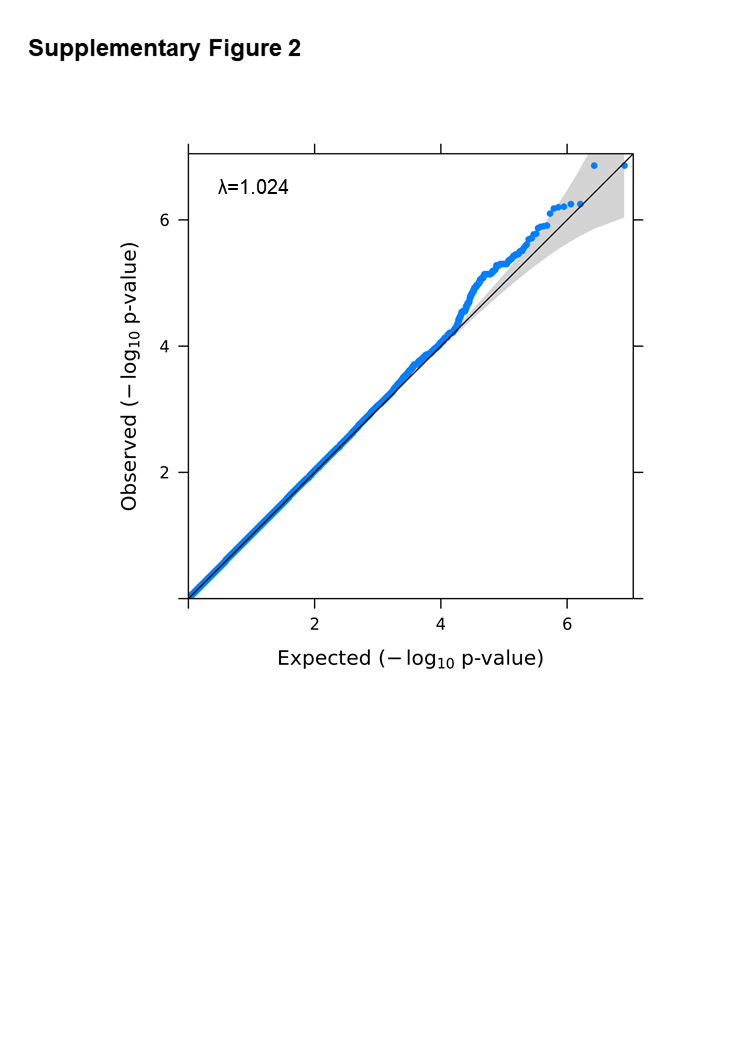

Supplement: Supplementary Figure 2 — QQ plot of p-values based on GWAS for the cold sensitivity. The results did not show any inflation of the test statistics (lambda = 1.024) [file Image_2.tif]

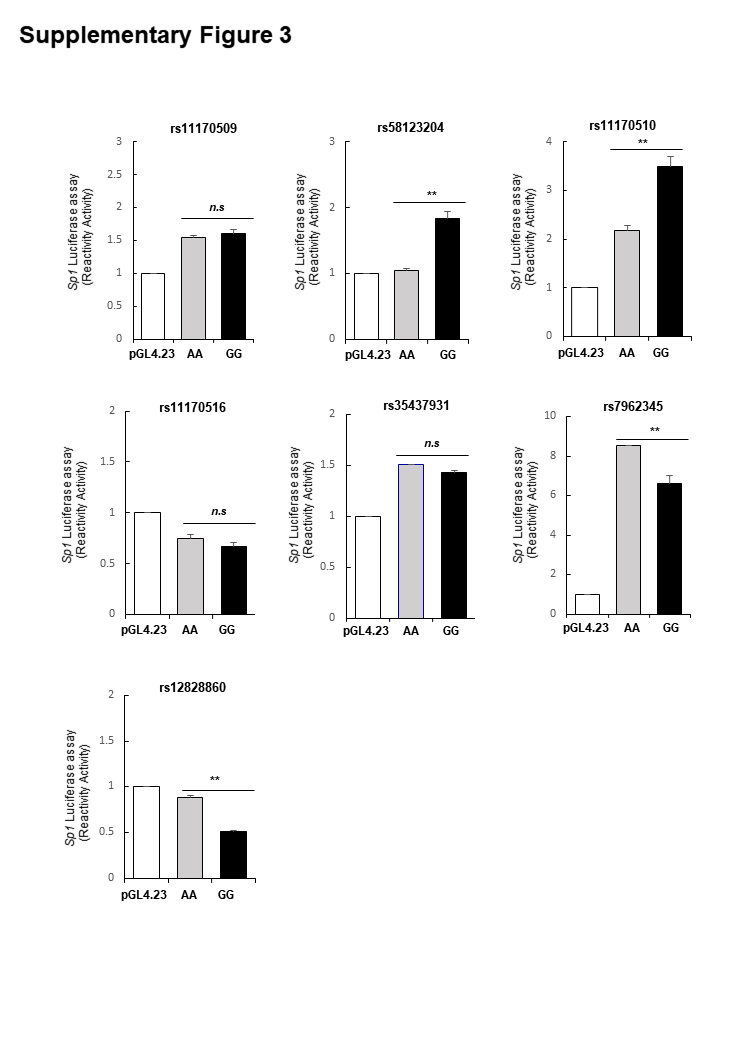

Supplement: Supplementary Figure 3 — Functional effects of SNPs rs11170509, rs11170516, rs35437931, rs7962345, rs12828860, rs11170510, and rs58123204. HEK293 cells were transfected with the mock vector (pGL4.23 vector), major A allele of the rs11170509, rs11170516, rs35437931, rs7962345, rs12828860, rs11170510, and rs58123204 or minor G allele of the rs11170509, rs11170516, rs35437931, rs7962345, rs12828860, rs11170510, and rs58123204-luciferase reporter constructs. Cells were subjected to a luciferase assay. Experiments were repeated at least three times. Graphs are indicative of the mean ± standard deviation (SD). **p < 0.01 (two-tailed unpaired Mann–Whitney). [file Image_3.tif]
