# Supplementary material for: Regulation of CIRP by genetic factors of SP1 related to cold sensitivity
Source: Front Immunol. 2022 Sep 16;13:994699. doi: 10.3389/fimmu.2022.994699 (PMC9524288; doi:10.3389/fimmu.2022.994699)
Supplement: Supplementary file 4 [file Table_1.docx]

Supplementary Table 1. Sample Information for GWAS

|  | Total (n=2000) | | Men (n=611) | | Women (n=1389) | | T test | Validated Data(n=20) | |
| --- | --- | --- | --- | --- | --- | --- | --- | --- | --- |
|  | mean | sd | mean | sd | mean | sd | p value | mean | sd |
| Height (cm) | 162.57 | 8.25 | 171.84 | 6.06 | 158.5 | 5.23 | 2.34E-259 | 161.06 | 6.88 |
| Weight(kg) | 64.72 | 12.53 | 76.13 | 11.28 | 59.7 | 9.35 | 3.47E-152 | 67.03 | 12.98 |
| Age | 44.32 | 6.85 | 43.34 | 7.3 | 44.76 | 6.59 | 3.92E-05 | 44.02 | 7.08 |
| Cold_Score | 22.86 | 6.06 | 19.67 | 5.02 | 24.26 | 5.95 | 6.68E-64 | 19.10 | 6.82 |
| BMI(kg/m^2^) | 24.36 | 3.63 | 25.75 | 3.31 | 23.75 | 3.6 | 3.46E-32 | 25.74 | 4.34 |
| SBP(mmHg) | 116.96 | 15.34 | 123.51 | 13.83 | 114.08 | 15.08 | 1.23E-39 | 120.48 | 16.81 |
| DBP(mmHg) | 73.5 | 12.08 | 80.4 | 11.22 | 70.46 | 11.15 | 1.04E-65 | 75.15 | 12.32 |
| BUN(mg/dL) | 13.4 | 3.46 | 14.65 | 3.56 | 12.86 | 3.27 | 3.12E-25 | 12.83 | 3.04 |
| Creatinine(mg/dL) | 0.72 | 0.26 | 0.89 | 0.13 | 0.64 | 0.27 | 4.70E-138 | 0.74 | 0.14 |
| F-Glucose(mg/dL) | 84.13 | 16.33 | 87.42 | 18.99 | 82.68 | 14.77 | 5.47E-08 | 82.25 | 9.73 |
| Insulin(mg/dL) | 6.1 | 4.35 | 6.86 | 5.09 | 5.77 | 3.94 | 2.75E-06 | 7.77 | 4.76 |
| Total Cholesterol(mg/dL) | 197.19 | 35.13 | 201.53 | 35.12 | 195.28 | 34.95 | 0.0003 | 182.45 | 32.58 |
| Triglyceride(mg/dL) | 132.25 | 124.03 | 184.32 | 164.9 | 109.35 | 91.98 | 2.41E-24 | 115.10 | 48.33 |
| HDL(mg/dL) | 56.82 | 13.95 | 49.88 | 11.37 | 59.87 | 13.89 | 3.25E-58 | 54.95 | 11.41 |
| LDL(mg/dL) | 120.03 | 32.8 | 125.16 | 33.52 | 117.78 | 32.21 | 4.98E-06 | 109.90 | 29.60 |
| CRP(mg/dL) | 1.24 | 2.77 | 1.23 | 1.94 | 1.25 | 3.06 | 0.874 | 1.81 | 1.79 |
| WBC(Thous/uL) | 5.46 | 1.54 | 5.9 | 1.52 | 5.26 | 1.5 | 1.94E-17 | 5.21 | 0.94 |
| RBC(Mil/uL) | 4.55 | 0.52 | 4.98 | 0.34 | 4.36 | 0.47 | 2.64E-181 | 4.50 | 0.33 |
| Hemoglobin(g/dL) | 13.83 | 1.59 | 15.37 | 0.9 | 13.15 | 1.33 | 5.44E-277 | 13.90 | 1.09 |
| Eosinophil Count | 168.4 | 122.37 | 200.34 | 148.66 | 154.35 | 105.75 | 9.12E-12 | 157.50 | 141.56 |
| HbA1c (%) | 5.48 | 0.68 | 5.58 | 0.68 | 5.43 | 0.68 | 6.57E-06 | 5.52 | 0.31 |
| r_GTP(IU/L) | 30.15 | 38.15 | 50.48 | 55.03 | 21.2 | 22.38 | 2.43E-33 | 42.85 | 31.35 |

BMI, Body Mass Index; SBP, Systolic Blood Pressure; DBP, Diastolic Blood Pressure; BUN, Blood Urea Nitrogen; Glucose, Fasting glucose; HDL, High Density Lipoprotein cholesterol; LDL, Low Density Lipoprotein cholesterol; CRP, C-Reactive Protein; WBC, White Blood Cell; RBC, Red Blood Cell; HbA1c, Hemoglobin A1c; r_GTP, γ-glutamyl transpeptidase

Supplementary Material

# Supplementary Table

**Supplementary Table 1**

Supplementary Table 1 shows statistical information for 2000 Koreans. 611 men and 1389 women were recruited, and the means and standard deviations of each item were obtained. It was found that there was a difference between men and women in most items.These results demonstrate the need to adjust for age and gender in the GWAS study.
