# Supplementary material for: Regulation of CIRP by genetic factors of SP1 related to cold sensitivity
Source: Front Immunol. 2022 Sep 16;13:994699. doi: 10.3389/fimmu.2022.994699 (PMC9524288; doi:10.3389/fimmu.2022.994699)
Supplement: Supplementary file 5 [file Table_2.docx]

Supplementary Table2. GWAS results for cold sensitivity in the 2000 samples.

| CHR | SNP | BP | BETA | L95 | U95 | SE | P | FDR | MAF | REF | Minor | Nearest Gene | Annotation |
| --- | --- | --- | --- | --- | --- | --- | --- | --- | --- | --- | --- | --- | --- |
| 10 | rs77101060 | 108393045 | -0.96 | -1.33 | -0.58 | 0.21 | 6.45E-06 | 0.043 | 0.24 | C | T | SORCS1 | intronic |
| 10 | rs75609190 | 108393116 | -0.96 | -1.33 | -0.58 | 0.21 | 6.45E-06 | 0.043 | 0.24 | A | C | SORCS1 | intronic |
| 10 | rs11192988 | 108403362 | -0.98 | -1.39 | -0.57 | 0.21 | 3.57E-06 | 0.039 | 0.25 | C | G | SORCS1 | intronic |
| 10 | rs11192989 | 108403873 | -0.99 | -1.40 | -0.57 | 0.21 | 3.11E-06 | 0.039 | 0.25 | C | T | SORCS1 | intronic |
| 10 | rs12221205 | 108406433 | -0.94 | -1.34 | -0.53 | 0.21 | 7.11E-06 | 0.045 | 0.25 | C | T | SORCS1 | intronic |
| 10 | rs12219036 | 108406455 | -0.94 | -1.34 | -0.53 | 0.21 | 7.11E-06 | 0.045 | 0.25 | A | T | SORCS1 | intronic |
| 10 | rs11193001 | 108414378 | -0.93 | -1.33 | -0.52 | 0.21 | 7.70E-06 | 0.047 | 0.25 | A | G | SORCS1 | intronic |
| 10 | rs821937 | 108446883 | -0.97 | -1.39 | -0.56 | 0.21 | 5.00E-06 | 0.039 | 0.23 | G | G | SORCS1 | intronic |
| 10 | rs821936 | 108447065 | -0.97 | -1.38 | -0.55 | 0.21 | 5.24E-06 | 0.039 | 0.23 | G | G | SORCS1 | intronic |
| 10 | rs821935 | 108447836 | -0.97 | -1.39 | -0.55 | 0.21 | 5.23E-06 | 0.039 | 0.23 | C | C | SORCS1 | intronic |
| 10 | rs1252035 | 108448200 | -1.27 | -1.77 | -0.77 | 0.25 | 6.68E-07 | 0.028 | 0.15 | C | C | SORCS1 | intronic |
| 10 | rs821934 | 108449004 | -0.97 | -1.39 | -0.55 | 0.21 | 5.23E-06 | 0.039 | 0.23 | C | C | SORCS1 | intronic |
| 10 | rs1252036 | 108453003 | -0.97 | -1.39 | -0.55 | 0.21 | 5.23E-06 | 0.039 | 0.23 | T | T | SORCS1 | intronic |
| 10 | rs821961 | 108453733 | -0.98 | -1.40 | -0.56 | 0.21 | 5.05E-06 | 0.039 | 0.23 | G | G | SORCS1 | intronic |
| 10 | rs821959 | 108454377 | -0.98 | -1.40 | -0.56 | 0.22 | 5.60E-06 | 0.04 | 0.23 | T | T | SORCS1 | intronic |
| 10 | rs821958 | 108455143 | -0.98 | -1.39 | -0.56 | 0.21 | 5.03E-06 | 0.039 | 0.23 | C | C | SORCS1 | intronic |
| 10 | rs821957 | 108456326 | -0.98 | -1.39 | -0.56 | 0.21 | 5.03E-06 | 0.039 | 0.23 | T | T | SORCS1 | intronic |
| 10 | rs821956 | 108456359 | -0.98 | -1.39 | -0.56 | 0.21 | 5.03E-06 | 0.039 | 0.23 | A | A | SORCS1 | intronic |
| 10 | rs821955 | 108456522 | -1.34 | -1.84 | -0.84 | 0.25 | 1.40E-07 | 0.015 | 0.15 | C | C | SORCS1 | intronic |
| 10 | rs703495 | 108457683 | -1.34 | -1.84 | -0.84 | 0.25 | 1.40E-07 | 0.015 | 0.15 | C | C | SORCS1 | intronic |
| 10 | rs703496 | 108459568 | -1.33 | -1.85 | -0.81 | 0.26 | 5.59E-07 | 0.028 | 0.13 | G | G | SORCS1 | 5upstream,intronic |
| 10 | rs821950 | 108460406 | -1.33 | -1.85 | -0.81 | 0.26 | 5.59E-07 | 0.028 | 0.13 | T | T | SORCS1 | 5upstream,intronic |
| 10 | rs1923325 | 108464633 | -0.99 | -1.41 | -0.57 | 0.21 | 3.75E-06 | 0.039 | 0.23 | C | C | SORCS1 | intronic |
| 10 | rs1322012 | 108465080 | -0.99 | -1.41 | -0.57 | 0.21 | 3.75E-06 | 0.039 | 0.23 | C | C | SORCS1 | intronic |
| 10 | rs2756231 | 108466412 | -1.29 | -1.83 | -0.75 | 0.28 | 2.81E-06 | 0.039 | 0.12 | C | C | SORCS1 | intronic |
| 10 | rs911579 | 108466888 | -0.99 | -1.41 | -0.57 | 0.21 | 4.13E-06 | 0.039 | 0.23 | T | T | SORCS1 | intronic |
| 10 | rs2756232 | 108469161 | -1 | -1.42 | -0.58 | 0.21 | 3.16E-06 | 0.039 | 0.23 | C | C | SORCS1 | intronic |
| 10 | rs821954 | 108470811 | -1 | -1.41 | -0.58 | 0.21 | 3.46E-06 | 0.039 | 0.23 | C | C | SORCS1 | intronic |
| 10 | rs821953 | 108470994 | -1 | -1.41 | -0.58 | 0.21 | 3.46E-06 | 0.039 | 0.23 | G | G | SORCS1 | intronic |
| 10 | rs1272085 | 108473077 | -1 | -1.42 | -0.58 | 0.21 | 3.57E-06 | 0.039 | 0.23 | G | G | SORCS1 | intronic |
| 10 | rs821949 | 108475616 | -0.98 | -1.40 | -0.56 | 0.21 | 4.99E-06 | 0.039 | 0.23 | T | T | SORCS1 | intronic |
| 10 | rs821946 | 108476622 | -0.98 | -1.40 | -0.56 | 0.21 | 4.99E-06 | 0.039 | 0.23 | G | G | SORCS1 | intronic |
| 10 | rs821944 | 108477569 | -0.98 | -1.40 | -0.56 | 0.21 | 4.99E-06 | 0.039 | 0.23 | T | T | SORCS1 | intronic |
| 10 | rs2756233 | 108479274 | -1.03 | -1.45 | -0.62 | 0.21 | 1.26E-06 | 0.039 | 0.24 | G | G | SORCS1 | intronic |
| 12 | rs11170509 | 53743050 | -0.95 | -1.36 | -0.54 | 0.21 | 6.17E-06 | 0.026 | 0.24 | C | T | SP1 | non-coding |
| 12 | rs11170510 | 53743734 | -0.95 | -1.36 | -0.54 | 0.21 | 5.25E-06 | 0.026 | 0.24 | A | G | SP1 | non-coding |
| 12 | rs11170516 | 53752692 | -0.94 | -1.35 | -0.53 | 0.21 | 6.79E-06 | 0.026 | 0.24 | G | A | SP1 | non-coding |
| 12 | rs35437931 | 53756354 | -0.93 | -1.34 | -0.52 | 0.21 | 8.32E-06 | 0.026 | 0.24 | T | C | SP1 | non-coding |
| 12 | rs12828860 | 53759803 | -0.93 | -1.34 | -0.52 | 0.21 | 8.56E-06 | 0.026 | 0.24 | G | C | SP1 | non-coding |
| 12 | rs58123204 | 53760162 | -0.98 | -1.40 | -0.55 | 0.22 | 6.95E-06 | 0.026 | 0.22 | A | G | SP1 | non-coding |
| 12 | rs7962345 | 53762887 | -1.07 | -1.49 | -0.65 | 0.21 | 6.13E-07 | 0.026 | 0.23 | C | C | SP1 | non-coding |
| 15 | rs12903035 | 26569608 | -0.95 | -1.35 | -0.55 | 0.21 | 4.26E-06 | 0.048 | 0.27 | A | A | lincRNA | non-coding |
| 15 | rs11853274 | 26572363 | -0.91 | -1.30 | -0.52 | 0.2 | 6.50E-06 | 0.048 | 0.28 | T | T | lincRNA | non-coding |
| 15 | rs12442453 | 26574726 | -0.88 | -1.26 | -0.51 | 0.19 | 4.15E-06 | 0.048 | 0.38 | C | C | lincRNA | non-coding |
| 15 | rs7171237 | 26575300 | -0.87 | -1.25 | -0.50 | 0.19 | 4.44E-06 | 0.048 | 0.37 | A | A | lincRNA | non-coding |
| 15 | rs6576562 | 26578036 | -0.9 | -1.27 | -0.53 | 0.19 | 1.97E-06 | 0.036 | 0.37 | GC | None | lincRNA | non-coding |
| 15 | rs6576563 | 26578079 | -0.96 | -1.33 | -0.58 | 0.19 | 6.35E-07 | 0.026 | 0.37 | C | C | lincRNA | non-coding |
| 15 | rs6576564 | 26578154 | -0.9 | -1.27 | -0.53 | 0.19 | 2.03E-06 | 0.048 | 0.37 | G | G | lincRNA | non-coding |
| 15 | rs7168118 | 26578376 | -0.91 | -1.28 | -0.54 | 0.19 | 1.66E-06 | 0.036 | 0.37 | A | A | lincRNA | non-coding |
| 15 | rs7168485 | 26578530 | -0.91 | -1.29 | -0.54 | 0.19 | 1.71E-06 | 0.036 | 0.38 | A | A | lincRNA | non-coding |
| 15 | rs4365262 | 26579982 | -0.92 | -1.29 | -0.55 | 0.19 | 1.23E-06 | 0.026 | 0.37 | A | A | lincRNA | non-coding |
| 15 | rs8039457 | 26580823 | -0.91 | -1.28 | -0.54 | 0.19 | 1.35E-06 | 0.032 | 0.37 | G | G | lincRNA | non-coding |
| 15 | rs8041307 | 26580842 | -0.92 | -1.29 | -0.55 | 0.19 | 1.29E-06 | 0.028 | 0.37 | A | A | lincRNA | non-coding |
| 15 | rs12717759 | 26581883 | -0.89 | -1.26 | -0.52 | 0.19 | 2.45E-06 | 0.048 | 0.38 | T | T | lincRNA | non-coding |
| 15 | rs7177522 | 26583425 | -0.88 | -1.25 | -0.51 | 0.19 | 3.10E-06 | 0.048 | 0.38 | A | A | lincRNA | non-coding |
| 15 | rs6576565 | 26584959 | -0.9 | -1.27 | -0.53 | 0.19 | 1.98E-06 | 0.048 | 0.38 | T | T | lincRNA | non-coding |

Abbreviations: Annotation, functional region; Beta, indicates the regression coefficients showing the increase or decrease in the cold score per minor allele; BP, base-pair position; CHR, Chromosome; L95, lower 95 confidence interval; U95, upper 95 confidence interval; FDR, adjusted p-value; MAF, minor allele frequency; Minor, minor allele; P, unadjusted p-value; REF, reference allele SE, Standard error of Beta; SNP, Single Nucleotide Polymorphism.

Supplementary Material

# Supplementary Table

**Supplementary Table 2.** Genome-wide association study on cold sensitivity for 2,000 Koreans. The cold scores indicate the results of BETA (Genetic Effects) (*p-*value, false discovery rate [FDR] < 0.05) obtained using linear regression adjusted for gender and age. The resulting single nucleotide polymorphisms (SNPs) were annotated with minor allele frequency, allele type, nearest gene, and functional region as shown in the table. SNP locations are indicated in accordance with the UCSC genome build hg19 version.
