# Supplementary material for: Regulation of CIRP by genetic factors of SP1 related to cold sensitivity
Source: Front Immunol. 2022 Sep 16;13:994699. doi: 10.3389/fimmu.2022.994699 (PMC9524288; doi:10.3389/fimmu.2022.994699)
Supplement: Supplementary file 6 [file Table_3.docx]

Supplementary Table3. The eQTL(SNP-Gene association test) results of significant SNPs related cold sensitivity in whole blood

| dbSNP | ene_id | gene_name | variant_id | tss_distance | ma_samples | maf | pval_nominal | slope | slope_se |
| --- | --- | --- | --- | --- | --- | --- | --- | --- | --- |
| rs77101060 | ENSG00000213260.3 | YWHAZP5 | 10_108393045_C_T_b37 | 946965 | 60 | 0.087 | 0.643 | 0.052 | 0.112 |
| rs75609190 | ENSG00000213260.3 | YWHAZP5 | 10_108393116_A_C_b37 | 947036 | 60 | 0.087 | 0.643 | 0.052 | 0.112 |
| rs11192988 | ENSG00000213260.3 | YWHAZP5 | 10_108403362_C_G_b37 | 957282 | 62 | 0.089 | 0.774 | 0.032 | 0.110 |
| rs11192989 | ENSG00000213260.3 | YWHAZP5 | 10_108403873_C_T_b37 | 957793 | 60 | 0.087 | 0.652 | 0.050 | 0.112 |
| rs12221205 | ENSG00000213260.3 | YWHAZP5 | 10_108406433_C_T_b37 | 960353 | 61 | 0.087 | 0.946 | -0.008 | 0.113 |
| rs12219036 | ENSG00000213260.3 | YWHAZP5 | 10_108406455_A_T_b37 | 960375 | 60 | 0.086 | 0.844 | 0.022 | 0.114 |
| rs11193001 | ENSG00000213260.3 | YWHAZP5 | 10_108414378_A_G_b37 | 968298 | 61 | 0.088 | 0.803 | 0.028 | 0.111 |
| rs11170509 | ENSG00000111481.5 | COPZ1 | 12_53743050_C_T_b37 | -951936 | 101 | 0.154 | 0.847 | 0.006 | 0.031 |
| rs11170509 | ENSG00000123405.9 | NFE2 | 12_53743050_C_T_b37 | -951855 | 101 | 0.154 | 0.567 | -0.013 | 0.023 |
| rs11170509 | ENSG00000135486.13 | HNRNPA1 | 12_53743050_C_T_b37 | -930927 | 101 | 0.154 | 0.735 | 0.012 | 0.037 |
| rs11170509 | ENSG00000094916.9 | CBX5 | 12_53743050_C_T_b37 | -930836 | 101 | 0.154 | 0.240 | 0.048 | 0.041 |
| rs11170509 | ENSG00000257596.1 | RP11-968A15.2 | 12_53743050_C_T_b37 | -913349 | 101 | 0.154 | 0.975 | -0.002 | 0.070 |
| rs11170509 | ENSG00000123415.10 | SMUG1 | 12_53743050_C_T_b37 | -839728 | 101 | 0.154 | 0.156 | -0.047 | 0.033 |
| rs11170509 | ENSG00000250742.1 | RP11-834C11.4 | 12_53743050_C_T_b37 | -776832 | 101 | 0.154 | 0.702 | 0.034 | 0.089 |
| rs11170509 | ENSG00000250654.3 | RP11-834C11.7 | 12_53743050_C_T_b37 | -732637 | 101 | 0.154 | 0.247 | 0.095 | 0.082 |
| rs11170509 | ENSG00000172789.3 | HOXC5 | 12_53743050_C_T_b37 | -683587 | 101 | 0.154 | 0.301 | 0.079 | 0.077 |
| rs11170509 | ENSG00000180818.4 | HOXC10 | 12_53743050_C_T_b37 | -635818 | 101 | 0.154 | 0.420 | -0.057 | 0.071 |
| rs11170509 | ENSG00000012822.11 | CALCOCO1 | 12_53743050_C_T_b37 | -378479 | 101 | 0.154 | 0.148 | -0.054 | 0.037 |
| rs11170509 | ENSG00000135390.13 | ATP5G2 | 12_53743050_C_T_b37 | -328142 | 101 | 0.154 | 0.884 | 0.003 | 0.023 |
| rs11170509 | ENSG00000170653.14 | ATF7 | 12_53743050_C_T_b37 | -277149 | 101 | 0.154 | 0.439 | -0.027 | 0.034 |
| rs11170509 | ENSG00000139574.7 | NPFF | 12_53743050_C_T_b37 | -158372 | 101 | 0.154 | 0.105 | -0.051 | 0.031 |
| rs11170509 | ENSG00000139546.6 | TARBP2 | 12_53743050_C_T_b37 | -151671 | 101 | 0.154 | 0.004 | -0.100 | 0.035 |
| rs11170509 | ENSG00000270175.1 | RP11-793H13.11 | 12_53743050_C_T_b37 | -151654 | 101 | 0.154 | 0.025 | -0.183 | 0.081 |
| rs11170509 | ENSG00000139625.8 | MAP3K12 | 12_53743050_C_T_b37 | -150797 | 101 | 0.154 | 0.109 | -0.066 | 0.041 |
| rs11170509 | ENSG00000197111.11 | PCBP2 | 12_53743050_C_T_b37 | -102836 | 101 | 0.154 | 0.180 | -0.043 | 0.032 |
| rs11170509 | ENSG00000205352.6 | PRR13 | 12_53743050_C_T_b37 | -92339 | 101 | 0.154 | 0.013 | 0.043 | 0.017 |
| rs11170509 | ENSG00000135409.6 | AMHR2 | 12_53743050_C_T_b37 | -74589 | 101 | 0.154 | 0.805 | 0.022 | 0.091 |
| rs11170509 | ENSG00000185591.5 | SP1 | 12_53743050_C_T_b37 | -30910 | 101 | 0.154 | 2.38454E-08 | 0.121 | 0.021 |
| rs11170509 | ENSG00000094914.8 | AAAS | 12_53743050_C_T_b37 | 24402 | 101 | 0.154 | 0.813 | -0.008 | 0.033 |
| rs11170509 | ENSG00000257605.1 | RP11-680A11.5 | 12_53743050_C_T_b37 | 48952 | 101 | 0.154 | 0.031 | 0.147 | 0.068 |
| rs11170509 | ENSG00000139637.9 | C12orf10 | 12_53743050_C_T_b37 | 49580 | 101 | 0.154 | 0.537 | -0.023 | 0.036 |
| rs11170509 | ENSG00000123349.9 | PFDN5 | 12_53743050_C_T_b37 | 53975 | 101 | 0.154 | 0.527 | -0.016 | 0.026 |
| rs11170509 | ENSG00000135476.7 | ESPL1 | 12_53743050_C_T_b37 | 80967 | 101 | 0.154 | 0.340 | -0.034 | 0.035 |
| rs11170509 | ENSG00000182544.8 | MFSD5 | 12_53743050_C_T_b37 | 98015 | 101 | 0.154 | 0.131 | -0.041 | 0.027 |
| rs11170509 | ENSG00000172819.12 | RARG | 12_53743050_C_T_b37 | 116286 | 101 | 0.154 | 0.320 | 0.044 | 0.044 |
| rs11170509 | ENSG00000139626.11 | ITGB7 | 12_53743050_C_T_b37 | 141959 | 101 | 0.154 | 0.711 | 0.009 | 0.025 |
| rs11170509 | ENSG00000139631.14 | CSAD | 12_53743050_C_T_b37 | 167915 | 101 | 0.154 | 0.224 | 0.038 | 0.031 |
| rs11170509 | ENSG00000139651.9 | ZNF740 | 12_53743050_C_T_b37 | 168566 | 101 | 0.154 | 0.952 | 0.002 | 0.034 |
| rs11170509 | ENSG00000257790.1 | RP11-1136G11.6 | 12_53743050_C_T_b37 | 195994 | 101 | 0.154 | 0.030 | -0.173 | 0.079 |
| rs11170509 | ENSG00000167780.7 | SOAT2 | 12_53743050_C_T_b37 | 245748 | 101 | 0.154 | 0.345 | -0.066 | 0.070 |
| rs11170509 | ENSG00000167779.3 | IGFBP6 | 12_53743050_C_T_b37 | 251830 | 101 | 0.154 | 0.132 | 0.081 | 0.054 |
| rs11170509 | ENSG00000167778.4 | SPRYD3 | 12_53743050_C_T_b37 | 269846 | 101 | 0.154 | 0.624 | 0.012 | 0.025 |
| rs11170509 | ENSG00000257337.2 | RP11-983P16.4 | 12_53743050_C_T_b37 | 295692 | 101 | 0.154 | 0.645 | 0.023 | 0.050 |
| rs11170509 | ENSG00000111077.13 | TENC1 | 12_53743050_C_T_b37 | 302215 | 101 | 0.154 | 0.137 | 0.060 | 0.040 |
| rs11170509 | ENSG00000063046.13 | EIF4B | 12_53743050_C_T_b37 | 343108 | 101 | 0.154 | 0.492 | -0.022 | 0.032 |
| rs11170509 | ENSG00000170421.7 | KRT8 | 12_53743050_C_T_b37 | 399346 | 101 | 0.154 | 0.818 | -0.014 | 0.062 |
| rs11170509 | ENSG00000111057.6 | KRT18 | 12_53743050_C_T_b37 | 400395 | 101 | 0.154 | 0.121 | 0.087 | 0.056 |
| rs11170509 | ENSG00000170423.8 | KRT78 | 12_53743050_C_T_b37 | 500174 | 101 | 0.154 | 0.888 | 0.012 | 0.084 |
| rs11170509 | ENSG00000185640.5 | KRT79 | 12_53743050_C_T_b37 | 514971 | 101 | 0.154 | 0.460 | -0.065 | 0.088 |
| rs11170509 | ENSG00000170477.8 | KRT4 | 12_53743050_C_T_b37 | 534715 | 101 | 0.154 | 0.218 | 0.081 | 0.066 |
| rs11170509 | ENSG00000189182.5 | KRT77 | 12_53743050_C_T_b37 | 645803 | 101 | 0.154 | 0.593 | 0.052 | 0.097 |
| rs11170509 | ENSG00000167768.4 | KRT1 | 12_53743050_C_T_b37 | 668859 | 101 | 0.154 | 0.511 | 0.035 | 0.054 |
| rs11170509 | ENSG00000172867.3 | KRT2 | 12_53743050_C_T_b37 | 697102 | 101 | 0.154 | 0.653 | 0.037 | 0.082 |
| rs11170509 | ENSG00000186049.4 | KRT73 | 12_53743050_C_T_b37 | 730707 | 101 | 0.154 | 0.985 | 0.001 | 0.072 |
| rs11170509 | ENSG00000257495.1 | RP11-641A6.2 | 12_53743050_C_T_b37 | 747727 | 101 | 0.154 | 0.244 | -0.072 | 0.062 |
| rs11170509 | ENSG00000170486.6 | KRT72 | 12_53743050_C_T_b37 | 747800 | 101 | 0.154 | 0.993 | -0.001 | 0.082 |
| rs11170509 | ENSG00000186081.7 | KRT5 | 12_53743050_C_T_b37 | 828579 | 101 | 0.154 | 0.820 | 0.017 | 0.075 |
| rs11170509 | ENSG00000205420.6 | KRT6A | 12_53743050_C_T_b37 | 856009 | 101 | 0.154 | 0.373 | 0.061 | 0.068 |
| rs11170509 | ENSG00000170465.9 | KRT6C | 12_53743050_C_T_b37 | 875481 | 101 | 0.154 | 0.593 | 0.040 | 0.076 |
| rs11170509 | ENSG00000185479.5 | KRT6B | 12_53743050_C_T_b37 | 897140 | 101 | 0.154 | 0.870 | -0.014 | 0.084 |
| rs11170510 | ENSG00000111481.5 | COPZ1 | 12_53743734_A_G_b37 | -951252 | 101 | 0.154 | 0.935 | -0.003 | 0.031 |
| rs11170510 | ENSG00000123405.9 | NFE2 | 12_53743734_A_G_b37 | -951171 | 101 | 0.154 | 0.484 | -0.016 | 0.023 |
| rs11170510 | ENSG00000135486.13 | HNRNPA1 | 12_53743734_A_G_b37 | -930243 | 101 | 0.154 | 0.788 | 0.010 | 0.037 |
| rs11170510 | ENSG00000094916.9 | CBX5 | 12_53743734_A_G_b37 | -930152 | 101 | 0.154 | 0.250 | 0.047 | 0.041 |
| rs11170510 | ENSG00000257596.1 | RP11-968A15.2 | 12_53743734_A_G_b37 | -912665 | 101 | 0.154 | 0.930 | 0.006 | 0.070 |
| rs11170510 | ENSG00000123415.10 | SMUG1 | 12_53743734_A_G_b37 | -839044 | 101 | 0.154 | 0.159 | -0.046 | 0.033 |
| rs11170510 | ENSG00000250742.1 | RP11-834C11.4 | 12_53743734_A_G_b37 | -776148 | 101 | 0.154 | 0.752 | 0.028 | 0.090 |
| rs11170510 | ENSG00000250654.3 | RP11-834C11.7 | 12_53743734_A_G_b37 | -731953 | 101 | 0.154 | 0.294 | 0.086 | 0.082 |
| rs11170510 | ENSG00000172789.3 | HOXC5 | 12_53743734_A_G_b37 | -682903 | 101 | 0.154 | 0.350 | 0.072 | 0.077 |
| rs11170510 | ENSG00000180818.4 | HOXC10 | 12_53743734_A_G_b37 | -635134 | 101 | 0.154 | 0.506 | -0.047 | 0.071 |
| rs11170510 | ENSG00000012822.11 | CALCOCO1 | 12_53743734_A_G_b37 | -377795 | 101 | 0.154 | 0.103 | -0.061 | 0.037 |
| rs11170510 | ENSG00000135390.13 | ATP5G2 | 12_53743734_A_G_b37 | -327458 | 101 | 0.154 | 0.992 | 0.000 | 0.023 |
| rs11170510 | ENSG00000170653.14 | ATF7 | 12_53743734_A_G_b37 | -276465 | 101 | 0.154 | 0.442 | -0.026 | 0.034 |
| rs11170510 | ENSG00000139574.7 | NPFF | 12_53743734_A_G_b37 | -157688 | 101 | 0.154 | 0.101 | -0.051 | 0.031 |
| rs11170510 | ENSG00000139546.6 | TARBP2 | 12_53743734_A_G_b37 | -150987 | 101 | 0.154 | 0.003 | -0.103 | 0.035 |
| rs11170510 | ENSG00000270175.1 | RP11-793H13.11 | 12_53743734_A_G_b37 | -150970 | 101 | 0.154 | 0.030 | -0.178 | 0.081 |
| rs11170510 | ENSG00000139625.8 | MAP3K12 | 12_53743734_A_G_b37 | -150113 | 101 | 0.154 | 0.118 | -0.064 | 0.041 |
| rs11170510 | ENSG00000197111.11 | PCBP2 | 12_53743734_A_G_b37 | -102152 | 101 | 0.154 | 0.208 | -0.040 | 0.032 |
| rs11170510 | ENSG00000205352.6 | PRR13 | 12_53743734_A_G_b37 | -91655 | 101 | 0.154 | 0.011 | 0.044 | 0.017 |
| rs11170510 | ENSG00000135409.6 | AMHR2 | 12_53743734_A_G_b37 | -73905 | 101 | 0.154 | 0.923 | 0.009 | 0.091 |
| rs11170510 | ENSG00000185591.5 | SP1 | 12_53743734_A_G_b37 | -30226 | 101 | 0.154 | 3.9845E-08 | 0.119 | 0.021 |
| rs11170510 | ENSG00000094914.8 | AAAS | 12_53743734_A_G_b37 | 25086 | 101 | 0.154 | 0.785 | -0.009 | 0.033 |
| rs11170510 | ENSG00000257605.1 | RP11-680A11.5 | 12_53743734_A_G_b37 | 49636 | 101 | 0.154 | 0.036 | 0.143 | 0.068 |
| rs11170510 | ENSG00000139637.9 | C12orf10 | 12_53743734_A_G_b37 | 50264 | 101 | 0.154 | 0.446 | -0.028 | 0.036 |
| rs11170510 | ENSG00000123349.9 | PFDN5 | 12_53743734_A_G_b37 | 54659 | 101 | 0.154 | 0.431 | -0.020 | 0.026 |
| rs11170510 | ENSG00000135476.7 | ESPL1 | 12_53743734_A_G_b37 | 81651 | 101 | 0.154 | 0.354 | -0.033 | 0.035 |
| rs11170510 | ENSG00000182544.8 | MFSD5 | 12_53743734_A_G_b37 | 98699 | 101 | 0.154 | 0.111 | -0.044 | 0.027 |
| rs11170510 | ENSG00000172819.12 | RARG | 12_53743734_A_G_b37 | 116970 | 101 | 0.154 | 0.337 | 0.042 | 0.044 |
| rs11170510 | ENSG00000139626.11 | ITGB7 | 12_53743734_A_G_b37 | 142643 | 101 | 0.154 | 0.670 | 0.011 | 0.025 |
| rs11170510 | ENSG00000139631.14 | CSAD | 12_53743734_A_G_b37 | 168599 | 101 | 0.154 | 0.255 | 0.036 | 0.031 |
| rs11170510 | ENSG00000139651.9 | ZNF740 | 12_53743734_A_G_b37 | 169250 | 101 | 0.154 | 0.939 | 0.003 | 0.034 |
| rs11170510 | ENSG00000257790.1 | RP11-1136G11.6 | 12_53743734_A_G_b37 | 196678 | 101 | 0.154 | 0.036 | -0.168 | 0.079 |
| rs11170510 | ENSG00000167780.7 | SOAT2 | 12_53743734_A_G_b37 | 246432 | 101 | 0.154 | 0.410 | -0.058 | 0.070 |
| rs11170510 | ENSG00000167779.3 | IGFBP6 | 12_53743734_A_G_b37 | 252514 | 101 | 0.154 | 0.145 | 0.079 | 0.054 |
| rs11170510 | ENSG00000167778.4 | SPRYD3 | 12_53743734_A_G_b37 | 270530 | 101 | 0.154 | 0.569 | 0.014 | 0.025 |
| rs11170510 | ENSG00000257337.2 | RP11-983P16.4 | 12_53743734_A_G_b37 | 296376 | 101 | 0.154 | 0.647 | 0.023 | 0.050 |
| rs11170510 | ENSG00000111077.13 | TENC1 | 12_53743734_A_G_b37 | 302899 | 101 | 0.154 | 0.139 | 0.059 | 0.040 |
| rs11170510 | ENSG00000063046.13 | EIF4B | 12_53743734_A_G_b37 | 343792 | 101 | 0.154 | 0.500 | -0.021 | 0.032 |
| rs11170510 | ENSG00000170421.7 | KRT8 | 12_53743734_A_G_b37 | 400030 | 101 | 0.154 | 0.779 | -0.017 | 0.062 |
| rs11170510 | ENSG00000111057.6 | KRT18 | 12_53743734_A_G_b37 | 401079 | 101 | 0.154 | 0.128 | 0.086 | 0.056 |
| rs11170510 | ENSG00000170423.8 | KRT78 | 12_53743734_A_G_b37 | 500858 | 101 | 0.154 | 0.908 | 0.010 | 0.084 |
| rs11170510 | ENSG00000185640.5 | KRT79 | 12_53743734_A_G_b37 | 515655 | 101 | 0.154 | 0.359 | -0.081 | 0.088 |
| rs11170510 | ENSG00000170477.8 | KRT4 | 12_53743734_A_G_b37 | 535399 | 101 | 0.154 | 0.232 | 0.079 | 0.066 |
| rs11170510 | ENSG00000189182.5 | KRT77 | 12_53743734_A_G_b37 | 646487 | 101 | 0.154 | 0.547 | 0.058 | 0.097 |
| rs11170510 | ENSG00000167768.4 | KRT1 | 12_53743734_A_G_b37 | 669543 | 101 | 0.154 | 0.464 | 0.039 | 0.054 |
| rs11170510 | ENSG00000172867.3 | KRT2 | 12_53743734_A_G_b37 | 697786 | 101 | 0.154 | 0.668 | 0.035 | 0.082 |
| rs11170510 | ENSG00000186049.4 | KRT73 | 12_53743734_A_G_b37 | 731391 | 101 | 0.154 | 0.858 | 0.013 | 0.072 |
| rs11170510 | ENSG00000257495.1 | RP11-641A6.2 | 12_53743734_A_G_b37 | 748411 | 101 | 0.154 | 0.277 | -0.068 | 0.062 |
| rs11170510 | ENSG00000170486.6 | KRT72 | 12_53743734_A_G_b37 | 748484 | 101 | 0.154 | 0.933 | 0.007 | 0.082 |
| rs11170510 | ENSG00000186081.7 | KRT5 | 12_53743734_A_G_b37 | 829263 | 101 | 0.154 | 0.831 | 0.016 | 0.075 |
| rs11170510 | ENSG00000205420.6 | KRT6A | 12_53743734_A_G_b37 | 856693 | 101 | 0.154 | 0.374 | 0.061 | 0.068 |
| rs11170510 | ENSG00000170465.9 | KRT6C | 12_53743734_A_G_b37 | 876165 | 101 | 0.154 | 0.648 | 0.035 | 0.076 |
| rs11170510 | ENSG00000185479.5 | KRT6B | 12_53743734_A_G_b37 | 897824 | 101 | 0.154 | 0.952 | -0.005 | 0.084 |
| rs11170516 | ENSG00000258086.1 | RP11-753H16.5 | 12_53752692_G_A_b37 | -994884 | 100 | 0.152 | 0.008 | -0.189 | 0.071 |
| rs11170516 | ENSG00000111481.5 | COPZ1 | 12_53752692_G_A_b37 | -942294 | 100 | 0.152 | 0.932 | -0.003 | 0.031 |
| rs11170516 | ENSG00000123405.9 | NFE2 | 12_53752692_G_A_b37 | -942213 | 100 | 0.152 | 0.650 | -0.010 | 0.023 |
| rs11170516 | ENSG00000135486.13 | HNRNPA1 | 12_53752692_G_A_b37 | -921285 | 100 | 0.152 | 0.916 | 0.004 | 0.036 |
| rs11170516 | ENSG00000094916.9 | CBX5 | 12_53752692_G_A_b37 | -921194 | 100 | 0.152 | 0.191 | 0.053 | 0.041 |
| rs11170516 | ENSG00000257596.1 | RP11-968A15.2 | 12_53752692_G_A_b37 | -903707 | 100 | 0.152 | 0.751 | 0.022 | 0.070 |
| rs11170516 | ENSG00000123415.10 | SMUG1 | 12_53752692_G_A_b37 | -830086 | 100 | 0.152 | 0.100 | -0.054 | 0.033 |
| rs11170516 | ENSG00000250742.1 | RP11-834C11.4 | 12_53752692_G_A_b37 | -767190 | 100 | 0.152 | 0.376 | 0.079 | 0.089 |
| rs11170516 | ENSG00000250654.3 | RP11-834C11.7 | 12_53752692_G_A_b37 | -722995 | 100 | 0.152 | 0.183 | 0.108 | 0.081 |
| rs11170516 | ENSG00000172789.3 | HOXC5 | 12_53752692_G_A_b37 | -673945 | 100 | 0.152 | 0.169 | 0.105 | 0.076 |
| rs11170516 | ENSG00000180818.4 | HOXC10 | 12_53752692_G_A_b37 | -626176 | 100 | 0.152 | 0.692 | -0.028 | 0.071 |
| rs11170516 | ENSG00000012822.11 | CALCOCO1 | 12_53752692_G_A_b37 | -368837 | 100 | 0.152 | 0.102 | -0.060 | 0.037 |
| rs11170516 | ENSG00000135390.13 | ATP5G2 | 12_53752692_G_A_b37 | -318500 | 100 | 0.152 | 0.817 | -0.005 | 0.023 |
| rs11170516 | ENSG00000170653.14 | ATF7 | 12_53752692_G_A_b37 | -267507 | 100 | 0.152 | 0.431 | -0.027 | 0.034 |
| rs11170516 | ENSG00000139574.7 | NPFF | 12_53752692_G_A_b37 | -148730 | 100 | 0.152 | 0.075 | -0.055 | 0.031 |
| rs11170516 | ENSG00000139546.6 | TARBP2 | 12_53752692_G_A_b37 | -142029 | 100 | 0.152 | 0.009 | -0.091 | 0.035 |
| rs11170516 | ENSG00000270175.1 | RP11-793H13.11 | 12_53752692_G_A_b37 | -142012 | 100 | 0.152 | 0.023 | -0.184 | 0.081 |
| rs11170516 | ENSG00000139625.8 | MAP3K12 | 12_53752692_G_A_b37 | -141155 | 100 | 0.152 | 0.078 | -0.072 | 0.041 |
| rs11170516 | ENSG00000197111.11 | PCBP2 | 12_53752692_G_A_b37 | -93194 | 100 | 0.152 | 0.179 | -0.043 | 0.032 |
| rs11170516 | ENSG00000205352.6 | PRR13 | 12_53752692_G_A_b37 | -82697 | 100 | 0.152 | 0.012 | 0.043 | 0.017 |
| rs11170516 | ENSG00000135409.6 | AMHR2 | 12_53752692_G_A_b37 | -64947 | 100 | 0.152 | 0.834 | 0.019 | 0.090 |
| rs11170516 | ENSG00000185591.5 | SP1 | 12_53752692_G_A_b37 | -21268 | 100 | 0.152 | 1.62099E-08 | 0.121 | 0.021 |
| rs11170516 | ENSG00000094914.8 | AAAS | 12_53752692_G_A_b37 | 34044 | 100 | 0.152 | 0.957 | -0.002 | 0.032 |
| rs11170516 | ENSG00000257605.1 | RP11-680A11.5 | 12_53752692_G_A_b37 | 58594 | 100 | 0.152 | 0.035 | 0.143 | 0.067 |
| rs11170516 | ENSG00000139637.9 | C12orf10 | 12_53752692_G_A_b37 | 59222 | 100 | 0.152 | 0.387 | -0.031 | 0.036 |
| rs11170516 | ENSG00000123349.9 | PFDN5 | 12_53752692_G_A_b37 | 63617 | 100 | 0.152 | 0.615 | -0.013 | 0.026 |
| rs11170516 | ENSG00000135476.7 | ESPL1 | 12_53752692_G_A_b37 | 90609 | 100 | 0.152 | 0.265 | -0.039 | 0.035 |
| rs11170516 | ENSG00000182544.8 | MFSD5 | 12_53752692_G_A_b37 | 107657 | 100 | 0.152 | 0.123 | -0.042 | 0.027 |
| rs11170516 | ENSG00000172819.12 | RARG | 12_53752692_G_A_b37 | 125928 | 100 | 0.152 | 0.259 | 0.049 | 0.043 |
| rs11170516 | ENSG00000139626.11 | ITGB7 | 12_53752692_G_A_b37 | 151601 | 100 | 0.152 | 0.757 | 0.008 | 0.025 |
| rs11170516 | ENSG00000139631.14 | CSAD | 12_53752692_G_A_b37 | 177557 | 100 | 0.152 | 0.165 | 0.043 | 0.031 |
| rs11170516 | ENSG00000139651.9 | ZNF740 | 12_53752692_G_A_b37 | 178208 | 100 | 0.152 | 0.702 | 0.013 | 0.033 |
| rs11170516 | ENSG00000257790.1 | RP11-1136G11.6 | 12_53752692_G_A_b37 | 205636 | 100 | 0.152 | 0.062 | -0.148 | 0.079 |
| rs11170516 | ENSG00000167780.7 | SOAT2 | 12_53752692_G_A_b37 | 255390 | 100 | 0.152 | 0.280 | -0.075 | 0.070 |
| rs11170516 | ENSG00000167779.3 | IGFBP6 | 12_53752692_G_A_b37 | 261472 | 100 | 0.152 | 0.085 | 0.092 | 0.054 |
| rs11170516 | ENSG00000167778.4 | SPRYD3 | 12_53752692_G_A_b37 | 279488 | 100 | 0.152 | 0.473 | 0.018 | 0.025 |
| rs11170516 | ENSG00000257337.2 | RP11-983P16.4 | 12_53752692_G_A_b37 | 305334 | 100 | 0.152 | 0.602 | 0.026 | 0.049 |
| rs11170516 | ENSG00000111077.13 | TENC1 | 12_53752692_G_A_b37 | 311857 | 100 | 0.152 | 0.306 | 0.041 | 0.040 |
| rs11170516 | ENSG00000063046.13 | EIF4B | 12_53752692_G_A_b37 | 352750 | 100 | 0.152 | 0.412 | -0.026 | 0.031 |
| rs11170516 | ENSG00000170421.7 | KRT8 | 12_53752692_G_A_b37 | 408988 | 100 | 0.152 | 0.819 | -0.014 | 0.062 |
| rs11170516 | ENSG00000111057.6 | KRT18 | 12_53752692_G_A_b37 | 410037 | 100 | 0.152 | 0.138 | 0.083 | 0.056 |
| rs11170516 | ENSG00000170423.8 | KRT78 | 12_53752692_G_A_b37 | 509816 | 100 | 0.152 | 0.833 | 0.018 | 0.084 |
| rs11170516 | ENSG00000185640.5 | KRT79 | 12_53752692_G_A_b37 | 524613 | 100 | 0.152 | 0.610 | -0.045 | 0.087 |
| rs11170516 | ENSG00000170477.8 | KRT4 | 12_53752692_G_A_b37 | 544357 | 100 | 0.152 | 0.187 | 0.087 | 0.065 |
| rs11170516 | ENSG00000189182.5 | KRT77 | 12_53752692_G_A_b37 | 655445 | 100 | 0.152 | 0.722 | 0.034 | 0.096 |
| rs11170516 | ENSG00000167768.4 | KRT1 | 12_53752692_G_A_b37 | 678501 | 100 | 0.152 | 0.215 | 0.066 | 0.053 |
| rs11170516 | ENSG00000172867.3 | KRT2 | 12_53752692_G_A_b37 | 706744 | 100 | 0.152 | 0.672 | 0.035 | 0.082 |
| rs11170516 | ENSG00000186049.4 | KRT73 | 12_53752692_G_A_b37 | 740349 | 100 | 0.152 | 0.919 | 0.007 | 0.072 |
| rs11170516 | ENSG00000257495.1 | RP11-641A6.2 | 12_53752692_G_A_b37 | 757369 | 100 | 0.152 | 0.463 | -0.045 | 0.062 |
| rs11170516 | ENSG00000170486.6 | KRT72 | 12_53752692_G_A_b37 | 757442 | 100 | 0.152 | 0.903 | 0.010 | 0.082 |
| rs11170516 | ENSG00000186081.7 | KRT5 | 12_53752692_G_A_b37 | 838221 | 100 | 0.152 | 0.689 | 0.030 | 0.074 |
| rs11170516 | ENSG00000205420.6 | KRT6A | 12_53752692_G_A_b37 | 865651 | 100 | 0.152 | 0.533 | 0.042 | 0.068 |
| rs11170516 | ENSG00000170465.9 | KRT6C | 12_53752692_G_A_b37 | 885123 | 100 | 0.152 | 0.652 | 0.034 | 0.075 |
| rs11170516 | ENSG00000185479.5 | KRT6B | 12_53752692_G_A_b37 | 906782 | 100 | 0.152 | 0.986 | 0.002 | 0.083 |
| rs35437931 | ENSG00000258086.1 | RP11-753H16.5 | 12_53756354_T_C_b37 | -991222 | 106 | 0.160 | 0.008 | -0.188 | 0.070 |
| rs35437931 | ENSG00000111481.5 | COPZ1 | 12_53756354_T_C_b37 | -938632 | 106 | 0.160 | 0.974 | -0.001 | 0.030 |
| rs35437931 | ENSG00000123405.9 | NFE2 | 12_53756354_T_C_b37 | -938551 | 106 | 0.160 | 0.500 | -0.015 | 0.022 |
| rs35437931 | ENSG00000135486.13 | HNRNPA1 | 12_53756354_T_C_b37 | -917623 | 106 | 0.160 | 0.775 | 0.010 | 0.036 |
| rs35437931 | ENSG00000094916.9 | CBX5 | 12_53756354_T_C_b37 | -917532 | 106 | 0.160 | 0.143 | 0.059 | 0.040 |
| rs35437931 | ENSG00000257596.1 | RP11-968A15.2 | 12_53756354_T_C_b37 | -900045 | 106 | 0.160 | 0.988 | 0.001 | 0.069 |
| rs35437931 | ENSG00000123415.10 | SMUG1 | 12_53756354_T_C_b37 | -826424 | 106 | 0.160 | 0.143 | -0.047 | 0.032 |
| rs35437931 | ENSG00000250742.1 | RP11-834C11.4 | 12_53756354_T_C_b37 | -763528 | 106 | 0.160 | 0.571 | 0.050 | 0.088 |
| rs35437931 | ENSG00000250654.3 | RP11-834C11.7 | 12_53756354_T_C_b37 | -719333 | 106 | 0.160 | 0.349 | 0.075 | 0.080 |
| rs35437931 | ENSG00000172789.3 | HOXC5 | 12_53756354_T_C_b37 | -670283 | 106 | 0.160 | 0.267 | 0.084 | 0.075 |
| rs35437931 | ENSG00000180818.4 | HOXC10 | 12_53756354_T_C_b37 | -622514 | 106 | 0.160 | 0.459 | -0.052 | 0.070 |
| rs35437931 | ENSG00000012822.11 | CALCOCO1 | 12_53756354_T_C_b37 | -365175 | 106 | 0.160 | 0.079 | -0.064 | 0.036 |
| rs35437931 | ENSG00000135390.13 | ATP5G2 | 12_53756354_T_C_b37 | -314838 | 106 | 0.160 | 0.814 | -0.005 | 0.023 |
| rs35437931 | ENSG00000170653.14 | ATF7 | 12_53756354_T_C_b37 | -263845 | 106 | 0.160 | 0.419 | -0.027 | 0.034 |
| rs35437931 | ENSG00000139574.7 | NPFF | 12_53756354_T_C_b37 | -145068 | 106 | 0.160 | 0.102 | -0.050 | 0.031 |
| rs35437931 | ENSG00000139546.6 | TARBP2 | 12_53756354_T_C_b37 | -138367 | 106 | 0.160 | 0.003 | -0.101 | 0.034 |
| rs35437931 | ENSG00000270175.1 | RP11-793H13.11 | 12_53756354_T_C_b37 | -138350 | 106 | 0.160 | 0.031 | -0.173 | 0.080 |
| rs35437931 | ENSG00000139625.8 | MAP3K12 | 12_53756354_T_C_b37 | -137493 | 106 | 0.160 | 0.090 | -0.068 | 0.040 |
| rs35437931 | ENSG00000197111.11 | PCBP2 | 12_53756354_T_C_b37 | -89532 | 106 | 0.160 | 0.153 | -0.045 | 0.031 |
| rs35437931 | ENSG00000205352.6 | PRR13 | 12_53756354_T_C_b37 | -79035 | 106 | 0.160 | 0.021 | 0.039 | 0.017 |
| rs35437931 | ENSG00000135409.6 | AMHR2 | 12_53756354_T_C_b37 | -61285 | 106 | 0.160 | 0.818 | 0.021 | 0.089 |
| rs35437931 | ENSG00000185591.5 | SP1 | 12_53756354_T_C_b37 | -17606 | 106 | 0.160 | 1.64393E-08 | 0.120 | 0.021 |
| rs35437931 | ENSG00000094914.8 | AAAS | 12_53756354_T_C_b37 | 37706 | 106 | 0.160 | 0.912 | -0.004 | 0.032 |
| rs35437931 | ENSG00000257605.1 | RP11-680A11.5 | 12_53756354_T_C_b37 | 62256 | 106 | 0.160 | 0.011 | 0.170 | 0.066 |
| rs35437931 | ENSG00000139637.9 | C12orf10 | 12_53756354_T_C_b37 | 62884 | 106 | 0.160 | 0.373 | -0.032 | 0.036 |
| rs35437931 | ENSG00000123349.9 | PFDN5 | 12_53756354_T_C_b37 | 67279 | 106 | 0.160 | 0.480 | -0.018 | 0.025 |
| rs35437931 | ENSG00000135476.7 | ESPL1 | 12_53756354_T_C_b37 | 94271 | 106 | 0.160 | 0.240 | -0.040 | 0.034 |
| rs35437931 | ENSG00000182544.8 | MFSD5 | 12_53756354_T_C_b37 | 111319 | 106 | 0.160 | 0.097 | -0.045 | 0.027 |
| rs35437931 | ENSG00000172819.12 | RARG | 12_53756354_T_C_b37 | 129590 | 106 | 0.160 | 0.199 | 0.055 | 0.043 |
| rs35437931 | ENSG00000139626.11 | ITGB7 | 12_53756354_T_C_b37 | 155263 | 106 | 0.160 | 0.599 | 0.013 | 0.024 |
| rs35437931 | ENSG00000139631.14 | CSAD | 12_53756354_T_C_b37 | 181219 | 106 | 0.160 | 0.174 | 0.042 | 0.031 |
| rs35437931 | ENSG00000139651.9 | ZNF740 | 12_53756354_T_C_b37 | 181870 | 106 | 0.160 | 0.596 | 0.018 | 0.033 |
| rs35437931 | ENSG00000257790.1 | RP11-1136G11.6 | 12_53756354_T_C_b37 | 209298 | 106 | 0.160 | 0.034 | -0.166 | 0.078 |
| rs35437931 | ENSG00000167780.7 | SOAT2 | 12_53756354_T_C_b37 | 259052 | 106 | 0.160 | 0.363 | -0.063 | 0.069 |
| rs35437931 | ENSG00000167779.3 | IGFBP6 | 12_53756354_T_C_b37 | 265134 | 106 | 0.160 | 0.165 | 0.074 | 0.053 |
| rs35437931 | ENSG00000167778.4 | SPRYD3 | 12_53756354_T_C_b37 | 283150 | 106 | 0.160 | 0.593 | 0.013 | 0.025 |
| rs35437931 | ENSG00000257337.2 | RP11-983P16.4 | 12_53756354_T_C_b37 | 308996 | 106 | 0.160 | 0.685 | 0.020 | 0.049 |
| rs35437931 | ENSG00000111077.13 | TENC1 | 12_53756354_T_C_b37 | 315519 | 106 | 0.160 | 0.433 | 0.031 | 0.039 |
| rs35437931 | ENSG00000063046.13 | EIF4B | 12_53756354_T_C_b37 | 356412 | 106 | 0.160 | 0.614 | -0.016 | 0.031 |
| rs35437931 | ENSG00000170421.7 | KRT8 | 12_53756354_T_C_b37 | 412650 | 106 | 0.160 | 0.922 | -0.006 | 0.061 |
| rs35437931 | ENSG00000111057.6 | KRT18 | 12_53756354_T_C_b37 | 413699 | 106 | 0.160 | 0.136 | 0.082 | 0.055 |
| rs35437931 | ENSG00000170423.8 | KRT78 | 12_53756354_T_C_b37 | 513478 | 106 | 0.160 | 0.868 | -0.014 | 0.083 |
| rs35437931 | ENSG00000185640.5 | KRT79 | 12_53756354_T_C_b37 | 528275 | 106 | 0.160 | 0.385 | -0.075 | 0.086 |
| rs35437931 | ENSG00000170477.8 | KRT4 | 12_53756354_T_C_b37 | 548019 | 106 | 0.160 | 0.249 | 0.075 | 0.065 |
| rs35437931 | ENSG00000189182.5 | KRT77 | 12_53756354_T_C_b37 | 659107 | 106 | 0.160 | 0.650 | 0.043 | 0.095 |
| rs35437931 | ENSG00000167768.4 | KRT1 | 12_53756354_T_C_b37 | 682163 | 106 | 0.160 | 0.310 | 0.053 | 0.053 |
| rs35437931 | ENSG00000172867.3 | KRT2 | 12_53756354_T_C_b37 | 710406 | 106 | 0.160 | 0.708 | 0.030 | 0.081 |
| rs35437931 | ENSG00000186049.4 | KRT73 | 12_53756354_T_C_b37 | 744011 | 106 | 0.160 | 0.984 | 0.001 | 0.071 |
| rs35437931 | ENSG00000257495.1 | RP11-641A6.2 | 12_53756354_T_C_b37 | 761031 | 106 | 0.160 | 0.250 | -0.070 | 0.061 |
| rs35437931 | ENSG00000170486.6 | KRT72 | 12_53756354_T_C_b37 | 761104 | 106 | 0.160 | 0.972 | -0.003 | 0.081 |
| rs35437931 | ENSG00000186081.7 | KRT5 | 12_53756354_T_C_b37 | 841883 | 106 | 0.160 | 0.792 | 0.019 | 0.074 |
| rs35437931 | ENSG00000205420.6 | KRT6A | 12_53756354_T_C_b37 | 869313 | 106 | 0.160 | 0.559 | 0.039 | 0.067 |
| rs35437931 | ENSG00000170465.9 | KRT6C | 12_53756354_T_C_b37 | 888785 | 106 | 0.160 | 0.742 | 0.025 | 0.074 |
| rs35437931 | ENSG00000185479.5 | KRT6B | 12_53756354_T_C_b37 | 910444 | 106 | 0.160 | 0.816 | -0.019 | 0.082 |
| rs12828860 | ENSG00000139572.3 | GPR84 | 12_53759803_G_C_b37 | -998468 | 106 | 0.160 | 0.493 | 0.022 | 0.032 |
| rs12828860 | ENSG00000258086.1 | RP11-753H16.5 | 12_53759803_G_C_b37 | -987773 | 106 | 0.160 | 0.008 | -0.188 | 0.070 |
| rs12828860 | ENSG00000111481.5 | COPZ1 | 12_53759803_G_C_b37 | -935183 | 106 | 0.160 | 0.974 | -0.001 | 0.030 |
| rs12828860 | ENSG00000123405.9 | NFE2 | 12_53759803_G_C_b37 | -935102 | 106 | 0.160 | 0.500 | -0.015 | 0.022 |
| rs12828860 | ENSG00000135486.13 | HNRNPA1 | 12_53759803_G_C_b37 | -914174 | 106 | 0.160 | 0.775 | 0.010 | 0.036 |
| rs12828860 | ENSG00000094916.9 | CBX5 | 12_53759803_G_C_b37 | -914083 | 106 | 0.160 | 0.143 | 0.059 | 0.040 |
| rs12828860 | ENSG00000257596.1 | RP11-968A15.2 | 12_53759803_G_C_b37 | -896596 | 106 | 0.160 | 0.988 | 0.001 | 0.069 |
| rs12828860 | ENSG00000123415.10 | SMUG1 | 12_53759803_G_C_b37 | -822975 | 106 | 0.160 | 0.143 | -0.047 | 0.032 |
| rs12828860 | ENSG00000250742.1 | RP11-834C11.4 | 12_53759803_G_C_b37 | -760079 | 106 | 0.160 | 0.571 | 0.050 | 0.088 |
| rs12828860 | ENSG00000250654.3 | RP11-834C11.7 | 12_53759803_G_C_b37 | -715884 | 106 | 0.160 | 0.349 | 0.075 | 0.080 |
| rs12828860 | ENSG00000172789.3 | HOXC5 | 12_53759803_G_C_b37 | -666834 | 106 | 0.160 | 0.267 | 0.084 | 0.075 |
| rs12828860 | ENSG00000180818.4 | HOXC10 | 12_53759803_G_C_b37 | -619065 | 106 | 0.160 | 0.459 | -0.052 | 0.070 |
| rs12828860 | ENSG00000012822.11 | CALCOCO1 | 12_53759803_G_C_b37 | -361726 | 106 | 0.160 | 0.079 | -0.064 | 0.036 |
| rs12828860 | ENSG00000135390.13 | ATP5G2 | 12_53759803_G_C_b37 | -311389 | 106 | 0.160 | 0.814 | -0.005 | 0.023 |
| rs12828860 | ENSG00000170653.14 | ATF7 | 12_53759803_G_C_b37 | -260396 | 106 | 0.160 | 0.419 | -0.027 | 0.034 |
| rs12828860 | ENSG00000139574.7 | NPFF | 12_53759803_G_C_b37 | -141619 | 106 | 0.160 | 0.102 | -0.050 | 0.031 |
| rs12828860 | ENSG00000139546.6 | TARBP2 | 12_53759803_G_C_b37 | -134918 | 106 | 0.160 | 0.003 | -0.101 | 0.034 |
| rs12828860 | ENSG00000270175.1 | RP11-793H13.11 | 12_53759803_G_C_b37 | -134901 | 106 | 0.160 | 0.031 | -0.173 | 0.080 |
| rs12828860 | ENSG00000139625.8 | MAP3K12 | 12_53759803_G_C_b37 | -134044 | 106 | 0.160 | 0.090 | -0.068 | 0.040 |
| rs12828860 | ENSG00000197111.11 | PCBP2 | 12_53759803_G_C_b37 | -86083 | 106 | 0.160 | 0.153 | -0.045 | 0.031 |
| rs12828860 | ENSG00000205352.6 | PRR13 | 12_53759803_G_C_b37 | -75586 | 106 | 0.160 | 0.021 | 0.039 | 0.017 |
| rs12828860 | ENSG00000135409.6 | AMHR2 | 12_53759803_G_C_b37 | -57836 | 106 | 0.160 | 0.818 | 0.021 | 0.089 |
| rs12828860 | ENSG00000185591.5 | SP1 | 12_53759803_G_C_b37 | -14157 | 106 | 0.160 | 1.64393E-08 | 0.120 | 0.021 |
| rs12828860 | ENSG00000094914.8 | AAAS | 12_53759803_G_C_b37 | 41155 | 106 | 0.160 | 0.912 | -0.004 | 0.032 |
| rs12828860 | ENSG00000257605.1 | RP11-680A11.5 | 12_53759803_G_C_b37 | 65705 | 106 | 0.160 | 0.011 | 0.170 | 0.066 |
| rs12828860 | ENSG00000139637.9 | C12orf10 | 12_53759803_G_C_b37 | 66333 | 106 | 0.160 | 0.373 | -0.032 | 0.036 |
| rs12828860 | ENSG00000123349.9 | PFDN5 | 12_53759803_G_C_b37 | 70728 | 106 | 0.160 | 0.480 | -0.018 | 0.025 |
| rs12828860 | ENSG00000135476.7 | ESPL1 | 12_53759803_G_C_b37 | 97720 | 106 | 0.160 | 0.240 | -0.040 | 0.034 |
| rs12828860 | ENSG00000182544.8 | MFSD5 | 12_53759803_G_C_b37 | 114768 | 106 | 0.160 | 0.097 | -0.045 | 0.027 |
| rs12828860 | ENSG00000172819.12 | RARG | 12_53759803_G_C_b37 | 133039 | 106 | 0.160 | 0.199 | 0.055 | 0.043 |
| rs12828860 | ENSG00000139626.11 | ITGB7 | 12_53759803_G_C_b37 | 158712 | 106 | 0.160 | 0.599 | 0.013 | 0.024 |
| rs12828860 | ENSG00000139631.14 | CSAD | 12_53759803_G_C_b37 | 184668 | 106 | 0.160 | 0.174 | 0.042 | 0.031 |
| rs12828860 | ENSG00000139651.9 | ZNF740 | 12_53759803_G_C_b37 | 185319 | 106 | 0.160 | 0.596 | 0.018 | 0.033 |
| rs12828860 | ENSG00000257790.1 | RP11-1136G11.6 | 12_53759803_G_C_b37 | 212747 | 106 | 0.160 | 0.034 | -0.166 | 0.078 |
| rs12828860 | ENSG00000167780.7 | SOAT2 | 12_53759803_G_C_b37 | 262501 | 106 | 0.160 | 0.363 | -0.063 | 0.069 |
| rs12828860 | ENSG00000167779.3 | IGFBP6 | 12_53759803_G_C_b37 | 268583 | 106 | 0.160 | 0.165 | 0.074 | 0.053 |
| rs12828860 | ENSG00000167778.4 | SPRYD3 | 12_53759803_G_C_b37 | 286599 | 106 | 0.160 | 0.593 | 0.013 | 0.025 |
| rs12828860 | ENSG00000257337.2 | RP11-983P16.4 | 12_53759803_G_C_b37 | 312445 | 106 | 0.160 | 0.685 | 0.020 | 0.049 |
| rs12828860 | ENSG00000111077.13 | TENC1 | 12_53759803_G_C_b37 | 318968 | 106 | 0.160 | 0.433 | 0.031 | 0.039 |
| rs12828860 | ENSG00000063046.13 | EIF4B | 12_53759803_G_C_b37 | 359861 | 106 | 0.160 | 0.614 | -0.016 | 0.031 |
| rs12828860 | ENSG00000170421.7 | KRT8 | 12_53759803_G_C_b37 | 416099 | 106 | 0.160 | 0.922 | -0.006 | 0.061 |
| rs12828860 | ENSG00000111057.6 | KRT18 | 12_53759803_G_C_b37 | 417148 | 106 | 0.160 | 0.136 | 0.082 | 0.055 |
| rs12828860 | ENSG00000170423.8 | KRT78 | 12_53759803_G_C_b37 | 516927 | 106 | 0.160 | 0.868 | -0.014 | 0.083 |
| rs12828860 | ENSG00000185640.5 | KRT79 | 12_53759803_G_C_b37 | 531724 | 106 | 0.160 | 0.385 | -0.075 | 0.086 |
| rs12828860 | ENSG00000170477.8 | KRT4 | 12_53759803_G_C_b37 | 551468 | 106 | 0.160 | 0.249 | 0.075 | 0.065 |
| rs12828860 | ENSG00000189182.5 | KRT77 | 12_53759803_G_C_b37 | 662556 | 106 | 0.160 | 0.650 | 0.043 | 0.095 |
| rs12828860 | ENSG00000167768.4 | KRT1 | 12_53759803_G_C_b37 | 685612 | 106 | 0.160 | 0.310 | 0.053 | 0.053 |
| rs12828860 | ENSG00000172867.3 | KRT2 | 12_53759803_G_C_b37 | 713855 | 106 | 0.160 | 0.708 | 0.030 | 0.081 |
| rs12828860 | ENSG00000186049.4 | KRT73 | 12_53759803_G_C_b37 | 747460 | 106 | 0.160 | 0.984 | 0.001 | 0.071 |
| rs12828860 | ENSG00000257495.1 | RP11-641A6.2 | 12_53759803_G_C_b37 | 764480 | 106 | 0.160 | 0.250 | -0.070 | 0.061 |
| rs12828860 | ENSG00000170486.6 | KRT72 | 12_53759803_G_C_b37 | 764553 | 106 | 0.160 | 0.972 | -0.003 | 0.081 |
| rs12828860 | ENSG00000186081.7 | KRT5 | 12_53759803_G_C_b37 | 845332 | 106 | 0.160 | 0.792 | 0.019 | 0.074 |
| rs12828860 | ENSG00000205420.6 | KRT6A | 12_53759803_G_C_b37 | 872762 | 106 | 0.160 | 0.559 | 0.039 | 0.067 |
| rs12828860 | ENSG00000170465.9 | KRT6C | 12_53759803_G_C_b37 | 892234 | 106 | 0.160 | 0.742 | 0.025 | 0.074 |
| rs12828860 | ENSG00000185479.5 | KRT6B | 12_53759803_G_C_b37 | 913893 | 106 | 0.160 | 0.816 | -0.019 | 0.082 |
| rs58123204 | ENSG00000139572.3 | GPR84 | 12_53760162_A_G_b37 | -998109 | 104 | 0.157 | 0.536 | 0.020 | 0.032 |
| rs58123204 | ENSG00000258086.1 | RP11-753H16.5 | 12_53760162_A_G_b37 | -987414 | 104 | 0.157 | 0.009 | -0.186 | 0.071 |
| rs58123204 | ENSG00000111481.5 | COPZ1 | 12_53760162_A_G_b37 | -934824 | 104 | 0.157 | 0.918 | -0.003 | 0.030 |
| rs58123204 | ENSG00000123405.9 | NFE2 | 12_53760162_A_G_b37 | -934743 | 104 | 0.157 | 0.572 | -0.013 | 0.022 |
| rs58123204 | ENSG00000135486.13 | HNRNPA1 | 12_53760162_A_G_b37 | -913815 | 104 | 0.157 | 0.715 | 0.013 | 0.036 |
| rs58123204 | ENSG00000094916.9 | CBX5 | 12_53760162_A_G_b37 | -913724 | 104 | 0.157 | 0.166 | 0.056 | 0.040 |
| rs58123204 | ENSG00000257596.1 | RP11-968A15.2 | 12_53760162_A_G_b37 | -896237 | 104 | 0.157 | 0.942 | -0.005 | 0.069 |
| rs58123204 | ENSG00000123415.10 | SMUG1 | 12_53760162_A_G_b37 | -822616 | 104 | 0.157 | 0.191 | -0.043 | 0.032 |
| rs58123204 | ENSG00000250742.1 | RP11-834C11.4 | 12_53760162_A_G_b37 | -759720 | 104 | 0.157 | 0.667 | 0.038 | 0.088 |
| rs58123204 | ENSG00000250654.3 | RP11-834C11.7 | 12_53760162_A_G_b37 | -715525 | 104 | 0.157 | 0.285 | 0.086 | 0.081 |
| rs58123204 | ENSG00000172789.3 | HOXC5 | 12_53760162_A_G_b37 | -666475 | 104 | 0.157 | 0.265 | 0.084 | 0.075 |
| rs58123204 | ENSG00000180818.4 | HOXC10 | 12_53760162_A_G_b37 | -618706 | 104 | 0.157 | 0.323 | -0.069 | 0.070 |
| rs58123204 | ENSG00000012822.11 | CALCOCO1 | 12_53760162_A_G_b37 | -361367 | 104 | 0.157 | 0.078 | -0.064 | 0.036 |
| rs58123204 | ENSG00000135390.13 | ATP5G2 | 12_53760162_A_G_b37 | -311030 | 104 | 0.157 | 0.950 | -0.001 | 0.023 |
| rs58123204 | ENSG00000170653.14 | ATF7 | 12_53760162_A_G_b37 | -260037 | 104 | 0.157 | 0.340 | -0.032 | 0.034 |
| rs58123204 | ENSG00000139574.7 | NPFF | 12_53760162_A_G_b37 | -141260 | 104 | 0.157 | 0.110 | -0.049 | 0.031 |
| rs58123204 | ENSG00000139546.6 | TARBP2 | 12_53760162_A_G_b37 | -134559 | 104 | 0.157 | 0.005 | -0.098 | 0.035 |
| rs58123204 | ENSG00000270175.1 | RP11-793H13.11 | 12_53760162_A_G_b37 | -134542 | 104 | 0.157 | 0.031 | -0.174 | 0.080 |
| rs58123204 | ENSG00000139625.8 | MAP3K12 | 12_53760162_A_G_b37 | -133685 | 104 | 0.157 | 0.101 | -0.066 | 0.040 |
| rs58123204 | ENSG00000197111.11 | PCBP2 | 12_53760162_A_G_b37 | -85724 | 104 | 0.157 | 0.150 | -0.045 | 0.031 |
| rs58123204 | ENSG00000205352.6 | PRR13 | 12_53760162_A_G_b37 | -75227 | 104 | 0.157 | 0.015 | 0.041 | 0.017 |
| rs58123204 | ENSG00000135409.6 | AMHR2 | 12_53760162_A_G_b37 | -57477 | 104 | 0.157 | 0.870 | 0.015 | 0.090 |
| rs58123204 | ENSG00000185591.5 | SP1 | 12_53760162_A_G_b37 | -13798 | 104 | 0.157 | 3.56958E-08 | 0.118 | 0.021 |
| rs58123204 | ENSG00000094914.8 | AAAS | 12_53760162_A_G_b37 | 41514 | 104 | 0.157 | 0.886 | -0.005 | 0.032 |
| rs58123204 | ENSG00000257605.1 | RP11-680A11.5 | 12_53760162_A_G_b37 | 66064 | 104 | 0.157 | 0.027 | 0.149 | 0.067 |
| rs58123204 | ENSG00000139637.9 | C12orf10 | 12_53760162_A_G_b37 | 66692 | 104 | 0.157 | 0.448 | -0.027 | 0.036 |
| rs58123204 | ENSG00000123349.9 | PFDN5 | 12_53760162_A_G_b37 | 71087 | 104 | 0.157 | 0.458 | -0.019 | 0.025 |
| rs58123204 | ENSG00000135476.7 | ESPL1 | 12_53760162_A_G_b37 | 98079 | 104 | 0.157 | 0.281 | -0.037 | 0.035 |
| rs58123204 | ENSG00000182544.8 | MFSD5 | 12_53760162_A_G_b37 | 115127 | 104 | 0.157 | 0.081 | -0.047 | 0.027 |
| rs58123204 | ENSG00000172819.12 | RARG | 12_53760162_A_G_b37 | 133398 | 104 | 0.157 | 0.277 | 0.047 | 0.043 |
| rs58123204 | ENSG00000139626.11 | ITGB7 | 12_53760162_A_G_b37 | 159071 | 104 | 0.157 | 0.603 | 0.013 | 0.025 |
| rs58123204 | ENSG00000139631.14 | CSAD | 12_53760162_A_G_b37 | 185027 | 104 | 0.157 | 0.207 | 0.039 | 0.031 |
| rs58123204 | ENSG00000139651.9 | ZNF740 | 12_53760162_A_G_b37 | 185678 | 104 | 0.157 | 0.750 | 0.011 | 0.033 |
| rs58123204 | ENSG00000257790.1 | RP11-1136G11.6 | 12_53760162_A_G_b37 | 213106 | 104 | 0.157 | 0.032 | -0.169 | 0.078 |
| rs58123204 | ENSG00000167780.7 | SOAT2 | 12_53760162_A_G_b37 | 262860 | 104 | 0.157 | 0.382 | -0.061 | 0.069 |
| rs58123204 | ENSG00000167779.3 | IGFBP6 | 12_53760162_A_G_b37 | 268942 | 104 | 0.157 | 0.189 | 0.070 | 0.053 |
| rs58123204 | ENSG00000167778.4 | SPRYD3 | 12_53760162_A_G_b37 | 286958 | 104 | 0.157 | 0.662 | 0.011 | 0.025 |
| rs58123204 | ENSG00000257337.2 | RP11-983P16.4 | 12_53760162_A_G_b37 | 312804 | 104 | 0.157 | 0.638 | 0.023 | 0.049 |
| rs58123204 | ENSG00000111077.13 | TENC1 | 12_53760162_A_G_b37 | 319327 | 104 | 0.157 | 0.298 | 0.041 | 0.039 |
| rs58123204 | ENSG00000063046.13 | EIF4B | 12_53760162_A_G_b37 | 360220 | 104 | 0.157 | 0.524 | -0.020 | 0.031 |
| rs58123204 | ENSG00000170421.7 | KRT8 | 12_53760162_A_G_b37 | 416458 | 104 | 0.157 | 0.885 | -0.009 | 0.061 |
| rs58123204 | ENSG00000111057.6 | KRT18 | 12_53760162_A_G_b37 | 417507 | 104 | 0.157 | 0.129 | 0.084 | 0.055 |
| rs58123204 | ENSG00000170423.8 | KRT78 | 12_53760162_A_G_b37 | 517286 | 104 | 0.157 | 0.962 | -0.004 | 0.083 |
| rs58123204 | ENSG00000185640.5 | KRT79 | 12_53760162_A_G_b37 | 532083 | 104 | 0.157 | 0.313 | -0.087 | 0.086 |
| rs58123204 | ENSG00000170477.8 | KRT4 | 12_53760162_A_G_b37 | 551827 | 104 | 0.157 | 0.268 | 0.072 | 0.065 |
| rs58123204 | ENSG00000189182.5 | KRT77 | 12_53760162_A_G_b37 | 662915 | 104 | 0.157 | 0.497 | 0.065 | 0.096 |
| rs58123204 | ENSG00000167768.4 | KRT1 | 12_53760162_A_G_b37 | 685971 | 104 | 0.157 | 0.414 | 0.043 | 0.053 |
| rs58123204 | ENSG00000172867.3 | KRT2 | 12_53760162_A_G_b37 | 714214 | 104 | 0.157 | 0.626 | 0.040 | 0.081 |
| rs58123204 | ENSG00000186049.4 | KRT73 | 12_53760162_A_G_b37 | 747819 | 104 | 0.157 | 0.994 | 0.001 | 0.071 |
| rs58123204 | ENSG00000257495.1 | RP11-641A6.2 | 12_53760162_A_G_b37 | 764839 | 104 | 0.157 | 0.240 | -0.072 | 0.061 |
| rs58123204 | ENSG00000170486.6 | KRT72 | 12_53760162_A_G_b37 | 764912 | 104 | 0.157 | 0.865 | -0.014 | 0.081 |
| rs58123204 | ENSG00000186081.7 | KRT5 | 12_53760162_A_G_b37 | 845691 | 104 | 0.157 | 0.814 | 0.017 | 0.074 |
| rs58123204 | ENSG00000205420.6 | KRT6A | 12_53760162_A_G_b37 | 873121 | 104 | 0.157 | 0.558 | 0.040 | 0.067 |
| rs58123204 | ENSG00000170465.9 | KRT6C | 12_53760162_A_G_b37 | 892593 | 104 | 0.157 | 0.701 | 0.029 | 0.075 |
| rs58123204 | ENSG00000185479.5 | KRT6B | 12_53760162_A_G_b37 | 914252 | 104 | 0.157 | 0.810 | -0.020 | 0.083 |
| rs7962345 | ENSG00000139572.3 | GPR84 | 12_53762887_C_T_b37 | -995384 | 131 | 0.222 | 0.510 | -0.020 | 0.030 |
| rs7962345 | ENSG00000258086.1 | RP11-753H16.5 | 12_53762887_C_T_b37 | -984689 | 131 | 0.222 | 0.001 | 0.215 | 0.065 |
| rs7962345 | ENSG00000111481.5 | COPZ1 | 12_53762887_C_T_b37 | -932099 | 131 | 0.222 | 0.370 | -0.025 | 0.028 |
| rs7962345 | ENSG00000123405.9 | NFE2 | 12_53762887_C_T_b37 | -932018 | 131 | 0.222 | 0.594 | 0.011 | 0.021 |
| rs7962345 | ENSG00000135486.13 | HNRNPA1 | 12_53762887_C_T_b37 | -911090 | 131 | 0.222 | 0.606 | -0.017 | 0.034 |
| rs7962345 | ENSG00000094916.9 | CBX5 | 12_53762887_C_T_b37 | -910999 | 131 | 0.222 | 0.068 | -0.069 | 0.038 |
| rs7962345 | ENSG00000257596.1 | RP11-968A15.2 | 12_53762887_C_T_b37 | -893512 | 131 | 0.222 | 0.837 | 0.013 | 0.064 |
| rs7962345 | ENSG00000123415.10 | SMUG1 | 12_53762887_C_T_b37 | -819891 | 131 | 0.222 | 0.320 | 0.030 | 0.030 |
| rs7962345 | ENSG00000250742.1 | RP11-834C11.4 | 12_53762887_C_T_b37 | -756995 | 131 | 0.222 | 0.601 | 0.043 | 0.082 |
| rs7962345 | ENSG00000250654.3 | RP11-834C11.7 | 12_53762887_C_T_b37 | -712800 | 131 | 0.222 | 0.249 | -0.087 | 0.075 |
| rs7962345 | ENSG00000172789.3 | HOXC5 | 12_53762887_C_T_b37 | -663750 | 131 | 0.222 | 0.372 | -0.063 | 0.070 |
| rs7962345 | ENSG00000180818.4 | HOXC10 | 12_53762887_C_T_b37 | -615981 | 131 | 0.222 | 0.667 | 0.028 | 0.065 |
| rs7962345 | ENSG00000012822.11 | CALCOCO1 | 12_53762887_C_T_b37 | -358642 | 131 | 0.222 | 0.099 | 0.056 | 0.034 |
| rs7962345 | ENSG00000135390.13 | ATP5G2 | 12_53762887_C_T_b37 | -308305 | 131 | 0.222 | 0.340 | 0.020 | 0.021 |
| rs7962345 | ENSG00000170653.14 | ATF7 | 12_53762887_C_T_b37 | -257312 | 131 | 0.222 | 0.109 | 0.050 | 0.031 |
| rs7962345 | ENSG00000139574.7 | NPFF | 12_53762887_C_T_b37 | -138535 | 131 | 0.222 | 0.055 | 0.055 | 0.029 |
| rs7962345 | ENSG00000139546.6 | TARBP2 | 12_53762887_C_T_b37 | -131834 | 131 | 0.222 | 0.022 | 0.074 | 0.032 |
| rs7962345 | ENSG00000270175.1 | RP11-793H13.11 | 12_53762887_C_T_b37 | -131817 | 131 | 0.222 | 0.112 | 0.119 | 0.075 |
| rs7962345 | ENSG00000139625.8 | MAP3K12 | 12_53762887_C_T_b37 | -130960 | 131 | 0.222 | 0.088 | 0.064 | 0.037 |
| rs7962345 | ENSG00000197111.11 | PCBP2 | 12_53762887_C_T_b37 | -82999 | 131 | 0.222 | 0.245 | 0.034 | 0.029 |
| rs7962345 | ENSG00000205352.6 | PRR13 | 12_53762887_C_T_b37 | -72502 | 131 | 0.222 | 0.231 | -0.019 | 0.016 |
| rs7962345 | ENSG00000135409.6 | AMHR2 | 12_53762887_C_T_b37 | -54752 | 131 | 0.222 | 0.869 | 0.014 | 0.083 |
| rs7962345 | ENSG00000185591.5 | SP1 | 12_53762887_C_T_b37 | -11073 | 131 | 0.222 | 4.41384E-07 | -0.101 | 0.019 |
| rs7962345 | ENSG00000094914.8 | AAAS | 12_53762887_C_T_b37 | 44239 | 131 | 0.222 | 0.981 | -0.001 | 0.030 |
| rs7962345 | ENSG00000257605.1 | RP11-680A11.5 | 12_53762887_C_T_b37 | 68789 | 131 | 0.222 | 0.008 | -0.166 | 0.062 |
| rs7962345 | ENSG00000139637.9 | C12orf10 | 12_53762887_C_T_b37 | 69417 | 131 | 0.222 | 0.636 | 0.016 | 0.034 |
| rs7962345 | ENSG00000123349.9 | PFDN5 | 12_53762887_C_T_b37 | 73812 | 131 | 0.222 | 0.951 | 0.001 | 0.024 |
| rs7962345 | ENSG00000135476.7 | ESPL1 | 12_53762887_C_T_b37 | 100804 | 131 | 0.222 | 0.117 | 0.050 | 0.032 |
| rs7962345 | ENSG00000182544.8 | MFSD5 | 12_53762887_C_T_b37 | 117852 | 131 | 0.222 | 0.184 | 0.033 | 0.025 |
| rs7962345 | ENSG00000172819.12 | RARG | 12_53762887_C_T_b37 | 136123 | 131 | 0.222 | 0.221 | -0.049 | 0.040 |
| rs7962345 | ENSG00000139626.11 | ITGB7 | 12_53762887_C_T_b37 | 161796 | 131 | 0.222 | 0.200 | -0.029 | 0.023 |
| rs7962345 | ENSG00000139631.14 | CSAD | 12_53762887_C_T_b37 | 187752 | 131 | 0.222 | 0.150 | -0.041 | 0.029 |
| rs7962345 | ENSG00000139651.9 | ZNF740 | 12_53762887_C_T_b37 | 188403 | 131 | 0.222 | 0.566 | -0.018 | 0.031 |
| rs7962345 | ENSG00000257790.1 | RP11-1136G11.6 | 12_53762887_C_T_b37 | 215831 | 131 | 0.222 | 0.066 | 0.135 | 0.073 |
| rs7962345 | ENSG00000167780.7 | SOAT2 | 12_53762887_C_T_b37 | 265585 | 131 | 0.222 | 0.162 | 0.090 | 0.064 |
| rs7962345 | ENSG00000167779.3 | IGFBP6 | 12_53762887_C_T_b37 | 271667 | 131 | 0.222 | 0.710 | -0.018 | 0.050 |
| rs7962345 | ENSG00000167778.4 | SPRYD3 | 12_53762887_C_T_b37 | 289683 | 131 | 0.222 | 0.408 | -0.019 | 0.023 |
| rs7962345 | ENSG00000257337.2 | RP11-983P16.4 | 12_53762887_C_T_b37 | 315529 | 131 | 0.222 | 0.793 | -0.012 | 0.046 |
| rs7962345 | ENSG00000111077.13 | TENC1 | 12_53762887_C_T_b37 | 322052 | 131 | 0.222 | 0.219 | -0.045 | 0.037 |
| rs7962345 | ENSG00000063046.13 | EIF4B | 12_53762887_C_T_b37 | 362945 | 131 | 0.222 | 0.741 | 0.010 | 0.029 |
| rs7962345 | ENSG00000170421.7 | KRT8 | 12_53762887_C_T_b37 | 419183 | 131 | 0.222 | 0.926 | 0.005 | 0.057 |
| rs7962345 | ENSG00000111057.6 | KRT18 | 12_53762887_C_T_b37 | 420232 | 131 | 0.222 | 0.104 | -0.084 | 0.052 |
| rs7962345 | ENSG00000170423.8 | KRT78 | 12_53762887_C_T_b37 | 520011 | 131 | 0.222 | 0.232 | 0.093 | 0.077 |
| rs7962345 | ENSG00000185640.5 | KRT79 | 12_53762887_C_T_b37 | 534808 | 131 | 0.222 | 0.214 | 0.100 | 0.080 |
| rs7962345 | ENSG00000170477.8 | KRT4 | 12_53762887_C_T_b37 | 554552 | 131 | 0.222 | 0.648 | -0.028 | 0.061 |
| rs7962345 | ENSG00000189182.5 | KRT77 | 12_53762887_C_T_b37 | 665640 | 131 | 0.222 | 0.630 | 0.043 | 0.089 |
| rs7962345 | ENSG00000167768.4 | KRT1 | 12_53762887_C_T_b37 | 688696 | 131 | 0.222 | 0.364 | -0.045 | 0.049 |
| rs7962345 | ENSG00000172867.3 | KRT2 | 12_53762887_C_T_b37 | 716939 | 131 | 0.222 | 0.166 | -0.104 | 0.075 |
| rs7962345 | ENSG00000186049.4 | KRT73 | 12_53762887_C_T_b37 | 750544 | 131 | 0.222 | 0.687 | 0.027 | 0.067 |
| rs7962345 | ENSG00000257495.1 | RP11-641A6.2 | 12_53762887_C_T_b37 | 767564 | 131 | 0.222 | 0.402 | 0.048 | 0.057 |
| rs7962345 | ENSG00000170486.6 | KRT72 | 12_53762887_C_T_b37 | 767637 | 131 | 0.222 | 0.817 | 0.017 | 0.075 |
| rs7962345 | ENSG00000186081.7 | KRT5 | 12_53762887_C_T_b37 | 848416 | 131 | 0.222 | 0.591 | -0.037 | 0.069 |
| rs7962345 | ENSG00000205420.6 | KRT6A | 12_53762887_C_T_b37 | 875846 | 131 | 0.222 | 0.881 | 0.009 | 0.063 |
| rs7962345 | ENSG00000170465.9 | KRT6C | 12_53762887_C_T_b37 | 895318 | 131 | 0.222 | 0.433 | 0.055 | 0.070 |
| rs7962345 | ENSG00000185479.5 | KRT6B | 12_53762887_C_T_b37 | 916977 | 131 | 0.222 | 0.751 | 0.024 | 0.077 |
| rs12903035 | ENSG00000206190.7 | ATP10A | 15_26569608_A_G_b37 | 459291 | 206 | 0.333 | 0.627 | -0.014 | 0.029 |
| rs12903035 | ENSG00000114062.13 | UBE3A | 15_26569608_A_G_b37 | 885480 | 206 | 0.333 | 0.383 | -0.018 | 0.020 |
| rs7178189 | ENSG00000206190.7 | ATP10A | 15_26570398_T_G_b37 | 460081 | 204 | 0.331 | 0.709 | -0.011 | 0.029 |
| rs7178189 | ENSG00000114062.13 | UBE3A | 15_26570398_T_G_b37 | 886270 | 204 | 0.331 | 0.339 | -0.019 | 0.020 |
| rs6576559 | ENSG00000206190.7 | ATP10A | 15_26571091_G_A_b37 | 460774 | 207 | 0.333 | 0.622 | -0.014 | 0.029 |
| rs6576559 | ENSG00000114062.13 | UBE3A | 15_26571091_G_A_b37 | 886963 | 207 | 0.333 | 0.375 | -0.018 | 0.020 |
| rs10873625 | ENSG00000206190.7 | ATP10A | 15_26571202_C_T_b37 | 460885 | 206 | 0.332 | 0.594 | -0.015 | 0.029 |
| rs10873625 | ENSG00000114062.13 | UBE3A | 15_26571202_C_T_b37 | 887074 | 206 | 0.332 | 0.332 | -0.019 | 0.020 |
| rs4523900 | ENSG00000206190.7 | ATP10A | 15_26572014_C_T_b37 | 461697 | 205 | 0.330 | 0.712 | -0.011 | 0.029 |
| rs4523900 | ENSG00000114062.13 | UBE3A | 15_26572014_C_T_b37 | 887886 | 205 | 0.330 | 0.271 | -0.022 | 0.020 |
| rs11853274 | ENSG00000206190.7 | ATP10A | 15_26572363_T_A_b37 | 462046 | 205 | 0.332 | 0.545 | -0.017 | 0.028 |
| rs11853274 | ENSG00000114062.13 | UBE3A | 15_26572363_T_A_b37 | 888235 | 205 | 0.332 | 0.354 | -0.019 | 0.020 |
| rs7169552 | ENSG00000206190.7 | ATP10A | 15_26572981_T_C_b37 | 462664 | 201 | 0.329 | 0.466 | -0.021 | 0.029 |
| rs7169552 | ENSG00000114062.13 | UBE3A | 15_26572981_T_C_b37 | 888853 | 201 | 0.329 | 0.388 | -0.018 | 0.020 |
| rs12442453 | ENSG00000206190.7 | ATP10A | 15_26574726_C_A_b37 | 464409 | 210 | 0.341 | 0.747 | -0.009 | 0.028 |
| rs12442453 | ENSG00000114062.13 | UBE3A | 15_26574726_C_A_b37 | 890598 | 210 | 0.341 | 0.335 | -0.019 | 0.020 |
| rs7171237 | ENSG00000206190.7 | ATP10A | 15_26575300_A_G_b37 | 464983 | 211 | 0.341 | 0.755 | -0.009 | 0.028 |
| rs7171237 | ENSG00000114062.13 | UBE3A | 15_26575300_A_G_b37 | 891172 | 211 | 0.341 | 0.328 | -0.019 | 0.020 |
| rs9920736 | ENSG00000206190.7 | ATP10A | 15_26576430_A_C_b37 | 466113 | 213 | 0.346 | 0.921 | -0.003 | 0.028 |
| rs9920736 | ENSG00000114062.13 | UBE3A | 15_26576430_A_C_b37 | 892302 | 213 | 0.346 | 0.430 | -0.016 | 0.020 |
| rs6576561 | ENSG00000206190.7 | ATP10A | 15_26577629_A_G_b37 | 467312 | 200 | 0.320 | 0.608 | -0.015 | 0.029 |
| rs6576561 | ENSG00000114062.13 | UBE3A | 15_26577629_A_G_b37 | 893501 | 200 | 0.320 | 0.219 | -0.025 | 0.020 |
| rs6576562 | ENSG00000206190.7 | ATP10A | 15_26578036_C_T_b37 | 467719 | 205 | 0.329 | 0.964 | -0.001 | 0.029 |
| rs6576562 | ENSG00000114062.13 | UBE3A | 15_26578036_C_T_b37 | 893908 | 205 | 0.329 | 0.199 | -0.026 | 0.020 |
| rs6576563 | ENSG00000206190.7 | ATP10A | 15_26578079_C_A_b37 | 467762 | 204 | 0.329 | 0.956 | -0.002 | 0.029 |
| rs6576563 | ENSG00000114062.13 | UBE3A | 15_26578079_C_A_b37 | 893951 | 204 | 0.329 | 0.209 | -0.025 | 0.020 |
| rs6576564 | ENSG00000206190.7 | ATP10A | 15_26578154_G_C_b37 | 467837 | 205 | 0.329 | 0.964 | -0.001 | 0.029 |
| rs6576564 | ENSG00000114062.13 | UBE3A | 15_26578154_G_C_b37 | 894026 | 205 | 0.329 | 0.199 | -0.026 | 0.020 |
| rs7168118 | ENSG00000206190.7 | ATP10A | 15_26578376_A_G_b37 | 468059 | 205 | 0.329 | 0.964 | -0.001 | 0.029 |
| rs7168118 | ENSG00000114062.13 | UBE3A | 15_26578376_A_G_b37 | 894248 | 205 | 0.329 | 0.199 | -0.026 | 0.020 |
| rs7168485 | ENSG00000206190.7 | ATP10A | 15_26578530_A_G_b37 | 468213 | 205 | 0.329 | 0.964 | -0.001 | 0.029 |
| rs7168485 | ENSG00000114062.13 | UBE3A | 15_26578530_A_G_b37 | 894402 | 205 | 0.329 | 0.199 | -0.026 | 0.020 |
| rs4365262 | ENSG00000206190.7 | ATP10A | 15_26579982_A_G_b37 | 469665 | 205 | 0.329 | 0.964 | -0.001 | 0.029 |
| rs4365262 | ENSG00000114062.13 | UBE3A | 15_26579982_A_G_b37 | 895854 | 205 | 0.329 | 0.199 | -0.026 | 0.020 |
| rs8039457 | ENSG00000206190.7 | ATP10A | 15_26580823_G_T_b37 | 470506 | 205 | 0.329 | 0.964 | -0.001 | 0.029 |
| rs8039457 | ENSG00000114062.13 | UBE3A | 15_26580823_G_T_b37 | 896695 | 205 | 0.329 | 0.199 | -0.026 | 0.020 |
| rs8041307 | ENSG00000206190.7 | ATP10A | 15_26580842_A_G_b37 | 470525 | 205 | 0.329 | 0.964 | -0.001 | 0.029 |
| rs8041307 | ENSG00000114062.13 | UBE3A | 15_26580842_A_G_b37 | 896714 | 205 | 0.329 | 0.199 | -0.026 | 0.020 |
| rs12717759 | ENSG00000206190.7 | ATP10A | 15_26581883_T_C_b37 | 471566 | 200 | 0.319 | 0.991 | 0.000 | 0.029 |
| rs12717759 | ENSG00000114062.13 | UBE3A | 15_26581883_T_C_b37 | 897755 | 200 | 0.319 | 0.309 | -0.021 | 0.020 |
| rs7177522 | ENSG00000206190.7 | ATP10A | 15_26583425_A_C_b37 | 473108 | 199 | 0.318 | 0.975 | -0.001 | 0.029 |
| rs7177522 | ENSG00000114062.13 | UBE3A | 15_26583425_A_C_b37 | 899297 | 199 | 0.318 | 0.311 | -0.021 | 0.020 |
| rs6576565 | ENSG00000206190.7 | ATP10A | 15_26584959_T_C_b37 | 474642 | 200 | 0.318 | 0.992 | 0.000 | 0.029 |
| rs6576565 | ENSG00000114062.13 | UBE3A | 15_26584959_T_C_b37 | 900831 | 200 | 0.318 | 0.288 | -0.022 | 0.020 |
| rs10517574 | ENSG00000171564.7 | FGB | 4_154510843_G_A_b37 | -973265 | 180 | 0.294 | 0.492 | 0.031 | 0.046 |
| rs10517574 | ENSG00000171566.7 | PLRG1 | 4_154510843_G_A_b37 | -960744 | 180 | 0.294 | 0.151 | -0.036 | 0.025 |
| rs10517574 | ENSG00000145423.4 | SFRP2 | 4_154510843_G_A_b37 | -199429 | 180 | 0.294 | 0.043 | 0.099 | 0.049 |
| rs10517574 | ENSG00000145428.10 | RNF175 | 4_154510843_G_A_b37 | -170544 | 180 | 0.294 | 0.275 | -0.025 | 0.023 |
| rs10517574 | ENSG00000249309.1 | RP11-153M7.5 | 4_154510843_G_A_b37 | -130636 | 180 | 0.294 | 0.660 | -0.016 | 0.036 |
| rs10517574 | ENSG00000137462.6 | TLR2 | 4_154510843_G_A_b37 | -111809 | 180 | 0.294 | 0.142 | -0.032 | 0.022 |
| rs10517574 | ENSG00000248208.1 | RP11-153M7.1 | 4_154510843_G_A_b37 | -68881 | 180 | 0.294 | 0.422 | -0.047 | 0.058 |
| rs10517574 | ENSG00000250771.2 | RP11-153M7.3 | 4_154510843_G_A_b37 | -50477 | 180 | 0.294 | 0.379 | 0.046 | 0.052 |
| rs10517574 | ENSG00000121210.11 | KIAA0922 | 4_154510843_G_A_b37 | 123345 | 180 | 0.294 | 0.381 | -0.031 | 0.036 |
| rs10517574 | ENSG00000121211.3 | MND1 | 4_154510843_G_A_b37 | 245042 | 180 | 0.294 | 0.589 | 0.027 | 0.050 |
| rs10517574 | ENSG00000109654.10 | TRIM2 | 4_154510843_G_A_b37 | 437349 | 180 | 0.294 | 0.323 | 0.055 | 0.055 |
| rs10517574 | ENSG00000137460.4 | FHDC1 | 4_154510843_G_A_b37 | 653339 | 180 | 0.294 | 0.372 | -0.037 | 0.042 |
| rs10517574 | ENSG00000231841.1 | RP11-206F17.2 | 4_154510843_G_A_b37 | 662245 | 180 | 0.294 | 0.999 | 0.000 | 0.066 |
| rs10517574 | ENSG00000164144.10 | ARFIP1 | 4_154510843_G_A_b37 | 809754 | 180 | 0.294 | 0.069 | -0.063 | 0.035 |
| rs10517574 | ENSG00000170006.7 | TMEM154 | 4_154510843_G_A_b37 | 909526 | 180 | 0.294 | 0.996 | 0.000 | 0.019 |
| rs10517574 | ENSG00000248571.1 | RP11-768B22.2 | 4_154510843_G_A_b37 | 923323 | 180 | 0.294 | 0.383 | 0.056 | 0.064 |
| rs10517574 | ENSG00000240669.1 | RP11-555K12.2 | 4_154510843_G_A_b37 | 975358 | 180 | 0.294 | 0.462 | 0.051 | 0.070 |
| rs36068308 | ENSG00000171564.7 | FGB | 4_154511538_G_A_b37 | -972570 | 188 | 0.313 | 0.479 | 0.031 | 0.044 |
| rs36068308 | ENSG00000171566.7 | PLRG1 | 4_154511538_G_A_b37 | -960049 | 188 | 0.313 | 0.151 | -0.034 | 0.024 |
| rs36068308 | ENSG00000145423.4 | SFRP2 | 4_154511538_G_A_b37 | -198734 | 188 | 0.313 | 0.098 | 0.077 | 0.047 |
| rs36068308 | ENSG00000145428.10 | RNF175 | 4_154511538_G_A_b37 | -169849 | 188 | 0.313 | 0.251 | -0.025 | 0.022 |
| rs36068308 | ENSG00000249309.1 | RP11-153M7.5 | 4_154511538_G_A_b37 | -129941 | 188 | 0.313 | 0.718 | -0.013 | 0.035 |
| rs36068308 | ENSG00000137462.6 | TLR2 | 4_154511538_G_A_b37 | -111114 | 188 | 0.313 | 0.274 | -0.023 | 0.021 |
| rs36068308 | ENSG00000248208.1 | RP11-153M7.1 | 4_154511538_G_A_b37 | -68186 | 188 | 0.313 | 0.422 | -0.045 | 0.055 |
| rs36068308 | ENSG00000250771.2 | RP11-153M7.3 | 4_154511538_G_A_b37 | -49782 | 188 | 0.313 | 0.477 | 0.036 | 0.050 |
| rs36068308 | ENSG00000121210.11 | KIAA0922 | 4_154511538_G_A_b37 | 124040 | 188 | 0.313 | 0.170 | -0.047 | 0.034 |
| rs36068308 | ENSG00000121211.3 | MND1 | 4_154511538_G_A_b37 | 245737 | 188 | 0.313 | 0.573 | 0.027 | 0.047 |
| rs36068308 | ENSG00000109654.10 | TRIM2 | 4_154511538_G_A_b37 | 438044 | 188 | 0.313 | 0.695 | 0.021 | 0.053 |
| rs36068308 | ENSG00000137460.4 | FHDC1 | 4_154511538_G_A_b37 | 654034 | 188 | 0.313 | 0.406 | -0.033 | 0.040 |
| rs36068308 | ENSG00000231841.1 | RP11-206F17.2 | 4_154511538_G_A_b37 | 662940 | 188 | 0.313 | 0.869 | -0.010 | 0.063 |
| rs36068308 | ENSG00000164144.10 | ARFIP1 | 4_154511538_G_A_b37 | 810449 | 188 | 0.313 | 0.075 | -0.059 | 0.033 |
| rs36068308 | ENSG00000170006.7 | TMEM154 | 4_154511538_G_A_b37 | 910221 | 188 | 0.313 | 0.969 | 0.001 | 0.018 |
| rs36068308 | ENSG00000248571.1 | RP11-768B22.2 | 4_154511538_G_A_b37 | 924018 | 188 | 0.313 | 0.414 | 0.050 | 0.061 |
| rs36068308 | ENSG00000240669.1 | RP11-555K12.2 | 4_154511538_G_A_b37 | 976053 | 188 | 0.313 | 0.717 | 0.024 | 0.067 |
| rs34076653 | ENSG00000171564.7 | FGB | 4_154511581_C_T_b37 | -972527 | 187 | 0.310 | 0.426 | 0.035 | 0.044 |
| rs34076653 | ENSG00000171566.7 | PLRG1 | 4_154511581_C_T_b37 | -960006 | 187 | 0.310 | 0.117 | -0.037 | 0.024 |
| rs34076653 | ENSG00000145423.4 | SFRP2 | 4_154511581_C_T_b37 | -198691 | 187 | 0.310 | 0.038 | 0.097 | 0.047 |
| rs34076653 | ENSG00000145428.10 | RNF175 | 4_154511581_C_T_b37 | -169806 | 187 | 0.310 | 0.297 | -0.023 | 0.022 |
| rs34076653 | ENSG00000249309.1 | RP11-153M7.5 | 4_154511581_C_T_b37 | -129898 | 187 | 0.310 | 0.620 | -0.017 | 0.035 |
| rs34076653 | ENSG00000137462.6 | TLR2 | 4_154511581_C_T_b37 | -111071 | 187 | 0.310 | 0.341 | -0.020 | 0.021 |
| rs34076653 | ENSG00000248208.1 | RP11-153M7.1 | 4_154511581_C_T_b37 | -68143 | 187 | 0.310 | 0.541 | -0.034 | 0.056 |
| rs34076653 | ENSG00000250771.2 | RP11-153M7.3 | 4_154511581_C_T_b37 | -49739 | 187 | 0.310 | 0.437 | 0.039 | 0.050 |
| rs34076653 | ENSG00000121210.11 | KIAA0922 | 4_154511581_C_T_b37 | 124083 | 187 | 0.310 | 0.169 | -0.047 | 0.034 |
| rs34076653 | ENSG00000121211.3 | MND1 | 4_154511581_C_T_b37 | 245780 | 187 | 0.310 | 0.524 | 0.030 | 0.048 |
| rs34076653 | ENSG00000109654.10 | TRIM2 | 4_154511581_C_T_b37 | 438087 | 187 | 0.310 | 0.819 | 0.012 | 0.053 |
| rs34076653 | ENSG00000137460.4 | FHDC1 | 4_154511581_C_T_b37 | 654077 | 187 | 0.310 | 0.590 | -0.022 | 0.040 |
| rs34076653 | ENSG00000231841.1 | RP11-206F17.2 | 4_154511581_C_T_b37 | 662983 | 187 | 0.310 | 0.798 | -0.016 | 0.063 |
| rs34076653 | ENSG00000164144.10 | ARFIP1 | 4_154511581_C_T_b37 | 810492 | 187 | 0.310 | 0.120 | -0.052 | 0.033 |
| rs34076653 | ENSG00000170006.7 | TMEM154 | 4_154511581_C_T_b37 | 910264 | 187 | 0.310 | 0.930 | 0.002 | 0.018 |
| rs34076653 | ENSG00000248571.1 | RP11-768B22.2 | 4_154511581_C_T_b37 | 924061 | 187 | 0.310 | 0.408 | 0.051 | 0.061 |
| rs34076653 | ENSG00000240669.1 | RP11-555K12.2 | 4_154511581_C_T_b37 | 976096 | 187 | 0.310 | 0.737 | 0.023 | 0.067 |
| rs62324755 | ENSG00000171564.7 | FGB | 4_154511832_A_G_b37 | -972276 | 188 | 0.313 | 0.479 | 0.031 | 0.044 |
| rs62324755 | ENSG00000171566.7 | PLRG1 | 4_154511832_A_G_b37 | -959755 | 188 | 0.313 | 0.151 | -0.034 | 0.024 |
| rs62324755 | ENSG00000145423.4 | SFRP2 | 4_154511832_A_G_b37 | -198440 | 188 | 0.313 | 0.098 | 0.077 | 0.047 |
| rs62324755 | ENSG00000145428.10 | RNF175 | 4_154511832_A_G_b37 | -169555 | 188 | 0.313 | 0.251 | -0.025 | 0.022 |
| rs62324755 | ENSG00000249309.1 | RP11-153M7.5 | 4_154511832_A_G_b37 | -129647 | 188 | 0.313 | 0.718 | -0.013 | 0.035 |
| rs62324755 | ENSG00000137462.6 | TLR2 | 4_154511832_A_G_b37 | -110820 | 188 | 0.313 | 0.274 | -0.023 | 0.021 |
| rs62324755 | ENSG00000248208.1 | RP11-153M7.1 | 4_154511832_A_G_b37 | -67892 | 188 | 0.313 | 0.422 | -0.045 | 0.055 |
| rs62324755 | ENSG00000250771.2 | RP11-153M7.3 | 4_154511832_A_G_b37 | -49488 | 188 | 0.313 | 0.477 | 0.036 | 0.050 |
| rs62324755 | ENSG00000121210.11 | KIAA0922 | 4_154511832_A_G_b37 | 124334 | 188 | 0.313 | 0.170 | -0.047 | 0.034 |
| rs62324755 | ENSG00000121211.3 | MND1 | 4_154511832_A_G_b37 | 246031 | 188 | 0.313 | 0.573 | 0.027 | 0.047 |
| rs62324755 | ENSG00000109654.10 | TRIM2 | 4_154511832_A_G_b37 | 438338 | 188 | 0.313 | 0.695 | 0.021 | 0.053 |
| rs62324755 | ENSG00000137460.4 | FHDC1 | 4_154511832_A_G_b37 | 654328 | 188 | 0.313 | 0.406 | -0.033 | 0.040 |
| rs62324755 | ENSG00000231841.1 | RP11-206F17.2 | 4_154511832_A_G_b37 | 663234 | 188 | 0.313 | 0.869 | -0.010 | 0.063 |
| rs62324755 | ENSG00000164144.10 | ARFIP1 | 4_154511832_A_G_b37 | 810743 | 188 | 0.313 | 0.075 | -0.059 | 0.033 |
| rs62324755 | ENSG00000170006.7 | TMEM154 | 4_154511832_A_G_b37 | 910515 | 188 | 0.313 | 0.969 | 0.001 | 0.018 |
| rs62324755 | ENSG00000248571.1 | RP11-768B22.2 | 4_154511832_A_G_b37 | 924312 | 188 | 0.313 | 0.414 | 0.050 | 0.061 |
| rs62324755 | ENSG00000240669.1 | RP11-555K12.2 | 4_154511832_A_G_b37 | 976347 | 188 | 0.313 | 0.717 | 0.024 | 0.067 |
| rs17279148 | ENSG00000171560.10 | FGA | 4_154512437_A_G_b37 | -999481 | 189 | 0.316 | 0.177 | 0.064 | 0.047 |
| rs17279148 | ENSG00000171564.7 | FGB | 4_154512437_A_G_b37 | -971671 | 189 | 0.316 | 0.449 | 0.033 | 0.044 |
| rs17279148 | ENSG00000171566.7 | PLRG1 | 4_154512437_A_G_b37 | -959150 | 189 | 0.316 | 0.125 | -0.036 | 0.024 |
| rs17279148 | ENSG00000145423.4 | SFRP2 | 4_154512437_A_G_b37 | -197835 | 189 | 0.316 | 0.082 | 0.081 | 0.046 |
| rs17279148 | ENSG00000145428.10 | RNF175 | 4_154512437_A_G_b37 | -168950 | 189 | 0.316 | 0.264 | -0.024 | 0.022 |
| rs17279148 | ENSG00000249309.1 | RP11-153M7.5 | 4_154512437_A_G_b37 | -129042 | 189 | 0.316 | 0.728 | -0.012 | 0.035 |
| rs17279148 | ENSG00000137462.6 | TLR2 | 4_154512437_A_G_b37 | -110215 | 189 | 0.316 | 0.260 | -0.024 | 0.021 |
| rs17279148 | ENSG00000248208.1 | RP11-153M7.1 | 4_154512437_A_G_b37 | -67287 | 189 | 0.316 | 0.444 | -0.042 | 0.055 |
| rs17279148 | ENSG00000250771.2 | RP11-153M7.3 | 4_154512437_A_G_b37 | -48883 | 189 | 0.316 | 0.580 | 0.028 | 0.050 |
| rs17279148 | ENSG00000121210.11 | KIAA0922 | 4_154512437_A_G_b37 | 124939 | 189 | 0.316 | 0.129 | -0.052 | 0.034 |
| rs17279148 | ENSG00000121211.3 | MND1 | 4_154512437_A_G_b37 | 246636 | 189 | 0.316 | 0.573 | 0.027 | 0.047 |
| rs17279148 | ENSG00000109654.10 | TRIM2 | 4_154512437_A_G_b37 | 438943 | 189 | 0.316 | 0.739 | 0.018 | 0.053 |
| rs17279148 | ENSG00000137460.4 | FHDC1 | 4_154512437_A_G_b37 | 654933 | 189 | 0.316 | 0.396 | -0.034 | 0.040 |
| rs17279148 | ENSG00000231841.1 | RP11-206F17.2 | 4_154512437_A_G_b37 | 663839 | 189 | 0.316 | 0.849 | -0.012 | 0.063 |
| rs17279148 | ENSG00000164144.10 | ARFIP1 | 4_154512437_A_G_b37 | 811348 | 189 | 0.316 | 0.064 | -0.061 | 0.033 |
| rs17279148 | ENSG00000170006.7 | TMEM154 | 4_154512437_A_G_b37 | 911120 | 189 | 0.316 | 0.916 | 0.002 | 0.018 |
| rs17279148 | ENSG00000248571.1 | RP11-768B22.2 | 4_154512437_A_G_b37 | 924917 | 189 | 0.316 | 0.367 | 0.055 | 0.061 |
| rs17279148 | ENSG00000240669.1 | RP11-555K12.2 | 4_154512437_A_G_b37 | 976952 | 189 | 0.316 | 0.798 | 0.017 | 0.067 |
| rs1371160 | ENSG00000171560.10 | FGA | 4_154512656_C_T_b37 | -999262 | 189 | 0.316 | 0.177 | 0.064 | 0.047 |
| rs1371160 | ENSG00000171564.7 | FGB | 4_154512656_C_T_b37 | -971452 | 189 | 0.316 | 0.449 | 0.033 | 0.044 |
| rs1371160 | ENSG00000171566.7 | PLRG1 | 4_154512656_C_T_b37 | -958931 | 189 | 0.316 | 0.125 | -0.036 | 0.024 |
| rs1371160 | ENSG00000145423.4 | SFRP2 | 4_154512656_C_T_b37 | -197616 | 189 | 0.316 | 0.082 | 0.081 | 0.046 |
| rs1371160 | ENSG00000145428.10 | RNF175 | 4_154512656_C_T_b37 | -168731 | 189 | 0.316 | 0.264 | -0.024 | 0.022 |
| rs1371160 | ENSG00000249309.1 | RP11-153M7.5 | 4_154512656_C_T_b37 | -128823 | 189 | 0.316 | 0.728 | -0.012 | 0.035 |
| rs1371160 | ENSG00000137462.6 | TLR2 | 4_154512656_C_T_b37 | -109996 | 189 | 0.316 | 0.260 | -0.024 | 0.021 |
| rs1371160 | ENSG00000248208.1 | RP11-153M7.1 | 4_154512656_C_T_b37 | -67068 | 189 | 0.316 | 0.444 | -0.042 | 0.055 |
| rs1371160 | ENSG00000250771.2 | RP11-153M7.3 | 4_154512656_C_T_b37 | -48664 | 189 | 0.316 | 0.580 | 0.028 | 0.050 |
| rs1371160 | ENSG00000121210.11 | KIAA0922 | 4_154512656_C_T_b37 | 125158 | 189 | 0.316 | 0.129 | -0.052 | 0.034 |
| rs1371160 | ENSG00000121211.3 | MND1 | 4_154512656_C_T_b37 | 246855 | 189 | 0.316 | 0.573 | 0.027 | 0.047 |
| rs1371160 | ENSG00000109654.10 | TRIM2 | 4_154512656_C_T_b37 | 439162 | 189 | 0.316 | 0.739 | 0.018 | 0.053 |
| rs1371160 | ENSG00000137460.4 | FHDC1 | 4_154512656_C_T_b37 | 655152 | 189 | 0.316 | 0.396 | -0.034 | 0.040 |
| rs1371160 | ENSG00000231841.1 | RP11-206F17.2 | 4_154512656_C_T_b37 | 664058 | 189 | 0.316 | 0.849 | -0.012 | 0.063 |
| rs1371160 | ENSG00000164144.10 | ARFIP1 | 4_154512656_C_T_b37 | 811567 | 189 | 0.316 | 0.064 | -0.061 | 0.033 |
| rs1371160 | ENSG00000170006.7 | TMEM154 | 4_154512656_C_T_b37 | 911339 | 189 | 0.316 | 0.916 | 0.002 | 0.018 |
| rs1371160 | ENSG00000248571.1 | RP11-768B22.2 | 4_154512656_C_T_b37 | 925136 | 189 | 0.316 | 0.367 | 0.055 | 0.061 |
| rs1371160 | ENSG00000240669.1 | RP11-555K12.2 | 4_154512656_C_T_b37 | 977171 | 189 | 0.316 | 0.798 | 0.017 | 0.067 |
| rs7664062 | ENSG00000171560.10 | FGA | 4_154513147_C_T_b37 | -998771 | 189 | 0.316 | 0.177 | 0.064 | 0.047 |
| rs7664062 | ENSG00000171564.7 | FGB | 4_154513147_C_T_b37 | -970961 | 189 | 0.316 | 0.449 | 0.033 | 0.044 |
| rs7664062 | ENSG00000171566.7 | PLRG1 | 4_154513147_C_T_b37 | -958440 | 189 | 0.316 | 0.125 | -0.036 | 0.024 |
| rs7664062 | ENSG00000145423.4 | SFRP2 | 4_154513147_C_T_b37 | -197125 | 189 | 0.316 | 0.082 | 0.081 | 0.046 |
| rs7664062 | ENSG00000145428.10 | RNF175 | 4_154513147_C_T_b37 | -168240 | 189 | 0.316 | 0.264 | -0.024 | 0.022 |
| rs7664062 | ENSG00000249309.1 | RP11-153M7.5 | 4_154513147_C_T_b37 | -128332 | 189 | 0.316 | 0.728 | -0.012 | 0.035 |
| rs7664062 | ENSG00000137462.6 | TLR2 | 4_154513147_C_T_b37 | -109505 | 189 | 0.316 | 0.260 | -0.024 | 0.021 |
| rs7664062 | ENSG00000248208.1 | RP11-153M7.1 | 4_154513147_C_T_b37 | -66577 | 189 | 0.316 | 0.444 | -0.042 | 0.055 |
| rs7664062 | ENSG00000250771.2 | RP11-153M7.3 | 4_154513147_C_T_b37 | -48173 | 189 | 0.316 | 0.580 | 0.028 | 0.050 |
| rs7664062 | ENSG00000121210.11 | KIAA0922 | 4_154513147_C_T_b37 | 125649 | 189 | 0.316 | 0.129 | -0.052 | 0.034 |
| rs7664062 | ENSG00000121211.3 | MND1 | 4_154513147_C_T_b37 | 247346 | 189 | 0.316 | 0.573 | 0.027 | 0.047 |
| rs7664062 | ENSG00000109654.10 | TRIM2 | 4_154513147_C_T_b37 | 439653 | 189 | 0.316 | 0.739 | 0.018 | 0.053 |
| rs7664062 | ENSG00000137460.4 | FHDC1 | 4_154513147_C_T_b37 | 655643 | 189 | 0.316 | 0.396 | -0.034 | 0.040 |
| rs7664062 | ENSG00000231841.1 | RP11-206F17.2 | 4_154513147_C_T_b37 | 664549 | 189 | 0.316 | 0.849 | -0.012 | 0.063 |
| rs7664062 | ENSG00000164144.10 | ARFIP1 | 4_154513147_C_T_b37 | 812058 | 189 | 0.316 | 0.064 | -0.061 | 0.033 |
| rs7664062 | ENSG00000170006.7 | TMEM154 | 4_154513147_C_T_b37 | 911830 | 189 | 0.316 | 0.916 | 0.002 | 0.018 |
| rs7664062 | ENSG00000248571.1 | RP11-768B22.2 | 4_154513147_C_T_b37 | 925627 | 189 | 0.316 | 0.367 | 0.055 | 0.061 |
| rs7664062 | ENSG00000240669.1 | RP11-555K12.2 | 4_154513147_C_T_b37 | 977662 | 189 | 0.316 | 0.798 | 0.017 | 0.067 |
| rs62324756 | ENSG00000171560.10 | FGA | 4_154513281_C_T_b37 | -998637 | 187 | 0.316 | 0.126 | 0.073 | 0.048 |
| rs62324756 | ENSG00000171564.7 | FGB | 4_154513281_C_T_b37 | -970827 | 187 | 0.316 | 0.413 | 0.036 | 0.044 |
| rs62324756 | ENSG00000171566.7 | PLRG1 | 4_154513281_C_T_b37 | -958306 | 187 | 0.316 | 0.092 | -0.040 | 0.024 |
| rs62324756 | ENSG00000145423.4 | SFRP2 | 4_154513281_C_T_b37 | -196991 | 187 | 0.316 | 0.110 | 0.075 | 0.047 |
| rs62324756 | ENSG00000145428.10 | RNF175 | 4_154513281_C_T_b37 | -168106 | 187 | 0.316 | 0.377 | -0.019 | 0.022 |
| rs62324756 | ENSG00000249309.1 | RP11-153M7.5 | 4_154513281_C_T_b37 | -128198 | 187 | 0.316 | 0.774 | -0.010 | 0.035 |
| rs62324756 | ENSG00000137462.6 | TLR2 | 4_154513281_C_T_b37 | -109371 | 187 | 0.316 | 0.269 | -0.023 | 0.021 |
| rs62324756 | ENSG00000248208.1 | RP11-153M7.1 | 4_154513281_C_T_b37 | -66443 | 187 | 0.316 | 0.418 | -0.045 | 0.056 |
| rs62324756 | ENSG00000250771.2 | RP11-153M7.3 | 4_154513281_C_T_b37 | -48039 | 187 | 0.316 | 0.571 | 0.029 | 0.050 |
| rs62324756 | ENSG00000121210.11 | KIAA0922 | 4_154513281_C_T_b37 | 125783 | 187 | 0.316 | 0.131 | -0.052 | 0.034 |
| rs62324756 | ENSG00000121211.3 | MND1 | 4_154513281_C_T_b37 | 247480 | 187 | 0.316 | 0.521 | 0.031 | 0.048 |
| rs62324756 | ENSG00000109654.10 | TRIM2 | 4_154513281_C_T_b37 | 439787 | 187 | 0.316 | 0.906 | 0.006 | 0.053 |
| rs62324756 | ENSG00000137460.4 | FHDC1 | 4_154513281_C_T_b37 | 655777 | 187 | 0.316 | 0.384 | -0.035 | 0.040 |
| rs62324756 | ENSG00000231841.1 | RP11-206F17.2 | 4_154513281_C_T_b37 | 664683 | 187 | 0.316 | 0.785 | -0.017 | 0.063 |
| rs62324756 | ENSG00000164144.10 | ARFIP1 | 4_154513281_C_T_b37 | 812192 | 187 | 0.316 | 0.066 | -0.061 | 0.033 |
| rs62324756 | ENSG00000170006.7 | TMEM154 | 4_154513281_C_T_b37 | 911964 | 187 | 0.316 | 0.935 | 0.002 | 0.018 |
| rs62324756 | ENSG00000248571.1 | RP11-768B22.2 | 4_154513281_C_T_b37 | 925761 | 187 | 0.316 | 0.421 | 0.049 | 0.061 |
| rs62324756 | ENSG00000240669.1 | RP11-555K12.2 | 4_154513281_C_T_b37 | 977796 | 187 | 0.316 | 0.821 | 0.015 | 0.067 |
| rs1371158 | ENSG00000171560.10 | FGA | 4_154513531_A_G_b37 | -998387 | 188 | 0.315 | 0.174 | 0.064 | 0.047 |
| rs1371158 | ENSG00000171564.7 | FGB | 4_154513531_A_G_b37 | -970577 | 188 | 0.315 | 0.456 | 0.033 | 0.044 |
| rs1371158 | ENSG00000171566.7 | PLRG1 | 4_154513531_A_G_b37 | -958056 | 188 | 0.315 | 0.120 | -0.037 | 0.024 |
| rs1371158 | ENSG00000145423.4 | SFRP2 | 4_154513531_A_G_b37 | -196741 | 188 | 0.315 | 0.082 | 0.081 | 0.046 |
| rs1371158 | ENSG00000145428.10 | RNF175 | 4_154513531_A_G_b37 | -167856 | 188 | 0.315 | 0.268 | -0.024 | 0.022 |
| rs1371158 | ENSG00000249309.1 | RP11-153M7.5 | 4_154513531_A_G_b37 | -127948 | 188 | 0.315 | 0.744 | -0.011 | 0.035 |
| rs1371158 | ENSG00000137462.6 | TLR2 | 4_154513531_A_G_b37 | -109121 | 188 | 0.315 | 0.270 | -0.023 | 0.021 |
| rs1371158 | ENSG00000248208.1 | RP11-153M7.1 | 4_154513531_A_G_b37 | -66193 | 188 | 0.315 | 0.447 | -0.042 | 0.055 |
| rs1371158 | ENSG00000250771.2 | RP11-153M7.3 | 4_154513531_A_G_b37 | -47789 | 188 | 0.315 | 0.606 | 0.026 | 0.050 |
| rs1371158 | ENSG00000121210.11 | KIAA0922 | 4_154513531_A_G_b37 | 126033 | 188 | 0.315 | 0.120 | -0.053 | 0.034 |
| rs1371158 | ENSG00000121211.3 | MND1 | 4_154513531_A_G_b37 | 247730 | 188 | 0.315 | 0.562 | 0.027 | 0.047 |
| rs1371158 | ENSG00000109654.10 | TRIM2 | 4_154513531_A_G_b37 | 440037 | 188 | 0.315 | 0.726 | 0.019 | 0.053 |
| rs1371158 | ENSG00000137460.4 | FHDC1 | 4_154513531_A_G_b37 | 656027 | 188 | 0.315 | 0.403 | -0.033 | 0.040 |
| rs1371158 | ENSG00000231841.1 | RP11-206F17.2 | 4_154513531_A_G_b37 | 664933 | 188 | 0.315 | 0.861 | -0.011 | 0.063 |
| rs1371158 | ENSG00000164144.10 | ARFIP1 | 4_154513531_A_G_b37 | 812442 | 188 | 0.315 | 0.068 | -0.061 | 0.033 |
| rs1371158 | ENSG00000170006.7 | TMEM154 | 4_154513531_A_G_b37 | 912214 | 188 | 0.315 | 0.936 | 0.001 | 0.018 |
| rs1371158 | ENSG00000248571.1 | RP11-768B22.2 | 4_154513531_A_G_b37 | 926011 | 188 | 0.315 | 0.376 | 0.054 | 0.061 |
| rs1371158 | ENSG00000240669.1 | RP11-555K12.2 | 4_154513531_A_G_b37 | 978046 | 188 | 0.315 | 0.784 | 0.018 | 0.067 |
| rs7669418 | ENSG00000171560.10 | FGA | 4_154513627_A_G_b37 | -998291 | 189 | 0.316 | 0.177 | 0.064 | 0.047 |
| rs7669418 | ENSG00000171564.7 | FGB | 4_154513627_A_G_b37 | -970481 | 189 | 0.316 | 0.449 | 0.033 | 0.044 |
| rs7669418 | ENSG00000171566.7 | PLRG1 | 4_154513627_A_G_b37 | -957960 | 189 | 0.316 | 0.125 | -0.036 | 0.024 |
| rs7669418 | ENSG00000145423.4 | SFRP2 | 4_154513627_A_G_b37 | -196645 | 189 | 0.316 | 0.082 | 0.081 | 0.046 |
| rs7669418 | ENSG00000145428.10 | RNF175 | 4_154513627_A_G_b37 | -167760 | 189 | 0.316 | 0.264 | -0.024 | 0.022 |
| rs7669418 | ENSG00000249309.1 | RP11-153M7.5 | 4_154513627_A_G_b37 | -127852 | 189 | 0.316 | 0.728 | -0.012 | 0.035 |
| rs7669418 | ENSG00000137462.6 | TLR2 | 4_154513627_A_G_b37 | -109025 | 189 | 0.316 | 0.260 | -0.024 | 0.021 |
| rs7669418 | ENSG00000248208.1 | RP11-153M7.1 | 4_154513627_A_G_b37 | -66097 | 189 | 0.316 | 0.444 | -0.042 | 0.055 |
| rs7669418 | ENSG00000250771.2 | RP11-153M7.3 | 4_154513627_A_G_b37 | -47693 | 189 | 0.316 | 0.580 | 0.028 | 0.050 |
| rs7669418 | ENSG00000121210.11 | KIAA0922 | 4_154513627_A_G_b37 | 126129 | 189 | 0.316 | 0.129 | -0.052 | 0.034 |
| rs7669418 | ENSG00000121211.3 | MND1 | 4_154513627_A_G_b37 | 247826 | 189 | 0.316 | 0.573 | 0.027 | 0.047 |
| rs7669418 | ENSG00000109654.10 | TRIM2 | 4_154513627_A_G_b37 | 440133 | 189 | 0.316 | 0.739 | 0.018 | 0.053 |
| rs7669418 | ENSG00000137460.4 | FHDC1 | 4_154513627_A_G_b37 | 656123 | 189 | 0.316 | 0.396 | -0.034 | 0.040 |
| rs7669418 | ENSG00000231841.1 | RP11-206F17.2 | 4_154513627_A_G_b37 | 665029 | 189 | 0.316 | 0.849 | -0.012 | 0.063 |
| rs7669418 | ENSG00000164144.10 | ARFIP1 | 4_154513627_A_G_b37 | 812538 | 189 | 0.316 | 0.064 | -0.061 | 0.033 |
| rs7669418 | ENSG00000170006.7 | TMEM154 | 4_154513627_A_G_b37 | 912310 | 189 | 0.316 | 0.916 | 0.002 | 0.018 |
| rs7669418 | ENSG00000248571.1 | RP11-768B22.2 | 4_154513627_A_G_b37 | 926107 | 189 | 0.316 | 0.367 | 0.055 | 0.061 |
| rs7669418 | ENSG00000240669.1 | RP11-555K12.2 | 4_154513627_A_G_b37 | 978142 | 189 | 0.316 | 0.798 | 0.017 | 0.067 |
| rs7670353 | ENSG00000171560.10 | FGA | 4_154514059_A_G_b37 | -997859 | 189 | 0.316 | 0.177 | 0.064 | 0.047 |
| rs7670353 | ENSG00000171564.7 | FGB | 4_154514059_A_G_b37 | -970049 | 189 | 0.316 | 0.449 | 0.033 | 0.044 |
| rs7670353 | ENSG00000171566.7 | PLRG1 | 4_154514059_A_G_b37 | -957528 | 189 | 0.316 | 0.125 | -0.036 | 0.024 |
| rs7670353 | ENSG00000145423.4 | SFRP2 | 4_154514059_A_G_b37 | -196213 | 189 | 0.316 | 0.082 | 0.081 | 0.046 |
| rs7670353 | ENSG00000145428.10 | RNF175 | 4_154514059_A_G_b37 | -167328 | 189 | 0.316 | 0.264 | -0.024 | 0.022 |
| rs7670353 | ENSG00000249309.1 | RP11-153M7.5 | 4_154514059_A_G_b37 | -127420 | 189 | 0.316 | 0.728 | -0.012 | 0.035 |
| rs7670353 | ENSG00000137462.6 | TLR2 | 4_154514059_A_G_b37 | -108593 | 189 | 0.316 | 0.260 | -0.024 | 0.021 |
| rs7670353 | ENSG00000248208.1 | RP11-153M7.1 | 4_154514059_A_G_b37 | -65665 | 189 | 0.316 | 0.444 | -0.042 | 0.055 |
| rs7670353 | ENSG00000250771.2 | RP11-153M7.3 | 4_154514059_A_G_b37 | -47261 | 189 | 0.316 | 0.580 | 0.028 | 0.050 |
| rs7670353 | ENSG00000121210.11 | KIAA0922 | 4_154514059_A_G_b37 | 126561 | 189 | 0.316 | 0.129 | -0.052 | 0.034 |
| rs7670353 | ENSG00000121211.3 | MND1 | 4_154514059_A_G_b37 | 248258 | 189 | 0.316 | 0.573 | 0.027 | 0.047 |
| rs7670353 | ENSG00000109654.10 | TRIM2 | 4_154514059_A_G_b37 | 440565 | 189 | 0.316 | 0.739 | 0.018 | 0.053 |
| rs7670353 | ENSG00000137460.4 | FHDC1 | 4_154514059_A_G_b37 | 656555 | 189 | 0.316 | 0.396 | -0.034 | 0.040 |
| rs7670353 | ENSG00000231841.1 | RP11-206F17.2 | 4_154514059_A_G_b37 | 665461 | 189 | 0.316 | 0.849 | -0.012 | 0.063 |
| rs7670353 | ENSG00000164144.10 | ARFIP1 | 4_154514059_A_G_b37 | 812970 | 189 | 0.316 | 0.064 | -0.061 | 0.033 |
| rs7670353 | ENSG00000170006.7 | TMEM154 | 4_154514059_A_G_b37 | 912742 | 189 | 0.316 | 0.916 | 0.002 | 0.018 |
| rs7670353 | ENSG00000248571.1 | RP11-768B22.2 | 4_154514059_A_G_b37 | 926539 | 189 | 0.316 | 0.367 | 0.055 | 0.061 |
| rs7670353 | ENSG00000240669.1 | RP11-555K12.2 | 4_154514059_A_G_b37 | 978574 | 189 | 0.316 | 0.798 | 0.017 | 0.067 |
| rs28386897 | ENSG00000171560.10 | FGA | 4_154514479_A_G_b37 | -997439 | 189 | 0.316 | 0.177 | 0.064 | 0.047 |
| rs28386897 | ENSG00000171564.7 | FGB | 4_154514479_A_G_b37 | -969629 | 189 | 0.316 | 0.449 | 0.033 | 0.044 |
| rs28386897 | ENSG00000171566.7 | PLRG1 | 4_154514479_A_G_b37 | -957108 | 189 | 0.316 | 0.125 | -0.036 | 0.024 |
| rs28386897 | ENSG00000145423.4 | SFRP2 | 4_154514479_A_G_b37 | -195793 | 189 | 0.316 | 0.082 | 0.081 | 0.046 |
| rs28386897 | ENSG00000145428.10 | RNF175 | 4_154514479_A_G_b37 | -166908 | 189 | 0.316 | 0.264 | -0.024 | 0.022 |
| rs28386897 | ENSG00000249309.1 | RP11-153M7.5 | 4_154514479_A_G_b37 | -127000 | 189 | 0.316 | 0.728 | -0.012 | 0.035 |
| rs28386897 | ENSG00000137462.6 | TLR2 | 4_154514479_A_G_b37 | -108173 | 189 | 0.316 | 0.260 | -0.024 | 0.021 |
| rs28386897 | ENSG00000248208.1 | RP11-153M7.1 | 4_154514479_A_G_b37 | -65245 | 189 | 0.316 | 0.444 | -0.042 | 0.055 |
| rs28386897 | ENSG00000250771.2 | RP11-153M7.3 | 4_154514479_A_G_b37 | -46841 | 189 | 0.316 | 0.580 | 0.028 | 0.050 |
| rs28386897 | ENSG00000121210.11 | KIAA0922 | 4_154514479_A_G_b37 | 126981 | 189 | 0.316 | 0.129 | -0.052 | 0.034 |
| rs28386897 | ENSG00000121211.3 | MND1 | 4_154514479_A_G_b37 | 248678 | 189 | 0.316 | 0.573 | 0.027 | 0.047 |
| rs28386897 | ENSG00000109654.10 | TRIM2 | 4_154514479_A_G_b37 | 440985 | 189 | 0.316 | 0.739 | 0.018 | 0.053 |
| rs28386897 | ENSG00000137460.4 | FHDC1 | 4_154514479_A_G_b37 | 656975 | 189 | 0.316 | 0.396 | -0.034 | 0.040 |
| rs28386897 | ENSG00000231841.1 | RP11-206F17.2 | 4_154514479_A_G_b37 | 665881 | 189 | 0.316 | 0.849 | -0.012 | 0.063 |
| rs28386897 | ENSG00000164144.10 | ARFIP1 | 4_154514479_A_G_b37 | 813390 | 189 | 0.316 | 0.064 | -0.061 | 0.033 |
| rs28386897 | ENSG00000170006.7 | TMEM154 | 4_154514479_A_G_b37 | 913162 | 189 | 0.316 | 0.916 | 0.002 | 0.018 |
| rs28386897 | ENSG00000248571.1 | RP11-768B22.2 | 4_154514479_A_G_b37 | 926959 | 189 | 0.316 | 0.367 | 0.055 | 0.061 |
| rs28386897 | ENSG00000240669.1 | RP11-555K12.2 | 4_154514479_A_G_b37 | 978994 | 189 | 0.316 | 0.798 | 0.017 | 0.067 |
| rs13109887 | ENSG00000171560.10 | FGA | 4_154514847_T_C_b37 | -997071 | 183 | 0.305 | 0.157 | 0.069 | 0.049 |
| rs13109887 | ENSG00000171564.7 | FGB | 4_154514847_T_C_b37 | -969261 | 183 | 0.305 | 0.320 | 0.045 | 0.045 |
| rs13109887 | ENSG00000171566.7 | PLRG1 | 4_154514847_T_C_b37 | -956740 | 183 | 0.305 | 0.145 | -0.036 | 0.024 |
| rs13109887 | ENSG00000145423.4 | SFRP2 | 4_154514847_T_C_b37 | -195425 | 183 | 0.305 | 0.034 | 0.102 | 0.048 |
| rs13109887 | ENSG00000145428.10 | RNF175 | 4_154514847_T_C_b37 | -166540 | 183 | 0.305 | 0.253 | -0.026 | 0.022 |
| rs13109887 | ENSG00000249309.1 | RP11-153M7.5 | 4_154514847_T_C_b37 | -126632 | 183 | 0.305 | 0.761 | -0.011 | 0.036 |
| rs13109887 | ENSG00000137462.6 | TLR2 | 4_154514847_T_C_b37 | -107805 | 183 | 0.305 | 0.146 | -0.031 | 0.022 |
| rs13109887 | ENSG00000248208.1 | RP11-153M7.1 | 4_154514847_T_C_b37 | -64877 | 183 | 0.305 | 0.435 | -0.045 | 0.057 |
| rs13109887 | ENSG00000250771.2 | RP11-153M7.3 | 4_154514847_T_C_b37 | -46473 | 183 | 0.305 | 0.379 | 0.045 | 0.051 |
| rs13109887 | ENSG00000121210.11 | KIAA0922 | 4_154514847_T_C_b37 | 127349 | 183 | 0.305 | 0.184 | -0.047 | 0.035 |
| rs13109887 | ENSG00000121211.3 | MND1 | 4_154514847_T_C_b37 | 249046 | 183 | 0.305 | 0.646 | 0.022 | 0.049 |
| rs13109887 | ENSG00000109654.10 | TRIM2 | 4_154514847_T_C_b37 | 441353 | 183 | 0.305 | 0.471 | 0.039 | 0.054 |
| rs13109887 | ENSG00000137460.4 | FHDC1 | 4_154514847_T_C_b37 | 657343 | 183 | 0.305 | 0.355 | -0.038 | 0.041 |
| rs13109887 | ENSG00000231841.1 | RP11-206F17.2 | 4_154514847_T_C_b37 | 666249 | 183 | 0.305 | 0.636 | -0.031 | 0.065 |
| rs13109887 | ENSG00000164144.10 | ARFIP1 | 4_154514847_T_C_b37 | 813758 | 183 | 0.305 | 0.061 | -0.064 | 0.034 |
| rs13109887 | ENSG00000170006.7 | TMEM154 | 4_154514847_T_C_b37 | 913530 | 183 | 0.305 | 0.962 | -0.001 | 0.019 |
| rs13109887 | ENSG00000248571.1 | RP11-768B22.2 | 4_154514847_T_C_b37 | 927327 | 183 | 0.305 | 0.290 | 0.066 | 0.063 |
| rs13109887 | ENSG00000240669.1 | RP11-555K12.2 | 4_154514847_T_C_b37 | 979362 | 183 | 0.305 | 0.621 | 0.034 | 0.069 |
| rs17370297 | ENSG00000171560.10 | FGA | 4_154514965_T_C_b37 | -996953 | 189 | 0.316 | 0.177 | 0.064 | 0.047 |
| rs17370297 | ENSG00000171564.7 | FGB | 4_154514965_T_C_b37 | -969143 | 189 | 0.316 | 0.449 | 0.033 | 0.044 |
| rs17370297 | ENSG00000171566.7 | PLRG1 | 4_154514965_T_C_b37 | -956622 | 189 | 0.316 | 0.125 | -0.036 | 0.024 |
| rs17370297 | ENSG00000145423.4 | SFRP2 | 4_154514965_T_C_b37 | -195307 | 189 | 0.316 | 0.082 | 0.081 | 0.046 |
| rs17370297 | ENSG00000145428.10 | RNF175 | 4_154514965_T_C_b37 | -166422 | 189 | 0.316 | 0.264 | -0.024 | 0.022 |
| rs17370297 | ENSG00000249309.1 | RP11-153M7.5 | 4_154514965_T_C_b37 | -126514 | 189 | 0.316 | 0.728 | -0.012 | 0.035 |
| rs17370297 | ENSG00000137462.6 | TLR2 | 4_154514965_T_C_b37 | -107687 | 189 | 0.316 | 0.260 | -0.024 | 0.021 |
| rs17370297 | ENSG00000248208.1 | RP11-153M7.1 | 4_154514965_T_C_b37 | -64759 | 189 | 0.316 | 0.444 | -0.042 | 0.055 |
| rs17370297 | ENSG00000250771.2 | RP11-153M7.3 | 4_154514965_T_C_b37 | -46355 | 189 | 0.316 | 0.580 | 0.028 | 0.050 |
| rs17370297 | ENSG00000121210.11 | KIAA0922 | 4_154514965_T_C_b37 | 127467 | 189 | 0.316 | 0.129 | -0.052 | 0.034 |
| rs17370297 | ENSG00000121211.3 | MND1 | 4_154514965_T_C_b37 | 249164 | 189 | 0.316 | 0.573 | 0.027 | 0.047 |
| rs17370297 | ENSG00000109654.10 | TRIM2 | 4_154514965_T_C_b37 | 441471 | 189 | 0.316 | 0.739 | 0.018 | 0.053 |
| rs17370297 | ENSG00000137460.4 | FHDC1 | 4_154514965_T_C_b37 | 657461 | 189 | 0.316 | 0.396 | -0.034 | 0.040 |
| rs17370297 | ENSG00000231841.1 | RP11-206F17.2 | 4_154514965_T_C_b37 | 666367 | 189 | 0.316 | 0.849 | -0.012 | 0.063 |
| rs17370297 | ENSG00000164144.10 | ARFIP1 | 4_154514965_T_C_b37 | 813876 | 189 | 0.316 | 0.064 | -0.061 | 0.033 |
| rs17370297 | ENSG00000170006.7 | TMEM154 | 4_154514965_T_C_b37 | 913648 | 189 | 0.316 | 0.916 | 0.002 | 0.018 |
| rs17370297 | ENSG00000248571.1 | RP11-768B22.2 | 4_154514965_T_C_b37 | 927445 | 189 | 0.316 | 0.367 | 0.055 | 0.061 |
| rs17370297 | ENSG00000240669.1 | RP11-555K12.2 | 4_154514965_T_C_b37 | 979480 | 189 | 0.316 | 0.798 | 0.017 | 0.067 |
| rs17370311 | ENSG00000171560.10 | FGA | 4_154515504_G_A_b37 | -996414 | 188 | 0.313 | 0.202 | 0.061 | 0.047 |
| rs17370311 | ENSG00000171564.7 | FGB | 4_154515504_G_A_b37 | -968604 | 188 | 0.313 | 0.479 | 0.031 | 0.044 |
| rs17370311 | ENSG00000171566.7 | PLRG1 | 4_154515504_G_A_b37 | -956083 | 188 | 0.313 | 0.151 | -0.034 | 0.024 |
| rs17370311 | ENSG00000145423.4 | SFRP2 | 4_154515504_G_A_b37 | -194768 | 188 | 0.313 | 0.098 | 0.077 | 0.047 |
| rs17370311 | ENSG00000145428.10 | RNF175 | 4_154515504_G_A_b37 | -165883 | 188 | 0.313 | 0.251 | -0.025 | 0.022 |
| rs17370311 | ENSG00000249309.1 | RP11-153M7.5 | 4_154515504_G_A_b37 | -125975 | 188 | 0.313 | 0.718 | -0.013 | 0.035 |
| rs17370311 | ENSG00000137462.6 | TLR2 | 4_154515504_G_A_b37 | -107148 | 188 | 0.313 | 0.274 | -0.023 | 0.021 |
| rs17370311 | ENSG00000248208.1 | RP11-153M7.1 | 4_154515504_G_A_b37 | -64220 | 188 | 0.313 | 0.422 | -0.045 | 0.055 |
| rs17370311 | ENSG00000250771.2 | RP11-153M7.3 | 4_154515504_G_A_b37 | -45816 | 188 | 0.313 | 0.477 | 0.036 | 0.050 |
| rs17370311 | ENSG00000121210.11 | KIAA0922 | 4_154515504_G_A_b37 | 128006 | 188 | 0.313 | 0.170 | -0.047 | 0.034 |
| rs17370311 | ENSG00000121211.3 | MND1 | 4_154515504_G_A_b37 | 249703 | 188 | 0.313 | 0.573 | 0.027 | 0.047 |
| rs17370311 | ENSG00000109654.10 | TRIM2 | 4_154515504_G_A_b37 | 442010 | 188 | 0.313 | 0.695 | 0.021 | 0.053 |
| rs17370311 | ENSG00000137460.4 | FHDC1 | 4_154515504_G_A_b37 | 658000 | 188 | 0.313 | 0.406 | -0.033 | 0.040 |
| rs17370311 | ENSG00000231841.1 | RP11-206F17.2 | 4_154515504_G_A_b37 | 666906 | 188 | 0.313 | 0.869 | -0.010 | 0.063 |
| rs17370311 | ENSG00000164144.10 | ARFIP1 | 4_154515504_G_A_b37 | 814415 | 188 | 0.313 | 0.075 | -0.059 | 0.033 |
| rs17370311 | ENSG00000170006.7 | TMEM154 | 4_154515504_G_A_b37 | 914187 | 188 | 0.313 | 0.969 | 0.001 | 0.018 |
| rs17370311 | ENSG00000248571.1 | RP11-768B22.2 | 4_154515504_G_A_b37 | 927984 | 188 | 0.313 | 0.414 | 0.050 | 0.061 |
| rs17370311 | ENSG00000240669.1 | RP11-555K12.2 | 4_154515504_G_A_b37 | 980019 | 188 | 0.313 | 0.717 | 0.024 | 0.067 |
| rs13141035 | ENSG00000171560.10 | FGA | 4_154515736_C_T_b37 | -996182 | 188 | 0.313 | 0.202 | 0.061 | 0.047 |
| rs13141035 | ENSG00000171564.7 | FGB | 4_154515736_C_T_b37 | -968372 | 188 | 0.313 | 0.479 | 0.031 | 0.044 |
| rs13141035 | ENSG00000171566.7 | PLRG1 | 4_154515736_C_T_b37 | -955851 | 188 | 0.313 | 0.151 | -0.034 | 0.024 |
| rs13141035 | ENSG00000145423.4 | SFRP2 | 4_154515736_C_T_b37 | -194536 | 188 | 0.313 | 0.098 | 0.077 | 0.047 |
| rs13141035 | ENSG00000145428.10 | RNF175 | 4_154515736_C_T_b37 | -165651 | 188 | 0.313 | 0.251 | -0.025 | 0.022 |
| rs13141035 | ENSG00000249309.1 | RP11-153M7.5 | 4_154515736_C_T_b37 | -125743 | 188 | 0.313 | 0.718 | -0.013 | 0.035 |
| rs13141035 | ENSG00000137462.6 | TLR2 | 4_154515736_C_T_b37 | -106916 | 188 | 0.313 | 0.274 | -0.023 | 0.021 |
| rs13141035 | ENSG00000248208.1 | RP11-153M7.1 | 4_154515736_C_T_b37 | -63988 | 188 | 0.313 | 0.422 | -0.045 | 0.055 |
| rs13141035 | ENSG00000250771.2 | RP11-153M7.3 | 4_154515736_C_T_b37 | -45584 | 188 | 0.313 | 0.477 | 0.036 | 0.050 |
| rs13141035 | ENSG00000121210.11 | KIAA0922 | 4_154515736_C_T_b37 | 128238 | 188 | 0.313 | 0.170 | -0.047 | 0.034 |
| rs13141035 | ENSG00000121211.3 | MND1 | 4_154515736_C_T_b37 | 249935 | 188 | 0.313 | 0.573 | 0.027 | 0.047 |
| rs13141035 | ENSG00000109654.10 | TRIM2 | 4_154515736_C_T_b37 | 442242 | 188 | 0.313 | 0.695 | 0.021 | 0.053 |
| rs13141035 | ENSG00000137460.4 | FHDC1 | 4_154515736_C_T_b37 | 658232 | 188 | 0.313 | 0.406 | -0.033 | 0.040 |
| rs13141035 | ENSG00000231841.1 | RP11-206F17.2 | 4_154515736_C_T_b37 | 667138 | 188 | 0.313 | 0.869 | -0.010 | 0.063 |
| rs13141035 | ENSG00000164144.10 | ARFIP1 | 4_154515736_C_T_b37 | 814647 | 188 | 0.313 | 0.075 | -0.059 | 0.033 |
| rs13141035 | ENSG00000170006.7 | TMEM154 | 4_154515736_C_T_b37 | 914419 | 188 | 0.313 | 0.969 | 0.001 | 0.018 |
| rs13141035 | ENSG00000248571.1 | RP11-768B22.2 | 4_154515736_C_T_b37 | 928216 | 188 | 0.313 | 0.414 | 0.050 | 0.061 |
| rs13141035 | ENSG00000240669.1 | RP11-555K12.2 | 4_154515736_C_T_b37 | 980251 | 188 | 0.313 | 0.717 | 0.024 | 0.067 |
| rs13141035 | ENSG00000171560.10 | FGA | 4_154515856_G_A_b37 | -996062 | 188 | 0.313 | 0.202 | 0.061 | 0.047 |
| rs13141035 | ENSG00000171564.7 | FGB | 4_154515856_G_A_b37 | -968252 | 188 | 0.313 | 0.479 | 0.031 | 0.044 |
| rs13141035 | ENSG00000171566.7 | PLRG1 | 4_154515856_G_A_b37 | -955731 | 188 | 0.313 | 0.151 | -0.034 | 0.024 |
| rs13141035 | ENSG00000145423.4 | SFRP2 | 4_154515856_G_A_b37 | -194416 | 188 | 0.313 | 0.098 | 0.077 | 0.047 |
| rs13141035 | ENSG00000145428.10 | RNF175 | 4_154515856_G_A_b37 | -165531 | 188 | 0.313 | 0.251 | -0.025 | 0.022 |
| rs13141035 | ENSG00000249309.1 | RP11-153M7.5 | 4_154515856_G_A_b37 | -125623 | 188 | 0.313 | 0.718 | -0.013 | 0.035 |
| rs13141035 | ENSG00000137462.6 | TLR2 | 4_154515856_G_A_b37 | -106796 | 188 | 0.313 | 0.274 | -0.023 | 0.021 |
| rs13141035 | ENSG00000248208.1 | RP11-153M7.1 | 4_154515856_G_A_b37 | -63868 | 188 | 0.313 | 0.422 | -0.045 | 0.055 |
| rs13141035 | ENSG00000250771.2 | RP11-153M7.3 | 4_154515856_G_A_b37 | -45464 | 188 | 0.313 | 0.477 | 0.036 | 0.050 |
| rs13141035 | ENSG00000121210.11 | KIAA0922 | 4_154515856_G_A_b37 | 128358 | 188 | 0.313 | 0.170 | -0.047 | 0.034 |
| rs13141035 | ENSG00000121211.3 | MND1 | 4_154515856_G_A_b37 | 250055 | 188 | 0.313 | 0.573 | 0.027 | 0.047 |
| rs13141035 | ENSG00000109654.10 | TRIM2 | 4_154515856_G_A_b37 | 442362 | 188 | 0.313 | 0.695 | 0.021 | 0.053 |
| rs13141035 | ENSG00000137460.4 | FHDC1 | 4_154515856_G_A_b37 | 658352 | 188 | 0.313 | 0.406 | -0.033 | 0.040 |
| rs13141035 | ENSG00000231841.1 | RP11-206F17.2 | 4_154515856_G_A_b37 | 667258 | 188 | 0.313 | 0.869 | -0.010 | 0.063 |
| rs13141035 | ENSG00000164144.10 | ARFIP1 | 4_154515856_G_A_b37 | 814767 | 188 | 0.313 | 0.075 | -0.059 | 0.033 |
| rs13141035 | ENSG00000170006.7 | TMEM154 | 4_154515856_G_A_b37 | 914539 | 188 | 0.313 | 0.969 | 0.001 | 0.018 |
| rs13141035 | ENSG00000248571.1 | RP11-768B22.2 | 4_154515856_G_A_b37 | 928336 | 188 | 0.313 | 0.414 | 0.050 | 0.061 |
| rs13141035 | ENSG00000240669.1 | RP11-555K12.2 | 4_154515856_G_A_b37 | 980371 | 188 | 0.313 | 0.717 | 0.024 | 0.067 |
| rs13146602 | ENSG00000171560.10 | FGA | 4_154515977_C_T_b37 | -995941 | 187 | 0.312 | 0.194 | 0.061 | 0.047 |
| rs13146602 | ENSG00000171564.7 | FGB | 4_154515977_C_T_b37 | -968131 | 187 | 0.312 | 0.459 | 0.032 | 0.044 |
| rs13146602 | ENSG00000171566.7 | PLRG1 | 4_154515977_C_T_b37 | -955610 | 187 | 0.312 | 0.161 | -0.033 | 0.024 |
| rs13146602 | ENSG00000145423.4 | SFRP2 | 4_154515977_C_T_b37 | -194295 | 187 | 0.312 | 0.103 | 0.076 | 0.046 |
| rs13146602 | ENSG00000145428.10 | RNF175 | 4_154515977_C_T_b37 | -165410 | 187 | 0.312 | 0.275 | -0.024 | 0.022 |
| rs13146602 | ENSG00000249309.1 | RP11-153M7.5 | 4_154515977_C_T_b37 | -125502 | 187 | 0.312 | 0.685 | -0.014 | 0.035 |
| rs13146602 | ENSG00000137462.6 | TLR2 | 4_154515977_C_T_b37 | -106675 | 187 | 0.312 | 0.300 | -0.022 | 0.021 |
| rs13146602 | ENSG00000248208.1 | RP11-153M7.1 | 4_154515977_C_T_b37 | -63747 | 187 | 0.312 | 0.470 | -0.040 | 0.055 |
| rs13146602 | ENSG00000250771.2 | RP11-153M7.3 | 4_154515977_C_T_b37 | -45343 | 187 | 0.312 | 0.503 | 0.034 | 0.050 |
| rs13146602 | ENSG00000121210.11 | KIAA0922 | 4_154515977_C_T_b37 | 128479 | 187 | 0.312 | 0.196 | -0.044 | 0.034 |
| rs13146602 | ENSG00000121211.3 | MND1 | 4_154515977_C_T_b37 | 250176 | 187 | 0.312 | 0.471 | 0.034 | 0.047 |
| rs13146602 | ENSG00000109654.10 | TRIM2 | 4_154515977_C_T_b37 | 442483 | 187 | 0.312 | 0.715 | 0.019 | 0.053 |
| rs13146602 | ENSG00000137460.4 | FHDC1 | 4_154515977_C_T_b37 | 658473 | 187 | 0.312 | 0.480 | -0.028 | 0.040 |
| rs13146602 | ENSG00000231841.1 | RP11-206F17.2 | 4_154515977_C_T_b37 | 667379 | 187 | 0.312 | 0.923 | -0.006 | 0.063 |
| rs13146602 | ENSG00000164144.10 | ARFIP1 | 4_154515977_C_T_b37 | 814888 | 187 | 0.312 | 0.082 | -0.058 | 0.033 |
| rs13146602 | ENSG00000170006.7 | TMEM154 | 4_154515977_C_T_b37 | 914660 | 187 | 0.312 | 0.977 | 0.001 | 0.018 |
| rs13146602 | ENSG00000248571.1 | RP11-768B22.2 | 4_154515977_C_T_b37 | 928457 | 187 | 0.312 | 0.436 | 0.047 | 0.061 |
| rs13146602 | ENSG00000240669.1 | RP11-555K12.2 | 4_154515977_C_T_b37 | 980492 | 187 | 0.312 | 0.642 | 0.031 | 0.067 |
| rs13147188 | ENSG00000171560.10 | FGA | 4_154516060_G_A_b37 | -995858 | 187 | 0.310 | 0.204 | 0.060 | 0.047 |
| rs13147188 | ENSG00000171564.7 | FGB | 4_154516060_G_A_b37 | -968048 | 187 | 0.310 | 0.446 | 0.033 | 0.044 |
| rs13147188 | ENSG00000171566.7 | PLRG1 | 4_154516060_G_A_b37 | -955527 | 187 | 0.310 | 0.166 | -0.033 | 0.024 |
| rs13147188 | ENSG00000145423.4 | SFRP2 | 4_154516060_G_A_b37 | -194212 | 187 | 0.310 | 0.101 | 0.077 | 0.047 |
| rs13147188 | ENSG00000145428.10 | RNF175 | 4_154516060_G_A_b37 | -165327 | 187 | 0.310 | 0.182 | -0.029 | 0.022 |
| rs13147188 | ENSG00000249309.1 | RP11-153M7.5 | 4_154516060_G_A_b37 | -125419 | 187 | 0.310 | 0.740 | -0.012 | 0.035 |
| rs13147188 | ENSG00000137462.6 | TLR2 | 4_154516060_G_A_b37 | -106592 | 187 | 0.310 | 0.243 | -0.025 | 0.021 |
| rs13147188 | ENSG00000248208.1 | RP11-153M7.1 | 4_154516060_G_A_b37 | -63664 | 187 | 0.310 | 0.343 | -0.053 | 0.056 |
| rs13147188 | ENSG00000250771.2 | RP11-153M7.3 | 4_154516060_G_A_b37 | -45260 | 187 | 0.310 | 0.455 | 0.037 | 0.050 |
| rs13147188 | ENSG00000121210.11 | KIAA0922 | 4_154516060_G_A_b37 | 128562 | 187 | 0.310 | 0.114 | -0.054 | 0.034 |
| rs13147188 | ENSG00000121211.3 | MND1 | 4_154516060_G_A_b37 | 250259 | 187 | 0.310 | 0.541 | 0.029 | 0.048 |
| rs13147188 | ENSG00000109654.10 | TRIM2 | 4_154516060_G_A_b37 | 442566 | 187 | 0.310 | 0.747 | 0.017 | 0.053 |
| rs13147188 | ENSG00000137460.4 | FHDC1 | 4_154516060_G_A_b37 | 658556 | 187 | 0.310 | 0.557 | -0.024 | 0.040 |
| rs13147188 | ENSG00000231841.1 | RP11-206F17.2 | 4_154516060_G_A_b37 | 667462 | 187 | 0.310 | 0.961 | -0.003 | 0.063 |
| rs13147188 | ENSG00000164144.10 | ARFIP1 | 4_154516060_G_A_b37 | 814971 | 187 | 0.310 | 0.087 | -0.057 | 0.033 |
| rs13147188 | ENSG00000170006.7 | TMEM154 | 4_154516060_G_A_b37 | 914743 | 187 | 0.310 | 0.932 | 0.002 | 0.018 |
| rs13147188 | ENSG00000248571.1 | RP11-768B22.2 | 4_154516060_G_A_b37 | 928540 | 187 | 0.310 | 0.378 | 0.054 | 0.061 |
| rs13147188 | ENSG00000240669.1 | RP11-555K12.2 | 4_154516060_G_A_b37 | 980575 | 187 | 0.310 | 0.676 | 0.028 | 0.067 |
| rs13147224 | ENSG00000171560.10 | FGA | 4_154516121_G_A_b37 | -995797 | 188 | 0.313 | 0.202 | 0.061 | 0.047 |
| rs13147224 | ENSG00000171564.7 | FGB | 4_154516121_G_A_b37 | -967987 | 188 | 0.313 | 0.479 | 0.031 | 0.044 |
| rs13147224 | ENSG00000171566.7 | PLRG1 | 4_154516121_G_A_b37 | -955466 | 188 | 0.313 | 0.151 | -0.034 | 0.024 |
| rs13147224 | ENSG00000145423.4 | SFRP2 | 4_154516121_G_A_b37 | -194151 | 188 | 0.313 | 0.098 | 0.077 | 0.047 |
| rs13147224 | ENSG00000145428.10 | RNF175 | 4_154516121_G_A_b37 | -165266 | 188 | 0.313 | 0.251 | -0.025 | 0.022 |
| rs13147224 | ENSG00000249309.1 | RP11-153M7.5 | 4_154516121_G_A_b37 | -125358 | 188 | 0.313 | 0.718 | -0.013 | 0.035 |
| rs13147224 | ENSG00000137462.6 | TLR2 | 4_154516121_G_A_b37 | -106531 | 188 | 0.313 | 0.274 | -0.023 | 0.021 |
| rs13147224 | ENSG00000248208.1 | RP11-153M7.1 | 4_154516121_G_A_b37 | -63603 | 188 | 0.313 | 0.422 | -0.045 | 0.055 |
| rs13147224 | ENSG00000250771.2 | RP11-153M7.3 | 4_154516121_G_A_b37 | -45199 | 188 | 0.313 | 0.477 | 0.036 | 0.050 |
| rs13147224 | ENSG00000121210.11 | KIAA0922 | 4_154516121_G_A_b37 | 128623 | 188 | 0.313 | 0.170 | -0.047 | 0.034 |
| rs13147224 | ENSG00000121211.3 | MND1 | 4_154516121_G_A_b37 | 250320 | 188 | 0.313 | 0.573 | 0.027 | 0.047 |
| rs13147224 | ENSG00000109654.10 | TRIM2 | 4_154516121_G_A_b37 | 442627 | 188 | 0.313 | 0.695 | 0.021 | 0.053 |
| rs13147224 | ENSG00000137460.4 | FHDC1 | 4_154516121_G_A_b37 | 658617 | 188 | 0.313 | 0.406 | -0.033 | 0.040 |
| rs13147224 | ENSG00000231841.1 | RP11-206F17.2 | 4_154516121_G_A_b37 | 667523 | 188 | 0.313 | 0.869 | -0.010 | 0.063 |
| rs13147224 | ENSG00000164144.10 | ARFIP1 | 4_154516121_G_A_b37 | 815032 | 188 | 0.313 | 0.075 | -0.059 | 0.033 |
| rs13147224 | ENSG00000170006.7 | TMEM154 | 4_154516121_G_A_b37 | 914804 | 188 | 0.313 | 0.969 | 0.001 | 0.018 |
| rs13147224 | ENSG00000248571.1 | RP11-768B22.2 | 4_154516121_G_A_b37 | 928601 | 188 | 0.313 | 0.414 | 0.050 | 0.061 |
| rs13147224 | ENSG00000240669.1 | RP11-555K12.2 | 4_154516121_G_A_b37 | 980636 | 188 | 0.313 | 0.717 | 0.024 | 0.067 |
| rs7690279 | ENSG00000171560.10 | FGA | 4_154517215_A_G_b37 | -994703 | 189 | 0.316 | 0.196 | 0.061 | 0.047 |
| rs7690279 | ENSG00000171564.7 | FGB | 4_154517215_A_G_b37 | -966893 | 189 | 0.316 | 0.470 | 0.031 | 0.044 |
| rs7690279 | ENSG00000171566.7 | PLRG1 | 4_154517215_A_G_b37 | -954372 | 189 | 0.316 | 0.124 | -0.036 | 0.024 |
| rs7690279 | ENSG00000145423.4 | SFRP2 | 4_154517215_A_G_b37 | -193057 | 189 | 0.316 | 0.155 | 0.066 | 0.046 |
| rs7690279 | ENSG00000145428.10 | RNF175 | 4_154517215_A_G_b37 | -164172 | 189 | 0.316 | 0.189 | -0.029 | 0.022 |
| rs7690279 | ENSG00000249309.1 | RP11-153M7.5 | 4_154517215_A_G_b37 | -124264 | 189 | 0.316 | 0.619 | -0.017 | 0.035 |
| rs7690279 | ENSG00000137462.6 | TLR2 | 4_154517215_A_G_b37 | -105437 | 189 | 0.316 | 0.249 | -0.024 | 0.021 |
| rs7690279 | ENSG00000248208.1 | RP11-153M7.1 | 4_154517215_A_G_b37 | -62509 | 189 | 0.316 | 0.509 | -0.036 | 0.055 |
| rs7690279 | ENSG00000250771.2 | RP11-153M7.3 | 4_154517215_A_G_b37 | -44105 | 189 | 0.316 | 0.500 | 0.034 | 0.050 |
| rs7690279 | ENSG00000121210.11 | KIAA0922 | 4_154517215_A_G_b37 | 129717 | 189 | 0.316 | 0.219 | -0.042 | 0.034 |
| rs7690279 | ENSG00000121211.3 | MND1 | 4_154517215_A_G_b37 | 251414 | 189 | 0.316 | 0.528 | 0.030 | 0.047 |
| rs7690279 | ENSG00000109654.10 | TRIM2 | 4_154517215_A_G_b37 | 443721 | 189 | 0.316 | 0.577 | 0.029 | 0.053 |
| rs7690279 | ENSG00000137460.4 | FHDC1 | 4_154517215_A_G_b37 | 659711 | 189 | 0.316 | 0.506 | -0.026 | 0.040 |
| rs7690279 | ENSG00000231841.1 | RP11-206F17.2 | 4_154517215_A_G_b37 | 668617 | 189 | 0.316 | 0.799 | -0.016 | 0.063 |
| rs7690279 | ENSG00000164144.10 | ARFIP1 | 4_154517215_A_G_b37 | 816126 | 189 | 0.316 | 0.079 | -0.058 | 0.033 |
| rs7690279 | ENSG00000170006.7 | TMEM154 | 4_154517215_A_G_b37 | 915898 | 189 | 0.316 | 0.995 | 0.000 | 0.018 |
| rs7690279 | ENSG00000248571.1 | RP11-768B22.2 | 4_154517215_A_G_b37 | 929695 | 189 | 0.316 | 0.418 | 0.049 | 0.061 |
| rs7690279 | ENSG00000240669.1 | RP11-555K12.2 | 4_154517215_A_G_b37 | 981730 | 189 | 0.316 | 0.708 | 0.025 | 0.067 |
| rs7690932 | ENSG00000171560.10 | FGA | 4_154517294_G_T_b37 | -994624 | 189 | 0.316 | 0.196 | 0.061 | 0.047 |
| rs7690932 | ENSG00000171564.7 | FGB | 4_154517294_G_T_b37 | -966814 | 189 | 0.316 | 0.470 | 0.031 | 0.044 |
| rs7690932 | ENSG00000171566.7 | PLRG1 | 4_154517294_G_T_b37 | -954293 | 189 | 0.316 | 0.124 | -0.036 | 0.024 |
| rs7690932 | ENSG00000145423.4 | SFRP2 | 4_154517294_G_T_b37 | -192978 | 189 | 0.316 | 0.155 | 0.066 | 0.046 |
| rs7690932 | ENSG00000145428.10 | RNF175 | 4_154517294_G_T_b37 | -164093 | 189 | 0.316 | 0.189 | -0.029 | 0.022 |
| rs7690932 | ENSG00000249309.1 | RP11-153M7.5 | 4_154517294_G_T_b37 | -124185 | 189 | 0.316 | 0.619 | -0.017 | 0.035 |
| rs7690932 | ENSG00000137462.6 | TLR2 | 4_154517294_G_T_b37 | -105358 | 189 | 0.316 | 0.249 | -0.024 | 0.021 |
| rs7690932 | ENSG00000248208.1 | RP11-153M7.1 | 4_154517294_G_T_b37 | -62430 | 189 | 0.316 | 0.509 | -0.036 | 0.055 |
| rs7690932 | ENSG00000250771.2 | RP11-153M7.3 | 4_154517294_G_T_b37 | -44026 | 189 | 0.316 | 0.500 | 0.034 | 0.050 |
| rs7690932 | ENSG00000121210.11 | KIAA0922 | 4_154517294_G_T_b37 | 129796 | 189 | 0.316 | 0.219 | -0.042 | 0.034 |
| rs7690932 | ENSG00000121211.3 | MND1 | 4_154517294_G_T_b37 | 251493 | 189 | 0.316 | 0.528 | 0.030 | 0.047 |
| rs7690932 | ENSG00000109654.10 | TRIM2 | 4_154517294_G_T_b37 | 443800 | 189 | 0.316 | 0.577 | 0.029 | 0.053 |
| rs7690932 | ENSG00000137460.4 | FHDC1 | 4_154517294_G_T_b37 | 659790 | 189 | 0.316 | 0.506 | -0.026 | 0.040 |
| rs7690932 | ENSG00000231841.1 | RP11-206F17.2 | 4_154517294_G_T_b37 | 668696 | 189 | 0.316 | 0.799 | -0.016 | 0.063 |
| rs7690932 | ENSG00000164144.10 | ARFIP1 | 4_154517294_G_T_b37 | 816205 | 189 | 0.316 | 0.079 | -0.058 | 0.033 |
| rs7690932 | ENSG00000170006.7 | TMEM154 | 4_154517294_G_T_b37 | 915977 | 189 | 0.316 | 0.995 | 0.000 | 0.018 |
| rs7690932 | ENSG00000248571.1 | RP11-768B22.2 | 4_154517294_G_T_b37 | 929774 | 189 | 0.316 | 0.418 | 0.049 | 0.061 |
| rs7690932 | ENSG00000240669.1 | RP11-555K12.2 | 4_154517294_G_T_b37 | 981809 | 189 | 0.316 | 0.708 | 0.025 | 0.067 |
| rs7696772 | ENSG00000171560.10 | FGA | 4_154518029_G_A_b37 | -993889 | 188 | 0.315 | 0.185 | 0.063 | 0.047 |
| rs7696772 | ENSG00000171564.7 | FGB | 4_154518029_G_A_b37 | -966079 | 188 | 0.315 | 0.466 | 0.032 | 0.044 |
| rs7696772 | ENSG00000171566.7 | PLRG1 | 4_154518029_G_A_b37 | -953558 | 188 | 0.315 | 0.122 | -0.036 | 0.024 |
| rs7696772 | ENSG00000145423.4 | SFRP2 | 4_154518029_G_A_b37 | -192243 | 188 | 0.315 | 0.154 | 0.066 | 0.046 |
| rs7696772 | ENSG00000145428.10 | RNF175 | 4_154518029_G_A_b37 | -163358 | 188 | 0.315 | 0.187 | -0.029 | 0.022 |
| rs7696772 | ENSG00000249309.1 | RP11-153M7.5 | 4_154518029_G_A_b37 | -123450 | 188 | 0.315 | 0.627 | -0.017 | 0.035 |
| rs7696772 | ENSG00000137462.6 | TLR2 | 4_154518029_G_A_b37 | -104623 | 188 | 0.315 | 0.250 | -0.024 | 0.021 |
| rs7696772 | ENSG00000248208.1 | RP11-153M7.1 | 4_154518029_G_A_b37 | -61695 | 188 | 0.315 | 0.522 | -0.035 | 0.055 |
| rs7696772 | ENSG00000250771.2 | RP11-153M7.3 | 4_154518029_G_A_b37 | -43291 | 188 | 0.315 | 0.474 | 0.036 | 0.050 |
| rs7696772 | ENSG00000121210.11 | KIAA0922 | 4_154518029_G_A_b37 | 130531 | 188 | 0.315 | 0.225 | -0.041 | 0.034 |
| rs7696772 | ENSG00000121211.3 | MND1 | 4_154518029_G_A_b37 | 252228 | 188 | 0.315 | 0.514 | 0.031 | 0.047 |
| rs7696772 | ENSG00000109654.10 | TRIM2 | 4_154518029_G_A_b37 | 444535 | 188 | 0.315 | 0.585 | 0.029 | 0.053 |
| rs7696772 | ENSG00000137460.4 | FHDC1 | 4_154518029_G_A_b37 | 660525 | 188 | 0.315 | 0.481 | -0.028 | 0.040 |
| rs7696772 | ENSG00000231841.1 | RP11-206F17.2 | 4_154518029_G_A_b37 | 669431 | 188 | 0.315 | 0.798 | -0.016 | 0.063 |
| rs7696772 | ENSG00000164144.10 | ARFIP1 | 4_154518029_G_A_b37 | 816940 | 188 | 0.315 | 0.068 | -0.060 | 0.033 |
| rs7696772 | ENSG00000170006.7 | TMEM154 | 4_154518029_G_A_b37 | 916712 | 188 | 0.315 | 0.947 | 0.001 | 0.018 |
| rs7696772 | ENSG00000248571.1 | RP11-768B22.2 | 4_154518029_G_A_b37 | 930509 | 188 | 0.315 | 0.418 | 0.049 | 0.061 |
| rs7696772 | ENSG00000240669.1 | RP11-555K12.2 | 4_154518029_G_A_b37 | 982544 | 188 | 0.315 | 0.733 | 0.023 | 0.067 |
| rs7655793 | ENSG00000171560.10 | FGA | 4_154518580_C_T_b37 | -993338 | 187 | 0.315 | 0.190 | 0.062 | 0.047 |
| rs7655793 | ENSG00000171564.7 | FGB | 4_154518580_C_T_b37 | -965528 | 187 | 0.315 | 0.475 | 0.031 | 0.044 |
| rs7655793 | ENSG00000171566.7 | PLRG1 | 4_154518580_C_T_b37 | -953007 | 187 | 0.315 | 0.114 | -0.037 | 0.024 |
| rs7655793 | ENSG00000145423.4 | SFRP2 | 4_154518580_C_T_b37 | -191692 | 187 | 0.315 | 0.151 | 0.067 | 0.046 |
| rs7655793 | ENSG00000145428.10 | RNF175 | 4_154518580_C_T_b37 | -162807 | 187 | 0.315 | 0.190 | -0.028 | 0.022 |
| rs7655793 | ENSG00000249309.1 | RP11-153M7.5 | 4_154518580_C_T_b37 | -122899 | 187 | 0.315 | 0.621 | -0.017 | 0.035 |
| rs7655793 | ENSG00000137462.6 | TLR2 | 4_154518580_C_T_b37 | -104072 | 187 | 0.315 | 0.252 | -0.024 | 0.021 |
| rs7655793 | ENSG00000248208.1 | RP11-153M7.1 | 4_154518580_C_T_b37 | -61144 | 187 | 0.315 | 0.535 | -0.034 | 0.055 |
| rs7655793 | ENSG00000250771.2 | RP11-153M7.3 | 4_154518580_C_T_b37 | -42740 | 187 | 0.315 | 0.518 | 0.032 | 0.050 |
| rs7655793 | ENSG00000121210.11 | KIAA0922 | 4_154518580_C_T_b37 | 131082 | 187 | 0.315 | 0.201 | -0.044 | 0.034 |
| rs7655793 | ENSG00000121211.3 | MND1 | 4_154518580_C_T_b37 | 252779 | 187 | 0.315 | 0.502 | 0.032 | 0.047 |
| rs7655793 | ENSG00000109654.10 | TRIM2 | 4_154518580_C_T_b37 | 445086 | 187 | 0.315 | 0.534 | 0.033 | 0.053 |
| rs7655793 | ENSG00000137460.4 | FHDC1 | 4_154518580_C_T_b37 | 661076 | 187 | 0.315 | 0.513 | -0.026 | 0.040 |
| rs7655793 | ENSG00000231841.1 | RP11-206F17.2 | 4_154518580_C_T_b37 | 669982 | 187 | 0.315 | 0.828 | -0.014 | 0.063 |
| rs7655793 | ENSG00000164144.10 | ARFIP1 | 4_154518580_C_T_b37 | 817491 | 187 | 0.315 | 0.088 | -0.057 | 0.033 |
| rs7655793 | ENSG00000170006.7 | TMEM154 | 4_154518580_C_T_b37 | 917263 | 187 | 0.315 | 0.984 | 0.000 | 0.018 |
| rs7655793 | ENSG00000248571.1 | RP11-768B22.2 | 4_154518580_C_T_b37 | 931060 | 187 | 0.315 | 0.430 | 0.048 | 0.061 |
| rs7655793 | ENSG00000240669.1 | RP11-555K12.2 | 4_154518580_C_T_b37 | 983095 | 187 | 0.315 | 0.689 | 0.027 | 0.067 |
| rs7656141 | ENSG00000171560.10 | FGA | 4_154518625_G_T_b37 | -993293 | 187 | 0.316 | 0.227 | 0.057 | 0.047 |
| rs7656141 | ENSG00000171564.7 | FGB | 4_154518625_G_T_b37 | -965483 | 187 | 0.316 | 0.513 | 0.029 | 0.044 |
| rs7656141 | ENSG00000171566.7 | PLRG1 | 4_154518625_G_T_b37 | -952962 | 187 | 0.316 | 0.115 | -0.037 | 0.024 |
| rs7656141 | ENSG00000145423.4 | SFRP2 | 4_154518625_G_T_b37 | -191647 | 187 | 0.316 | 0.150 | 0.067 | 0.047 |
| rs7656141 | ENSG00000145428.10 | RNF175 | 4_154518625_G_T_b37 | -162762 | 187 | 0.316 | 0.186 | -0.029 | 0.022 |
| rs7656141 | ENSG00000249309.1 | RP11-153M7.5 | 4_154518625_G_T_b37 | -122854 | 187 | 0.316 | 0.641 | -0.016 | 0.035 |
| rs7656141 | ENSG00000137462.6 | TLR2 | 4_154518625_G_T_b37 | -104027 | 187 | 0.316 | 0.241 | -0.025 | 0.021 |
| rs7656141 | ENSG00000248208.1 | RP11-153M7.1 | 4_154518625_G_T_b37 | -61099 | 187 | 0.316 | 0.496 | -0.038 | 0.056 |
| rs7656141 | ENSG00000250771.2 | RP11-153M7.3 | 4_154518625_G_T_b37 | -42695 | 187 | 0.316 | 0.535 | 0.031 | 0.050 |
| rs7656141 | ENSG00000121210.11 | KIAA0922 | 4_154518625_G_T_b37 | 131127 | 187 | 0.316 | 0.225 | -0.042 | 0.034 |
| rs7656141 | ENSG00000121211.3 | MND1 | 4_154518625_G_T_b37 | 252824 | 187 | 0.316 | 0.425 | 0.038 | 0.048 |
| rs7656141 | ENSG00000109654.10 | TRIM2 | 4_154518625_G_T_b37 | 445131 | 187 | 0.316 | 0.634 | 0.025 | 0.053 |
| rs7656141 | ENSG00000137460.4 | FHDC1 | 4_154518625_G_T_b37 | 661121 | 187 | 0.316 | 0.525 | -0.025 | 0.040 |
| rs7656141 | ENSG00000231841.1 | RP11-206F17.2 | 4_154518625_G_T_b37 | 670027 | 187 | 0.316 | 0.846 | -0.012 | 0.063 |
| rs7656141 | ENSG00000164144.10 | ARFIP1 | 4_154518625_G_T_b37 | 817536 | 187 | 0.316 | 0.090 | -0.056 | 0.033 |
| rs7656141 | ENSG00000170006.7 | TMEM154 | 4_154518625_G_T_b37 | 917308 | 187 | 0.316 | 0.985 | 0.000 | 0.018 |
| rs7656141 | ENSG00000248571.1 | RP11-768B22.2 | 4_154518625_G_T_b37 | 931105 | 187 | 0.316 | 0.425 | 0.049 | 0.061 |
| rs7656141 | ENSG00000240669.1 | RP11-555K12.2 | 4_154518625_G_T_b37 | 983140 | 187 | 0.316 | 0.708 | 0.025 | 0.067 |
| rs11732127 | ENSG00000171560.10 | FGA | 4_154519002_G_A_b37 | -992916 | 189 | 0.316 | 0.196 | 0.061 | 0.047 |
| rs11732127 | ENSG00000171564.7 | FGB | 4_154519002_G_A_b37 | -965106 | 189 | 0.316 | 0.470 | 0.031 | 0.044 |
| rs11732127 | ENSG00000171566.7 | PLRG1 | 4_154519002_G_A_b37 | -952585 | 189 | 0.316 | 0.124 | -0.036 | 0.024 |
| rs11732127 | ENSG00000145423.4 | SFRP2 | 4_154519002_G_A_b37 | -191270 | 189 | 0.316 | 0.155 | 0.066 | 0.046 |
| rs11732127 | ENSG00000145428.10 | RNF175 | 4_154519002_G_A_b37 | -162385 | 189 | 0.316 | 0.189 | -0.029 | 0.022 |
| rs11732127 | ENSG00000249309.1 | RP11-153M7.5 | 4_154519002_G_A_b37 | -122477 | 189 | 0.316 | 0.619 | -0.017 | 0.035 |
| rs11732127 | ENSG00000137462.6 | TLR2 | 4_154519002_G_A_b37 | -103650 | 189 | 0.316 | 0.249 | -0.024 | 0.021 |
| rs11732127 | ENSG00000248208.1 | RP11-153M7.1 | 4_154519002_G_A_b37 | -60722 | 189 | 0.316 | 0.509 | -0.036 | 0.055 |
| rs11732127 | ENSG00000250771.2 | RP11-153M7.3 | 4_154519002_G_A_b37 | -42318 | 189 | 0.316 | 0.500 | 0.034 | 0.050 |
| rs11732127 | ENSG00000121210.11 | KIAA0922 | 4_154519002_G_A_b37 | 131504 | 189 | 0.316 | 0.219 | -0.042 | 0.034 |
| rs11732127 | ENSG00000121211.3 | MND1 | 4_154519002_G_A_b37 | 253201 | 189 | 0.316 | 0.528 | 0.030 | 0.047 |
| rs11732127 | ENSG00000109654.10 | TRIM2 | 4_154519002_G_A_b37 | 445508 | 189 | 0.316 | 0.577 | 0.029 | 0.053 |
| rs11732127 | ENSG00000137460.4 | FHDC1 | 4_154519002_G_A_b37 | 661498 | 189 | 0.316 | 0.506 | -0.026 | 0.040 |
| rs11732127 | ENSG00000231841.1 | RP11-206F17.2 | 4_154519002_G_A_b37 | 670404 | 189 | 0.316 | 0.799 | -0.016 | 0.063 |
| rs11732127 | ENSG00000164144.10 | ARFIP1 | 4_154519002_G_A_b37 | 817913 | 189 | 0.316 | 0.079 | -0.058 | 0.033 |
| rs11732127 | ENSG00000170006.7 | TMEM154 | 4_154519002_G_A_b37 | 917685 | 189 | 0.316 | 0.995 | 0.000 | 0.018 |
| rs11732127 | ENSG00000248571.1 | RP11-768B22.2 | 4_154519002_G_A_b37 | 931482 | 189 | 0.316 | 0.418 | 0.049 | 0.061 |
| rs11732127 | ENSG00000240669.1 | RP11-555K12.2 | 4_154519002_G_A_b37 | 983517 | 189 | 0.316 | 0.708 | 0.025 | 0.067 |
| rs11943070 | ENSG00000171560.10 | FGA | 4_154520418_G_A_b37 | -991500 | 190 | 0.316 | 0.204 | 0.060 | 0.047 |
| rs11943070 | ENSG00000171564.7 | FGB | 4_154520418_G_A_b37 | -963690 | 190 | 0.316 | 0.493 | 0.030 | 0.044 |
| rs11943070 | ENSG00000171566.7 | PLRG1 | 4_154520418_G_A_b37 | -951169 | 190 | 0.316 | 0.088 | -0.040 | 0.024 |
| rs11943070 | ENSG00000145423.4 | SFRP2 | 4_154520418_G_A_b37 | -189854 | 190 | 0.316 | 0.182 | 0.062 | 0.047 |
| rs11943070 | ENSG00000145428.10 | RNF175 | 4_154520418_G_A_b37 | -160969 | 190 | 0.316 | 0.233 | -0.026 | 0.022 |
| rs11943070 | ENSG00000249309.1 | RP11-153M7.5 | 4_154520418_G_A_b37 | -121061 | 190 | 0.316 | 0.762 | -0.011 | 0.035 |
| rs11943070 | ENSG00000137462.6 | TLR2 | 4_154520418_G_A_b37 | -102234 | 190 | 0.316 | 0.273 | -0.023 | 0.021 |
| rs11943070 | ENSG00000248208.1 | RP11-153M7.1 | 4_154520418_G_A_b37 | -59306 | 190 | 0.316 | 0.530 | -0.035 | 0.056 |
| rs11943070 | ENSG00000250771.2 | RP11-153M7.3 | 4_154520418_G_A_b37 | -40902 | 190 | 0.316 | 0.550 | 0.030 | 0.050 |
| rs11943070 | ENSG00000121210.11 | KIAA0922 | 4_154520418_G_A_b37 | 132920 | 190 | 0.316 | 0.196 | -0.044 | 0.034 |
| rs11943070 | ENSG00000121211.3 | MND1 | 4_154520418_G_A_b37 | 254617 | 190 | 0.316 | 0.540 | 0.029 | 0.047 |
| rs11943070 | ENSG00000109654.10 | TRIM2 | 4_154520418_G_A_b37 | 446924 | 190 | 0.316 | 0.550 | 0.032 | 0.053 |
| rs11943070 | ENSG00000137460.4 | FHDC1 | 4_154520418_G_A_b37 | 662914 | 190 | 0.316 | 0.472 | -0.029 | 0.040 |
| rs11943070 | ENSG00000231841.1 | RP11-206F17.2 | 4_154520418_G_A_b37 | 671820 | 190 | 0.316 | 0.750 | -0.020 | 0.063 |
| rs11943070 | ENSG00000164144.10 | ARFIP1 | 4_154520418_G_A_b37 | 819329 | 190 | 0.316 | 0.078 | -0.059 | 0.033 |
| rs11943070 | ENSG00000170006.7 | TMEM154 | 4_154520418_G_A_b37 | 919101 | 190 | 0.316 | 0.951 | 0.001 | 0.018 |
| rs11943070 | ENSG00000248571.1 | RP11-768B22.2 | 4_154520418_G_A_b37 | 932898 | 190 | 0.316 | 0.359 | 0.056 | 0.061 |
| rs11943070 | ENSG00000240669.1 | RP11-555K12.2 | 4_154520418_G_A_b37 | 984933 | 190 | 0.316 | 0.701 | 0.026 | 0.067 |
| rs10000932 | ENSG00000171560.10 | FGA | 4_154520561_A_G_b37 | -991357 | 206 | 0.348 | 0.372 | 0.041 | 0.046 |
| rs10000932 | ENSG00000171564.7 | FGB | 4_154520561_A_G_b37 | -963547 | 206 | 0.348 | 0.594 | 0.023 | 0.043 |
| rs10000932 | ENSG00000171566.7 | PLRG1 | 4_154520561_A_G_b37 | -951026 | 206 | 0.348 | 0.171 | -0.032 | 0.023 |
| rs10000932 | ENSG00000145423.4 | SFRP2 | 4_154520561_A_G_b37 | -189711 | 206 | 0.348 | 0.192 | 0.059 | 0.045 |
| rs10000932 | ENSG00000145428.10 | RNF175 | 4_154520561_A_G_b37 | -160826 | 206 | 0.348 | 0.039 | -0.044 | 0.021 |
| rs10000932 | ENSG00000249309.1 | RP11-153M7.5 | 4_154520561_A_G_b37 | -120918 | 206 | 0.348 | 0.481 | -0.024 | 0.034 |
| rs10000932 | ENSG00000137462.6 | TLR2 | 4_154520561_A_G_b37 | -102091 | 206 | 0.348 | 0.189 | -0.027 | 0.020 |
| rs10000932 | ENSG00000248208.1 | RP11-153M7.1 | 4_154520561_A_G_b37 | -59163 | 206 | 0.348 | 0.633 | -0.026 | 0.054 |
| rs10000932 | ENSG00000250771.2 | RP11-153M7.3 | 4_154520561_A_G_b37 | -40759 | 206 | 0.348 | 0.708 | 0.018 | 0.049 |
| rs10000932 | ENSG00000121210.11 | KIAA0922 | 4_154520561_A_G_b37 | 133063 | 206 | 0.348 | 0.284 | -0.036 | 0.033 |
| rs10000932 | ENSG00000121211.3 | MND1 | 4_154520561_A_G_b37 | 254760 | 206 | 0.348 | 0.695 | 0.018 | 0.046 |
| rs10000932 | ENSG00000109654.10 | TRIM2 | 4_154520561_A_G_b37 | 447067 | 206 | 0.348 | 0.915 | 0.005 | 0.051 |
| rs10000932 | ENSG00000137460.4 | FHDC1 | 4_154520561_A_G_b37 | 663057 | 206 | 0.348 | 0.209 | -0.049 | 0.039 |
| rs10000932 | ENSG00000231841.1 | RP11-206F17.2 | 4_154520561_A_G_b37 | 671963 | 206 | 0.348 | 0.502 | -0.041 | 0.061 |
| rs10000932 | ENSG00000164144.10 | ARFIP1 | 4_154520561_A_G_b37 | 819472 | 206 | 0.348 | 0.032 | -0.069 | 0.032 |
| rs10000932 | ENSG00000170006.7 | TMEM154 | 4_154520561_A_G_b37 | 919244 | 206 | 0.348 | 0.614 | 0.009 | 0.018 |
| rs10000932 | ENSG00000248571.1 | RP11-768B22.2 | 4_154520561_A_G_b37 | 933041 | 206 | 0.348 | 0.161 | 0.083 | 0.059 |
| rs10000932 | ENSG00000240669.1 | RP11-555K12.2 | 4_154520561_A_G_b37 | 985076 | 206 | 0.348 | 0.901 | -0.008 | 0.065 |
| rs11734584 | ENSG00000171560.10 | FGA | 4_154521309_G_A_b37 | -990609 | 182 | 0.302 | 0.182 | 0.064 | 0.048 |
| rs11734584 | ENSG00000171564.7 | FGB | 4_154521309_G_A_b37 | -962799 | 182 | 0.302 | 0.374 | 0.039 | 0.044 |
| rs11734584 | ENSG00000171566.7 | PLRG1 | 4_154521309_G_A_b37 | -950278 | 182 | 0.302 | 0.041 | -0.049 | 0.024 |
| rs11734584 | ENSG00000145423.4 | SFRP2 | 4_154521309_G_A_b37 | -188963 | 182 | 0.302 | 0.301 | 0.049 | 0.047 |
| rs11734584 | ENSG00000145428.10 | RNF175 | 4_154521309_G_A_b37 | -160078 | 182 | 0.302 | 0.117 | -0.035 | 0.022 |
| rs11734584 | ENSG00000249309.1 | RP11-153M7.5 | 4_154521309_G_A_b37 | -120170 | 182 | 0.302 | 0.666 | -0.015 | 0.035 |
| rs11734584 | ENSG00000137462.6 | TLR2 | 4_154521309_G_A_b37 | -101343 | 182 | 0.302 | 0.320 | -0.021 | 0.021 |
| rs11734584 | ENSG00000248208.1 | RP11-153M7.1 | 4_154521309_G_A_b37 | -58415 | 182 | 0.302 | 0.997 | 0.000 | 0.056 |
| rs11726361 | ENSG00000250771.2 | RP11-153M7.3 | 4_154521309_G_A_b37 | -40011 | 182 | 0.302 | 0.477 | 0.036 | 0.051 |
| rs11734584 | ENSG00000121210.11 | KIAA0922 | 4_154521309_G_A_b37 | 133811 | 182 | 0.302 | 0.205 | -0.044 | 0.035 |
| rs11734584 | ENSG00000121211.3 | MND1 | 4_154521309_G_A_b37 | 255508 | 182 | 0.302 | 0.468 | 0.035 | 0.048 |
| rs11734584 | ENSG00000109654.10 | TRIM2 | 4_154521309_G_A_b37 | 447815 | 182 | 0.302 | 0.487 | 0.037 | 0.053 |
| rs11734584 | ENSG00000137460.4 | FHDC1 | 4_154521309_G_A_b37 | 663805 | 182 | 0.302 | 0.194 | -0.052 | 0.040 |
| rs11734584 | ENSG00000231841.1 | RP11-206F17.2 | 4_154521309_G_A_b37 | 672711 | 182 | 0.302 | 0.870 | 0.010 | 0.064 |
| rs11734584 | ENSG00000164144.10 | ARFIP1 | 4_154521309_G_A_b37 | 820220 | 182 | 0.302 | 0.089 | -0.057 | 0.033 |
| rs11734584 | ENSG00000170006.7 | TMEM154 | 4_154521309_G_A_b37 | 919992 | 182 | 0.302 | 0.597 | -0.010 | 0.018 |
| rs11734584 | ENSG00000248571.1 | RP11-768B22.2 | 4_154521309_G_A_b37 | 933789 | 182 | 0.302 | 0.529 | 0.039 | 0.062 |
| rs11734584 | ENSG00000240669.1 | RP11-555K12.2 | 4_154521309_G_A_b37 | 985824 | 182 | 0.302 | 0.966 | -0.003 | 0.068 |
| rs11726361 | ENSG00000171560.10 | FGA | 4_154521489_A_C_b37 | -990429 | 172 | 0.291 | 0.415 | 0.040 | 0.049 |
| rs11726361 | ENSG00000171564.7 | FGB | 4_154521489_A_C_b37 | -962619 | 172 | 0.291 | 0.798 | 0.012 | 0.046 |
| rs11726361 | ENSG00000171566.7 | PLRG1 | 4_154521489_A_C_b37 | -950098 | 172 | 0.291 | 0.084 | -0.043 | 0.025 |
| rs11726361 | ENSG00000145423.4 | SFRP2 | 4_154521489_A_C_b37 | -188783 | 172 | 0.291 | 0.461 | 0.036 | 0.049 |
| rs11726361 | ENSG00000145428.10 | RNF175 | 4_154521489_A_C_b37 | -159898 | 172 | 0.291 | 0.169 | -0.031 | 0.023 |
| rs11726361 | ENSG00000249309.1 | RP11-153M7.5 | 4_154521489_A_C_b37 | -119990 | 172 | 0.291 | 0.626 | -0.018 | 0.036 |
| rs11726361 | ENSG00000137462.6 | TLR2 | 4_154521489_A_C_b37 | -101163 | 172 | 0.291 | 0.304 | -0.022 | 0.022 |
| rs11726361 | ENSG00000248208.1 | RP11-153M7.1 | 4_154521489_A_C_b37 | -58235 | 172 | 0.291 | 0.725 | -0.020 | 0.058 |
| rs11726361 | ENSG00000250771.2 | RP11-153M7.3 | 4_154521489_A_C_b37 | -39831 | 172 | 0.291 | 0.620 | 0.026 | 0.052 |
| rs11726361 | ENSG00000121210.11 | KIAA0922 | 4_154521489_A_C_b37 | 133991 | 172 | 0.291 | 0.116 | -0.056 | 0.036 |
| rs11726361 | ENSG00000121211.3 | MND1 | 4_154521489_A_C_b37 | 255688 | 172 | 0.291 | 0.445 | 0.038 | 0.049 |
| rs11726361 | ENSG00000109654.10 | TRIM2 | 4_154521489_A_C_b37 | 447995 | 172 | 0.291 | 0.381 | 0.048 | 0.055 |
| rs11726361 | ENSG00000137460.4 | FHDC1 | 4_154521489_A_C_b37 | 663985 | 172 | 0.291 | 0.268 | -0.046 | 0.041 |
| rs11726361 | ENSG00000231841.1 | RP11-206F17.2 | 4_154521489_A_C_b37 | 672891 | 172 | 0.291 | 0.751 | 0.021 | 0.066 |
| rs11726361 | ENSG00000164144.10 | ARFIP1 | 4_154521489_A_C_b37 | 820400 | 172 | 0.291 | 0.091 | -0.059 | 0.034 |
| rs11726361 | ENSG00000170006.7 | TMEM154 | 4_154521489_A_C_b37 | 920172 | 172 | 0.291 | 0.473 | -0.014 | 0.019 |
| rs11726361 | ENSG00000248571.1 | RP11-768B22.2 | 4_154521489_A_C_b37 | 933969 | 172 | 0.291 | 0.601 | 0.033 | 0.063 |
| rs11726361 | ENSG00000240669.1 | RP11-555K12.2 | 4_154521489_A_C_b37 | 986004 | 172 | 0.291 | 0.892 | -0.009 | 0.070 |
| rs11734684 | ENSG00000171560.10 | FGA | 4_154521574_G_A_b37 | -990344 | 200 | 0.348 | 0.409 | 0.039 | 0.047 |
| rs11734684 | ENSG00000171564.7 | FGB | 4_154521574_G_A_b37 | -962534 | 200 | 0.348 | 0.884 | 0.006 | 0.043 |
| rs11734684 | ENSG00000171566.7 | PLRG1 | 4_154521574_G_A_b37 | -950013 | 200 | 0.348 | 0.051 | -0.046 | 0.023 |
| rs11734684 | ENSG00000145423.4 | SFRP2 | 4_154521574_G_A_b37 | -188698 | 200 | 0.348 | 0.215 | 0.057 | 0.046 |
| rs11734684 | ENSG00000145428.10 | RNF175 | 4_154521574_G_A_b37 | -159813 | 200 | 0.348 | 0.206 | -0.027 | 0.022 |
| rs11734684 | ENSG00000249309.1 | RP11-153M7.5 | 4_154521574_G_A_b37 | -119905 | 200 | 0.348 | 0.914 | 0.004 | 0.034 |
| rs11734684 | ENSG00000137462.6 | TLR2 | 4_154521574_G_A_b37 | -101078 | 200 | 0.348 | 0.630 | -0.010 | 0.021 |
| rs11734684 | ENSG00000248208.1 | RP11-153M7.1 | 4_154521574_G_A_b37 | -58150 | 200 | 0.348 | 0.639 | -0.026 | 0.055 |
| rs11734684 | ENSG00000250771.2 | RP11-153M7.3 | 4_154521574_G_A_b37 | -39746 | 200 | 0.348 | 0.699 | 0.019 | 0.049 |
| rs11734684 | ENSG00000121210.11 | KIAA0922 | 4_154521574_G_A_b37 | 134076 | 200 | 0.348 | 0.345 | -0.032 | 0.034 |
| rs11734684 | ENSG00000121211.3 | MND1 | 4_154521574_G_A_b37 | 255773 | 200 | 0.348 | 0.933 | 0.004 | 0.047 |
| rs11734684 | ENSG00000109654.10 | TRIM2 | 4_154521574_G_A_b37 | 448080 | 200 | 0.348 | 0.275 | 0.057 | 0.052 |
| rs11734684 | ENSG00000137460.4 | FHDC1 | 4_154521574_G_A_b37 | 664070 | 200 | 0.348 | 0.172 | -0.054 | 0.039 |
| rs11734684 | ENSG00000231841.1 | RP11-206F17.2 | 4_154521574_G_A_b37 | 672976 | 200 | 0.348 | 0.661 | -0.027 | 0.062 |
| rs11734684 | ENSG00000164144.10 | ARFIP1 | 4_154521574_G_A_b37 | 820485 | 200 | 0.348 | 0.072 | -0.059 | 0.033 |
| rs11734684 | ENSG00000170006.7 | TMEM154 | 4_154521574_G_A_b37 | 920257 | 200 | 0.348 | 0.667 | -0.008 | 0.018 |
| rs11734684 | ENSG00000248571.1 | RP11-768B22.2 | 4_154521574_G_A_b37 | 934054 | 200 | 0.348 | 0.626 | 0.029 | 0.060 |
| rs11734684 | ENSG00000240669.1 | RP11-555K12.2 | 4_154521574_G_A_b37 | 986089 | 200 | 0.348 | 0.860 | -0.012 | 0.066 |
| rs11099889 | ENSG00000171560.10 | FGA | 4_154521663_G_A_b37 | -990255 | 183 | 0.302 | 0.178 | 0.065 | 0.048 |
| rs11099889 | ENSG00000171564.7 | FGB | 4_154521663_G_A_b37 | -962445 | 183 | 0.302 | 0.370 | 0.040 | 0.044 |
| rs11099889 | ENSG00000171566.7 | PLRG1 | 4_154521663_G_A_b37 | -949924 | 183 | 0.302 | 0.041 | -0.049 | 0.024 |
| rs11099889 | ENSG00000145423.4 | SFRP2 | 4_154521663_G_A_b37 | -188609 | 183 | 0.302 | 0.290 | 0.050 | 0.047 |
| rs11099889 | ENSG00000145428.10 | RNF175 | 4_154521663_G_A_b37 | -159724 | 183 | 0.302 | 0.129 | -0.034 | 0.022 |
| rs11099889 | ENSG00000249309.1 | RP11-153M7.5 | 4_154521663_G_A_b37 | -119816 | 183 | 0.302 | 0.614 | -0.018 | 0.035 |
| rs11099889 | ENSG00000137462.6 | TLR2 | 4_154521663_G_A_b37 | -100989 | 183 | 0.302 | 0.335 | -0.020 | 0.021 |
| rs11099889 | ENSG00000248208.1 | RP11-153M7.1 | 4_154521663_G_A_b37 | -58061 | 183 | 0.302 | 0.957 | -0.003 | 0.056 |
| rs11099889 | ENSG00000250771.2 | RP11-153M7.3 | 4_154521663_G_A_b37 | -39657 | 183 | 0.302 | 0.523 | 0.032 | 0.051 |
| rs11099889 | ENSG00000121210.11 | KIAA0922 | 4_154521663_G_A_b37 | 134165 | 183 | 0.302 | 0.208 | -0.044 | 0.035 |
| rs11099889 | ENSG00000121211.3 | MND1 | 4_154521663_G_A_b37 | 255862 | 183 | 0.302 | 0.465 | 0.035 | 0.048 |
| rs11099889 | ENSG00000109654.10 | TRIM2 | 4_154521663_G_A_b37 | 448169 | 183 | 0.302 | 0.464 | 0.039 | 0.053 |
| rs11099889 | ENSG00000137460.4 | FHDC1 | 4_154521663_G_A_b37 | 664159 | 183 | 0.302 | 0.186 | -0.053 | 0.040 |
| rs11099889 | ENSG00000231841.1 | RP11-206F17.2 | 4_154521663_G_A_b37 | 673065 | 183 | 0.302 | 0.881 | 0.010 | 0.064 |
| rs11099889 | ENSG00000164144.10 | ARFIP1 | 4_154521663_G_A_b37 | 820574 | 183 | 0.302 | 0.084 | -0.058 | 0.033 |
| rs11099889 | ENSG00000170006.7 | TMEM154 | 4_154521663_G_A_b37 | 920346 | 183 | 0.302 | 0.628 | -0.009 | 0.018 |
| rs11099889 | ENSG00000248571.1 | RP11-768B22.2 | 4_154521663_G_A_b37 | 934143 | 183 | 0.302 | 0.550 | 0.037 | 0.062 |
| rs11099889 | ENSG00000240669.1 | RP11-555K12.2 | 4_154521663_G_A_b37 | 986178 | 183 | 0.302 | 0.960 | -0.003 | 0.068 |
| rs3891364 | ENSG00000171560.10 | FGA | 4_154521998_T_C_b37 | -989920 | 206 | 0.347 | 0.254 | 0.053 | 0.046 |
| rs3891364 | ENSG00000171564.7 | FGB | 4_154521998_T_C_b37 | -962110 | 206 | 0.347 | 0.525 | 0.027 | 0.043 |
| rs3891364 | ENSG00000171566.7 | PLRG1 | 4_154521998_T_C_b37 | -949589 | 206 | 0.347 | 0.074 | -0.041 | 0.023 |
| rs3891364 | ENSG00000145423.4 | SFRP2 | 4_154521998_T_C_b37 | -188274 | 206 | 0.347 | 0.319 | 0.046 | 0.046 |
| rs3891364 | ENSG00000145428.10 | RNF175 | 4_154521998_T_C_b37 | -159389 | 206 | 0.347 | 0.187 | -0.028 | 0.021 |
| rs3891364 | ENSG00000249309.1 | RP11-153M7.5 | 4_154521998_T_C_b37 | -119481 | 206 | 0.347 | 0.826 | -0.008 | 0.034 |
| rs3891364 | ENSG00000137462.6 | TLR2 | 4_154521998_T_C_b37 | -100654 | 206 | 0.347 | 0.462 | -0.015 | 0.021 |
| rs3891364 | ENSG00000248208.1 | RP11-153M7.1 | 4_154521998_T_C_b37 | -57726 | 206 | 0.347 | 0.995 | 0.000 | 0.054 |
| rs3891364 | ENSG00000250771.2 | RP11-153M7.3 | 4_154521998_T_C_b37 | -39322 | 206 | 0.347 | 0.689 | 0.020 | 0.049 |
| rs3891364 | ENSG00000121210.11 | KIAA0922 | 4_154521998_T_C_b37 | 134500 | 206 | 0.347 | 0.478 | -0.024 | 0.034 |
| rs3891364 | ENSG00000121211.3 | MND1 | 4_154521998_T_C_b37 | 256197 | 206 | 0.347 | 0.826 | 0.010 | 0.046 |
| rs3891364 | ENSG00000109654.10 | TRIM2 | 4_154521998_T_C_b37 | 448504 | 206 | 0.347 | 0.442 | 0.040 | 0.052 |
| rs3891364 | ENSG00000137460.4 | FHDC1 | 4_154521998_T_C_b37 | 664494 | 206 | 0.347 | 0.093 | -0.065 | 0.039 |
| rs3891364 | ENSG00000231841.1 | RP11-206F17.2 | 4_154521998_T_C_b37 | 673400 | 206 | 0.347 | 0.857 | -0.011 | 0.062 |
| rs3891364 | ENSG00000164144.10 | ARFIP1 | 4_154521998_T_C_b37 | 820909 | 206 | 0.347 | 0.028 | -0.071 | 0.032 |
| rs3891364 | ENSG00000170006.7 | TMEM154 | 4_154521998_T_C_b37 | 920681 | 206 | 0.347 | 0.804 | 0.004 | 0.018 |
| rs3891364 | ENSG00000248571.1 | RP11-768B22.2 | 4_154521998_T_C_b37 | 934478 | 206 | 0.347 | 0.649 | 0.027 | 0.060 |
| rs3891364 | ENSG00000240669.1 | RP11-555K12.2 | 4_154521998_T_C_b37 | 986513 | 206 | 0.347 | 0.900 | 0.008 | 0.065 |

Supplementary Material

# Supplementary Table

**Supplementary Table 3.**

We identified eQTL values between SNPs and gene expression provided by v7 GTEx whole blood data (https://storage.googleapis.com/gtex_analysis_v7/single_tissue_eqtl_data/GTEx_Analysis_v7_eQTL_all_associations.tar.gz) to find genes affected by allele types of SNPs associated with cold sensitivity. This result shows only the relationship between significant SNP and gene expression on the same chromosome (cis-eQTL). SP1 (ENSG00000185591.5) was the only case where q-value (test measures the proportion of false positives incurred when that particular test is called significant) was less than 0.05.
