# Supplementary material for: Regulation of CIRP by genetic factors of SP1 related to cold sensitivity
Source: Front Immunol. 2022 Sep 16;13:994699. doi: 10.3389/fimmu.2022.994699 (PMC9524288; doi:10.3389/fimmu.2022.994699)
Supplement: Supplementary file 7 [file Table_4.docx]

Supplementary 4. LD-based clumping results in cold-sensitivity associated SNPs.

| CHR | SNP | BP | P | Block Start | Block End | KB | NSNPS | SNPS |
| --- | --- | --- | --- | --- | --- | --- | --- | --- |
| 4 | rs2685468 | 81989503 | 7.92E-07 | 81984196 | 82003787 | 19.592 | 7 | rs2643925\|rs7436092\|rs2643921\|rs7695260\|rs2685468\|4-82003702\|rs17005046 |
| 4 | rs11943070 | 154520418 | 2.55E-06 | 154510843 | 154525458 | 14.616 | 47 | rs10517574\|rs36068308\|rs34076653\|rs62324755\|rs17279148\|rs1371160\|rs7664062\|rs62324756\|rs1371158\|rs7669418\|rs7669741\|rs893628\|rs7670485\|rs7670353\|rs28386897\|rs138978734\|rs13109887\|rs17279217\|rs17370297\|rs17370311\|rs13141035\|rs13141748\|rs13146602\|rs13147188\|rs13147224\|rs13147431\|rs1371156\|rs7690279\|rs7690932\|rs7696772\|rs7697047\|rs7655793\|rs7656141\|rs11732127\|rs13118469\|rs13118949\|rs13145743\|rs11943070\|rs10000932\|rs9990596\|rs9654222\|rs11734584\|rs11726361\|rs11734684\|rs11099889\|rs3891364\|rs1063151 |
| 5 | rs4291038 | 164688851 | 6.44E-06 | 164685326 | 164726566 | 41.241 | 65 | rs1541913\|rs4291038\|rs17074343\|AX-247296079\|rs971569\|rs12519802\|rs35075132\|rs973845\|rs973847\|rs7714945\|rs7732563\|rs12109827\|rs1897578\|rs13153940\|5-164693951\|rs7442642\|rs11135362\|rs12186829\|rs6870836\|rs17063630\|rs1432994\|rs1160830\|rs17419172\|rs1158559\|rs11749582\|rs17074571\|rs1432998\|rs17074579\|rs2011999\|rs17074593\|rs10056972\|rs718903\|rs6896656\|rs17074610\|rs1432999\|rs6859347\|rs1368418\|rs1368419\|rs1368420\|rs4868925\|rs1433000\|rs1428502\|rs1433001\|rs6556815\|rs1991803\|rs6860354\|rs1368421\|rs13180364\|rs10434705\|rs1368422\|rs1594672\|rs11135363\|rs7734762\|rs7721635\|rs62383664\|5-164721271\|rs12523003\|AX-14964622\|rs13190045\|rs17074826\|rs12153272\|rs34961943\|rs1145648\|rs62383667\|rs2861141 |
| 7 | rs148142477 | 43714469 | 8.25E-06 |  |  |  |  |  |
| 10 | rs821955 | 108456522 | 1.40E-07 | 108369320 | 108417488 | 48.169 | 59 | rs6584757\|rs12772601\|rs7095267\|rs7099866\|rs7099641\|rs911574\|rs10884337\|rs1022745\|rs7085383\|rs4918241\|rs1322005\|rs7074484\|rs7899072\|rs11192980\|rs11192981\|rs7068505\|rs8181286\|rs7073704\|rs10748920\|rs7901090\|10-108393045\|10-108393116\|rs1535397\|rs1535396\|rs4918244\|rs4918245\|rs7922320\|rs7906440\|rs7906750\|rs7910603\|rs7087196\|rs12220921\|rs11192986\|rs11598968\|rs7905341\|AX-14690042\|rs11192988\|rs8181404\|rs11192989\|rs7099241\|rs12221205\|rs12219036\|rs7088196\|rs1322017\|rs1322014\|rs11192994\|rs9732511\|rs11192996\|rs1407283\|rs11192997\|rs11192998\|rs11192999\|rs17121117\|rs11193000\|rs11193001\|rs7084276\|10-108416737\|rs56663696\|rs11193002 |
| 10 | rs11192989 | 108403873 | 3.11E-06 | 108421956 | 108484515 | 62.56 | 66 | rs11598223\|rs6584759\|rs11193007\|rs11193008\|rs12412558\|rs11598147\|rs11193010\|rs2475258\|rs821933\|rs821932\|rs821931\|rs821930\|rs821929\|rs821928\|rs821927\|rs821926\|rs821925\|rs821943\|rs821942\|rs12256633\|rs821940\|rs821939\|rs821937\|rs11193012\|rs821936\|rs821935\|rs1252035\|rs821934\|rs1252036\|rs821961\|rs821959\|rs821958\|rs821957\|rs821956\|rs821955\|rs17121228\|rs17121230\|rs703495\|rs703496\|rs821951\|rs7096521\|rs821950\|rs1923325\|rs12240368\|rs1322012\|rs2756231\|rs911580\|rs911579\|rs2756232\|rs11193018\|rs821954\|rs821953\|rs821952\|rs1272085\|rs1252034\|rs821949\|rs821948\|rs821947\|rs821946\|rs821944\|rs2486149\|rs2756233\|rs2756234\|rs1964864\|rs1040871\|rs1358874 |
| 12 | rs7962345 | 53762887 | 6.13E-07 | 53684619 | 53813402 | 128.784 | 52 | rs61754164\|rs79211945\|12-53707106\|rs10747666\|AX-17000366\|rs10783573\|rs3847780\|rs11829355\|rs10876434\|rs11170509\|rs11170510\|rs57355676\|12-53751033\|rs11170516\|rs35437931\|rs12582170\|rs12828860\|rs76958490\|rs58123204\|rs7962345\|rs10876447\|rs7131938\|rs36065378\|rs3741651\|rs10876449\|rs11170525\|rs12368491\|rs7315782\|rs2694847\|rs7134665\|12-53787866\|rs2460882\|12-53788286\|rs11170532\|12-53789763\|rs2608302\|rs7300593\|rs7133236\|12-53792914\|rs35969688\|rs2947336\|12-53794288\|rs57676448\|rs2947337\|12-53798768\|rs784882\|12-53801461\|rs7968637\|rs12817984\|rs10876450\|12-53811667\|rs17098950 |
| 15 | rs6576563 | 26578079 | 6.34E-07 | 26564189 | 26594649 | 30.461 | 72 | 15-26564189\|15-26564493\|15-26564789\|15-26565377\|rs34526896\|15-26566444\|rs12902317\|rs12903035\|rs55899970\|15-26569794\|rs7173305\|rs7173159\|rs7178189\|rs12907868\|rs6576559\|rs12908892\|rs10873625\|15-26571841\|rs12912966\|rs4523900\|rs11853274\|rs11855604\|rs7162659\|rs7169552\|15-26573058\|15-26573203\|rs12442453\|rs7171237\|15-26575621\|rs12907174\|rs9920736\|rs4528540\|rs34823996\|rs6576561\|rs6576562\|rs6576563\|rs6576564\|rs7168118\|rs7168485\|15-26578574\|15-26578803\|15-26579088\|rs12914178\|rs4365262\|rs8039457\|rs8041307\|15-26580968\|rs12899924\|rs12900779\|rs12717759\|rs12904865\|rs12906538\|rs7175299\|rs7177522\|rs6576565\|rs6576566\|rs8038626\|rs10444893\|rs5004573\|rs5004574\|15-26587334\|rs139414406\|rs34633148\|AX-11206337\|15-26590161\|15-26591413\|15-26591537\|15-26591856\|15-26592617\|15-26593818\|15-26594135\|rs12591807 |
| 20 | rs2294966 | 42834504 | 4.94E-06 | 42834253 | 42844189 | 9.937 | 22 | rs57634151\|rs6017287\|rs2294966\|rs6017288\|rs6130568\|rs62204525\|rs2232286\|rs2232282\|rs6017289\|rs1007125\|rs956609\|rs2235807\|rs2235806\|20-42838209\|rs2143606\|rs4142441\|rs6031458\|rs8115598\|20-42842363\|20-42843271\|20-42843929\|rs62204530 |

Supplementary Material

# Supplementary Table

**Supplementary Table 4.**

The clumping procedure takes all SNPs that are significant at threshold *p*< 1*10-5, they form a clump of all other SNPs within a 250kb kb distance from the index SNPs. The SNPS field lists all SNPs bundled with an index SNP, regardless of the *p*-value of that SNP. The Block Start and Block End fields indicate the start and end physical positions of the chromosomes on the LD block. The KB field indicates the size of the LD block to which the index SNP belongs. The SNPs field indicated number of SNPs on the LD block.
