# Supplementary material for: Regulation of CIRP by genetic factors of SP1 related to cold sensitivity
Source: Front Immunol. 2022 Sep 16;13:994699. doi: 10.3389/fimmu.2022.994699 (PMC9524288; doi:10.3389/fimmu.2022.994699)
Supplement: Supplementary file 8 [file Table_5.docx]

Supplementary Material

# Supplementary Table5. Pairwise linkage disequilibrium expressed as the squared correlation coefficient (*r^2^*) between the single-nucleotide polymorphisms (SNPs) genotyped in the chr12:53684619-53813402 region.

| CHR_A | BP_A | SNP_A | CHR_B | BP_B | SNP_B | *r^2^* |
| --- | --- | --- | --- | --- | --- | --- |
| 12 | 53684619 | rs61754164 | 12 | 53692222 | rs79211945 | 0.989359 |
| 12 | 53684619 | rs61754164 | 12 | 53707106 | 12-53707106 | 0.987601 |
| 12 | 53684619 | rs61754164 | 12 | 53716836 | rs10747666 | 1 |
| 12 | 53684619 | rs61754164 | 12 | 53727955 | AX-17000366 | 0.952167 |
| 12 | 53684619 | rs61754164 | 12 | 53728369 | rs10783573 | 0.953554 |
| 12 | 53692222 | rs79211945 | 12 | 53707106 | 12-53707106 | 0.995419 |
| 12 | 53692222 | rs79211945 | 12 | 53716836 | rs10747666 | 0.990914 |
| 12 | 53692222 | rs79211945 | 12 | 53727955 | AX-17000366 | 0.92471 |
| 12 | 53692222 | rs79211945 | 12 | 53728369 | rs10783573 | 0.937775 |
| 12 | 53707106 | 12-53707106 | 12 | 53716836 | rs10747666 | 0.989281 |
| 12 | 53707106 | 12-53707106 | 12 | 53727955 | AX-17000366 | 0.941204 |
| 12 | 53707106 | 12-53707106 | 12 | 53728369 | rs10783573 | 0.955246 |
| 12 | 53707106 | 12-53707106 | 12 | 53731336 | rs3847780 | 0.773453 |
| 12 | 53707106 | 12-53707106 | 12 | 53733283 | rs11829355 | 0.797817 |
| 12 | 53716836 | rs10747666 | 12 | 53727955 | AX-17000366 | 0.976129 |
| 12 | 53716836 | rs10747666 | 12 | 53728369 | rs10783573 | 0.973816 |
| 12 | 53716836 | rs10747666 | 12 | 53731336 | rs3847780 | 0.755724 |
| 12 | 53716836 | rs10747666 | 12 | 53733283 | rs11829355 | 0.754993 |
| 12 | 53725559 | rs118172385 | 12 | 53734026 | 12-53734026 | 0.955187 |
| 12 | 53727955 | AX-17000366 | 12 | 53728369 | rs10783573 | 0.998467 |
| 12 | 53727955 | AX-17000366 | 12 | 53731336 | rs3847780 | 0.771576 |
| 12 | 53727955 | AX-17000366 | 12 | 53733283 | rs11829355 | 0.762818 |
| 12 | 53727955 | AX-17000366 | 12 | 53734675 | rs10876434 | 0.742114 |
| 12 | 53727955 | AX-17000366 | 12 | 53743050 | rs11170509 | 0.727094 |
| 12 | 53727955 | AX-17000366 | 12 | 53743734 | rs11170510 | 0.727194 |
| 12 | 53728369 | rs10783573 | 12 | 53731336 | rs3847780 | 0.804171 |
| 12 | 53728369 | rs10783573 | 12 | 53733283 | rs11829355 | 0.795595 |
| 12 | 53728369 | rs10783573 | 12 | 53734675 | rs10876434 | 0.773769 |
| 12 | 53728369 | rs10783573 | 12 | 53743050 | rs11170509 | 0.769608 |
| 12 | 53728369 | rs10783573 | 12 | 53743734 | rs11170510 | 0.769694 |
| 12 | 53728369 | rs10783573 | 12 | 53746487 | rs57355676 | 0.914648 |
| 12 | 53731336 | rs3847780 | 12 | 53733283 | rs11829355 | 0.991926 |
| 12 | 53731336 | rs3847780 | 12 | 53734675 | rs10876434 | 0.973296 |
| 12 | 53731336 | rs3847780 | 12 | 53743050 | rs11170509 | 0.956635 |
| 12 | 53731336 | rs3847780 | 12 | 53743734 | rs11170510 | 0.95665 |
| 12 | 53731336 | rs3847780 | 12 | 53746487 | rs57355676 | 0.772507 |
| 12 | 53731336 | rs3847780 | 12 | 53751033 | 12-53751033 | 0.711474 |
| 12 | 53731336 | rs3847780 | 12 | 53752692 | rs11170516 | 0.940158 |
| 12 | 53733283 | rs11829355 | 12 | 53734675 | rs10876434 | 0.987878 |
| 12 | 53733283 | rs11829355 | 12 | 53743050 | rs11170509 | 0.986539 |
| 12 | 53733283 | rs11829355 | 12 | 53743734 | rs11170510 | 0.986543 |
| 12 | 53733283 | rs11829355 | 12 | 53746487 | rs57355676 | 0.795618 |
| 12 | 53733283 | rs11829355 | 12 | 53751033 | 12-53751033 | 0.745328 |
| 12 | 53733283 | rs11829355 | 12 | 53752692 | rs11170516 | 0.985204 |
| 12 | 53733283 | rs11829355 | 12 | 53756354 | rs35437931 | 0.986534 |
| 12 | 53734675 | rs10876434 | 12 | 53743050 | rs11170509 | 1 |
| 12 | 53734675 | rs10876434 | 12 | 53743734 | rs11170510 | 1 |
| 12 | 53734675 | rs10876434 | 12 | 53746487 | rs57355676 | 0.804896 |
| 12 | 53734675 | rs10876434 | 12 | 53751033 | 12-53751033 | 0.753876 |
| 12 | 53734675 | rs10876434 | 12 | 53752692 | rs11170516 | 0.998634 |
| 12 | 53734675 | rs10876434 | 12 | 53756354 | rs35437931 | 1 |
| 12 | 53734675 | rs10876434 | 12 | 53757831 | rs12582170 | 0.997267 |
| 12 | 53734675 | rs10876434 | 12 | 53759803 | rs12828860 | 0.995902 |
| 12 | 53743050 | rs11170509 | 12 | 53743734 | rs11170510 | 1 |
| 12 | 53743050 | rs11170509 | 12 | 53746487 | rs57355676 | 0.806622 |
| 12 | 53743050 | rs11170509 | 12 | 53751033 | 12-53751033 | 0.755705 |
| 12 | 53743050 | rs11170509 | 12 | 53752692 | rs11170516 | 0.998639 |
| 12 | 53743050 | rs11170509 | 12 | 53756354 | rs35437931 | 1 |
| 12 | 53743050 | rs11170509 | 12 | 53757831 | rs12582170 | 0.997277 |
| 12 | 53743050 | rs11170509 | 12 | 53759803 | rs12828860 | 0.995918 |
| 12 | 53743050 | rs11170509 | 12 | 53759858 | rs76958490 | 0.802959 |
| **12** | **53743734** | **rs11170510** | **12** | **53746487** | **rs57355676** | **0.805569** |
| **12** | **53743734** | **rs11170510** | **12** | **53751033** | **12-53751033** | **0.754676** |
| **12** | **53743734** | **rs11170510** | **12** | **53752692** | **rs11170516** | **0.998639** |
| **12** | **53743734** | **rs11170510** | **12** | **53756354** | **rs35437931** | **1** |
| **12** | **53743734** | **rs11170510** | **12** | **53757831** | **rs12582170** | **0.997278** |
| **12** | **53743734** | **rs11170510** | **12** | **53759803** | **rs12828860** | **0.995919** |
| **12** | **53743734** | **rs11170510** | **12** | **53759858** | **rs76958490** | **0.80191** |
| **12** | **53743734** | **rs11170510** | **12** | **53760162** | **rs58123204** | **0.994077** |
| 12 | 53746487 | rs57355676 | 12 | 53751033 | 12-53751033 | 0.946527 |
| 12 | 53746487 | rs57355676 | 12 | 53752692 | rs11170516 | 0.803643 |
| 12 | 53746487 | rs57355676 | 12 | 53756354 | rs35437931 | 0.805451 |
| 12 | 53746487 | rs57355676 | 12 | 53757831 | rs12582170 | 0.802959 |
| 12 | 53746487 | rs57355676 | 12 | 53759803 | rs12828860 | 0.801453 |
| 12 | 53746487 | rs57355676 | 12 | 53759858 | rs76958490 | 0.995343 |
| 12 | 53746487 | rs57355676 | 12 | 53760162 | rs58123204 | 0.786269 |
| 12 | 53746487 | rs57355676 | 12 | 53762887 | rs7962345 | 0.798271 |
| 12 | 53751033 | 12-53751033 | 12 | 53752692 | rs11170516 | 0.753279 |
| 12 | 53751033 | 12-53751033 | 12 | 53756354 | rs35437931 | 0.755307 |
| 12 | 53751033 | 12-53751033 | 12 | 53757831 | rs12582170 | 0.752753 |
| 12 | 53751033 | 12-53751033 | 12 | 53759803 | rs12828860 | 0.751133 |
| 12 | 53751033 | 12-53751033 | 12 | 53759858 | rs76958490 | 0.940256 |
| 12 | 53751033 | 12-53751033 | 12 | 53760162 | rs58123204 | 0.731925 |
| 12 | 53751033 | 12-53751033 | 12 | 53762887 | rs7962345 | 0.7467 |
| 12 | 53751033 | 12-53751033 | 12 | 53770941 | rs10876447 | 0.751345 |
| 12 | 53752692 | rs11170516 | 12 | 53756354 | rs35437931 | 0.998643 |
| 12 | 53752692 | rs11170516 | 12 | 53757831 | rs12582170 | 0.994579 |
| 12 | 53752692 | rs11170516 | 12 | 53759803 | rs12828860 | 0.994576 |
| 12 | 53752692 | rs11170516 | 12 | 53759858 | rs76958490 | 0.800672 |
| 12 | 53752692 | rs11170516 | 12 | 53760162 | rs58123204 | 0.992617 |
| 12 | 53752692 | rs11170516 | 12 | 53762887 | rs7962345 | 0.994312 |
| 12 | 53752692 | rs11170516 | 12 | 53770941 | rs10876447 | 0.994459 |
| 12 | 53752692 | rs11170516 | 12 | 53772927 | rs7131938 | 0.994286 |
| 12 | 53756354 | rs35437931 | 12 | 53757831 | rs12582170 | 1 |
| 12 | 53756354 | rs35437931 | 12 | 53759803 | rs12828860 | 0.998643 |
| 12 | 53756354 | rs35437931 | 12 | 53759858 | rs76958490 | 0.805021 |
| 12 | 53756354 | rs35437931 | 12 | 53760162 | rs58123204 | 0.997047 |
| 12 | 53756354 | rs35437931 | 12 | 53762887 | rs7962345 | 0.998577 |
| 12 | 53756354 | rs35437931 | 12 | 53770941 | rs10876447 | 0.998614 |
| 12 | 53756354 | rs35437931 | 12 | 53772927 | rs7131938 | 0.998571 |
| 12 | 53756354 | rs35437931 | 12 | 53774779 | rs36065378 | 0.998488 |
| 12 | 53756354 | rs35437931 | 12 | 53777171 | rs3741651 | 0.998571 |
| 12 | 53757831 | rs12582170 | 12 | 53759803 | rs12828860 | 0.997292 |
| 12 | 53757831 | rs12582170 | 12 | 53759858 | rs76958490 | 0.804092 |
| 12 | 53757831 | rs12582170 | 12 | 53760162 | rs58123204 | 0.995579 |
| 12 | 53757831 | rs12582170 | 12 | 53762887 | rs7962345 | 0.99716 |
| 12 | 53757831 | rs12582170 | 12 | 53770941 | rs10876447 | 0.997233 |
| 12 | 53757831 | rs12582170 | 12 | 53772927 | rs7131938 | 0.997147 |
| 12 | 53757831 | rs12582170 | 12 | 53774779 | rs36065378 | 0.996982 |
| 12 | 53757831 | rs12582170 | 12 | 53777171 | rs3741651 | 0.997147 |
| 12 | 53759803 | rs12828860 | 12 | 53759858 | rs76958490 | 0.806286 |
| 12 | 53759803 | rs12828860 | 12 | 53760162 | rs58123204 | 0.998524 |
| 12 | 53759803 | rs12828860 | 12 | 53762887 | rs7962345 | 1 |
| 12 | 53759803 | rs12828860 | 12 | 53770941 | rs10876447 | 1 |
| 12 | 53759803 | rs12828860 | 12 | 53772927 | rs7131938 | 1 |
| 12 | 53759803 | rs12828860 | 12 | 53774779 | rs36065378 | 1 |
| 12 | 53759803 | rs12828860 | 12 | 53777171 | rs3741651 | 1 |
| 12 | 53759803 | rs12828860 | 12 | 53779775 | rs10876449 | 0.997146 |
| 12 | 53759858 | rs76958490 | 12 | 53760162 | rs58123204 | 0.791585 |
| 12 | 53759858 | rs76958490 | 12 | 53762887 | rs7962345 | 0.803185 |
| 12 | 53759858 | rs76958490 | 12 | 53770941 | rs10876447 | 0.806796 |
| 12 | 53759858 | rs76958490 | 12 | 53772927 | rs7131938 | 0.832213 |
| 12 | 53759858 | rs76958490 | 12 | 53774779 | rs36065378 | 0.830847 |
| 12 | 53759858 | rs76958490 | 12 | 53777171 | rs3741651 | 0.832213 |
| 12 | 53759858 | rs76958490 | 12 | 53779775 | rs10876449 | 0.829006 |
| 12 | 53759858 | rs76958490 | 12 | 53782628 | rs11170525 | 0.832147 |
| **12** | **53760162** | **rs58123204** | **12** | **53762887** | **rs7962345** | **0.998469** |
| **12** | **53760162** | **rs58123204** | **12** | **53770941** | **rs10876447** | **0.998487** |
| **12** | **53760162** | **rs58123204** | **12** | **53772927** | **rs7131938** | **0.998437** |
| **12** | **53760162** | **rs58123204** | **12** | **53774779** | **rs36065378** | **0.998339** |
| **12** | **53760162** | **rs58123204** | **12** | **53777171** | **rs3741651** | **0.998437** |
| **12** | **53760162** | **rs58123204** | **12** | **53779775** | **rs10876449** | **0.995311** |
| **12** | **53760162** | **rs58123204** | **12** | **53782628** | **rs11170525** | **0.998437** |
| **12** | **53760162** | **rs58123204** | **12** | **53782959** | **rs12368491** | **0.998437** |
| 12 | 53762887 | rs7962345 | 12 | 53770941 | rs10876447 | 1 |
| 12 | 53762887 | rs7962345 | 12 | 53772927 | rs7131938 | 1 |
| 12 | 53762887 | rs7962345 | 12 | 53774779 | rs36065378 | 1 |
| 12 | 53762887 | rs7962345 | 12 | 53777171 | rs3741651 | 1 |
| 12 | 53762887 | rs7962345 | 12 | 53779775 | rs10876449 | 0.997024 |
| 12 | 53762887 | rs7962345 | 12 | 53782628 | rs11170525 | 1 |
| 12 | 53762887 | rs7962345 | 12 | 53782959 | rs12368491 | 1 |
| 12 | 53770941 | rs10876447 | 12 | 53772927 | rs7131938 | 1 |
| 12 | 53770941 | rs10876447 | 12 | 53774779 | rs36065378 | 1 |
| 12 | 53770941 | rs10876447 | 12 | 53777171 | rs3741651 | 1 |
| 12 | 53770941 | rs10876447 | 12 | 53779775 | rs10876449 | 0.997146 |
| 12 | 53770941 | rs10876447 | 12 | 53782628 | rs11170525 | 1 |
| 12 | 53770941 | rs10876447 | 12 | 53782959 | rs12368491 | 1 |
| 12 | 53770941 | rs10876447 | 12 | 53784913 | rs7315782 | 0.957202 |
| 12 | 53772927 | rs7131938 | 12 | 53774779 | rs36065378 | 1 |
| 12 | 53772927 | rs7131938 | 12 | 53777171 | rs3741651 | 1 |
| 12 | 53772927 | rs7131938 | 12 | 53779775 | rs10876449 | 0.998571 |
| 12 | 53772927 | rs7131938 | 12 | 53782628 | rs11170525 | 1 |
| 12 | 53772927 | rs7131938 | 12 | 53782959 | rs12368491 | 1 |
| 12 | 53772927 | rs7131938 | 12 | 53784913 | rs7315782 | 0.957202 |
| 12 | 53772927 | rs7131938 | 12 | 53785028 | rs2694847 | 1 |
| 12 | 53774779 | rs36065378 | 12 | 53777171 | rs3741651 | 1 |
| 12 | 53774779 | rs36065378 | 12 | 53779775 | rs10876449 | 0.998488 |
| 12 | 53774779 | rs36065378 | 12 | 53782628 | rs11170525 | 1 |
| 12 | 53774779 | rs36065378 | 12 | 53782959 | rs12368491 | 1 |
| 12 | 53774779 | rs36065378 | 12 | 53784913 | rs7315782 | 0.955249 |
| 12 | 53774779 | rs36065378 | 12 | 53785028 | rs2694847 | 1 |
| 12 | 53774779 | rs36065378 | 12 | 53786033 | rs7134665 | 1 |
| 12 | 53777171 | rs3741651 | 12 | 53779775 | rs10876449 | 0.998571 |
| 12 | 53777171 | rs3741651 | 12 | 53782628 | rs11170525 | 1 |
| 12 | 53777171 | rs3741651 | 12 | 53782959 | rs12368491 | 1 |
| 12 | 53777171 | rs3741651 | 12 | 53784913 | rs7315782 | 0.957202 |
| 12 | 53777171 | rs3741651 | 12 | 53785028 | rs2694847 | 1 |
| 12 | 53777171 | rs3741651 | 12 | 53786033 | rs7134665 | 1 |
| 12 | 53777171 | rs3741651 | 12 | 53787866 | 12-53787866 | 0.99706 |
| 12 | 53779775 | rs10876449 | 12 | 53782628 | rs11170525 | 0.998571 |
| 12 | 53779775 | rs10876449 | 12 | 53782959 | rs12368491 | 0.998571 |
| 12 | 53779775 | rs10876449 | 12 | 53784913 | rs7315782 | 0.9557 |
| 12 | 53779775 | rs10876449 | 12 | 53785028 | rs2694847 | 0.998558 |
| 12 | 53779775 | rs10876449 | 12 | 53786033 | rs7134665 | 0.99856 |
| 12 | 53779775 | rs10876449 | 12 | 53787866 | 12-53787866 | 0.998529 |
| 12 | 53779775 | rs10876449 | 12 | 53787953 | rs2460882 | 0.99856 |
| 12 | 53779775 | rs10876449 | 12 | 53788286 | 12-53788286 | 0.830508 |
| 12 | 53782628 | rs11170525 | 12 | 53782959 | rs12368491 | 1 |
| 12 | 53782628 | rs11170525 | 12 | 53784913 | rs7315782 | 0.957202 |
| 12 | 53782628 | rs11170525 | 12 | 53785028 | rs2694847 | 1 |
| 12 | 53782628 | rs11170525 | 12 | 53786033 | rs7134665 | 1 |
| 12 | 53782628 | rs11170525 | 12 | 53787866 | 12-53787866 | 0.99706 |
| 12 | 53782628 | rs11170525 | 12 | 53787953 | rs2460882 | 1 |
| 12 | 53782628 | rs11170525 | 12 | 53788286 | 12-53788286 | 0.832147 |
| 12 | 53782628 | rs11170525 | 12 | 53788335 | rs11170532 | 1 |
| 12 | 53782959 | rs12368491 | 12 | 53784913 | rs7315782 | 0.957202 |
| 12 | 53782959 | rs12368491 | 12 | 53785028 | rs2694847 | 1 |
| 12 | 53782959 | rs12368491 | 12 | 53786033 | rs7134665 | 1 |
| 12 | 53782959 | rs12368491 | 12 | 53787866 | 12-53787866 | 0.99706 |
| 12 | 53782959 | rs12368491 | 12 | 53787953 | rs2460882 | 1 |
| 12 | 53782959 | rs12368491 | 12 | 53788286 | 12-53788286 | 0.832147 |
| 12 | 53782959 | rs12368491 | 12 | 53788335 | rs11170532 | 1 |
| 12 | 53782959 | rs12368491 | 12 | 53789763 | 12-53789763 | 0.832061 |
| 12 | 53784913 | rs7315782 | 12 | 53785028 | rs2694847 | 0.95681 |
| 12 | 53784913 | rs7315782 | 12 | 53786033 | rs7134665 | 0.956866 |
| 12 | 53784913 | rs7315782 | 12 | 53787866 | 12-53787866 | 0.953006 |
| 12 | 53784913 | rs7315782 | 12 | 53787953 | rs2460882 | 0.956866 |
| 12 | 53784913 | rs7315782 | 12 | 53788286 | 12-53788286 | 0.78719 |
| 12 | 53784913 | rs7315782 | 12 | 53788335 | rs11170532 | 0.95719 |
| 12 | 53784913 | rs7315782 | 12 | 53789763 | 12-53789763 | 0.787083 |
| 12 | 53784913 | rs7315782 | 12 | 53790296 | rs2608302 | 0.957202 |
| 12 | 53784913 | rs7315782 | 12 | 53790450 | rs7300593 | 0.957202 |
| 12 | 53785028 | rs2694847 | 12 | 53786033 | rs7134665 | 1 |
| 12 | 53785028 | rs2694847 | 12 | 53787866 | 12-53787866 | 0.997043 |
| 12 | 53785028 | rs2694847 | 12 | 53787953 | rs2460882 | 1 |
| 12 | 53785028 | rs2694847 | 12 | 53788286 | 12-53788286 | 0.831982 |
| 12 | 53785028 | rs2694847 | 12 | 53788335 | rs11170532 | 1 |
| 12 | 53785028 | rs2694847 | 12 | 53789763 | 12-53789763 | 0.831897 |
| 12 | 53785028 | rs2694847 | 12 | 53790296 | rs2608302 | 1 |
| 12 | 53785028 | rs2694847 | 12 | 53790450 | rs7300593 | 1 |
| 12 | 53785028 | rs2694847 | 12 | 53790985 | rs7133236 | 1 |
| 12 | 53786033 | rs7134665 | 12 | 53787866 | 12-53787866 | 0.99706 |
| 12 | 53786033 | rs7134665 | 12 | 53787953 | rs2460882 | 1 |
| 12 | 53786033 | rs7134665 | 12 | 53788286 | 12-53788286 | 0.832186 |
| 12 | 53786033 | rs7134665 | 12 | 53788335 | rs11170532 | 1 |
| 12 | 53786033 | rs7134665 | 12 | 53789763 | 12-53789763 | 0.8321 |
| 12 | 53786033 | rs7134665 | 12 | 53790296 | rs2608302 | 1 |
| 12 | 53786033 | rs7134665 | 12 | 53790450 | rs7300593 | 1 |
| 12 | 53786033 | rs7134665 | 12 | 53790985 | rs7133236 | 1 |
| 12 | 53786033 | rs7134665 | 12 | 53792914 | 12-53792914 | 1 |
| 12 | 53787866 | 12-53787866 | 12 | 53787953 | rs2460882 | 0.99706 |
| 12 | 53787866 | 12-53787866 | 12 | 53788286 | 12-53788286 | 0.826618 |
| 12 | 53787866 | 12-53787866 | 12 | 53788335 | rs11170532 | 0.997059 |
| 12 | 53787866 | 12-53787866 | 12 | 53789763 | 12-53789763 | 0.826532 |
| 12 | 53787866 | 12-53787866 | 12 | 53790296 | rs2608302 | 0.99706 |
| 12 | 53787866 | 12-53787866 | 12 | 53790450 | rs7300593 | 0.99706 |
| 12 | 53787866 | 12-53787866 | 12 | 53790985 | rs7133236 | 0.99706 |
| 12 | 53787866 | 12-53787866 | 12 | 53792914 | 12-53792914 | 0.99706 |
| 12 | 53787866 | 12-53787866 | 12 | 53793209 | rs35969688 | 0.99706 |
| 12 | 53787953 | rs2460882 | 12 | 53788286 | 12-53788286 | 0.832186 |
| 12 | 53787953 | rs2460882 | 12 | 53788335 | rs11170532 | 1 |
| 12 | 53787953 | rs2460882 | 12 | 53789763 | 12-53789763 | 0.8321 |
| 12 | 53787953 | rs2460882 | 12 | 53790296 | rs2608302 | 1 |
| 12 | 53787953 | rs2460882 | 12 | 53790450 | rs7300593 | 1 |
| 12 | 53787953 | rs2460882 | 12 | 53790985 | rs7133236 | 1 |
| 12 | 53787953 | rs2460882 | 12 | 53792914 | 12-53792914 | 1 |
| 12 | 53787953 | rs2460882 | 12 | 53793209 | rs35969688 | 1 |
| 12 | 53787953 | rs2460882 | 12 | 53793653 | rs2947336 | 1 |
| 12 | 53788286 | 12-53788286 | 12 | 53788335 | rs11170532 | 0.832104 |
| 12 | 53788286 | 12-53788286 | 12 | 53789763 | 12-53789763 | 1 |
| 12 | 53788286 | 12-53788286 | 12 | 53790296 | rs2608302 | 0.832147 |
| 12 | 53788286 | 12-53788286 | 12 | 53790450 | rs7300593 | 0.832147 |
| 12 | 53788286 | 12-53788286 | 12 | 53790985 | rs7133236 | 0.83289 |
| 12 | 53788286 | 12-53788286 | 12 | 53792914 | 12-53792914 | 0.831497 |
| 12 | 53788286 | 12-53788286 | 12 | 53793209 | rs35969688 | 0.831497 |
| 12 | 53788286 | 12-53788286 | 12 | 53793653 | rs2947336 | 0.831497 |
| 12 | 53788286 | 12-53788286 | 12 | 53794288 | 12-53794288 | 1 |
| 12 | 53788335 | rs11170532 | 12 | 53789763 | 12-53789763 | 0.832018 |
| 12 | 53788335 | rs11170532 | 12 | 53790296 | rs2608302 | 1 |
| 12 | 53788335 | rs11170532 | 12 | 53790450 | rs7300593 | 1 |
| 12 | 53788335 | rs11170532 | 12 | 53790985 | rs7133236 | 1 |
| 12 | 53788335 | rs11170532 | 12 | 53792914 | 12-53792914 | 1 |
| 12 | 53788335 | rs11170532 | 12 | 53793209 | rs35969688 | 1 |
| 12 | 53788335 | rs11170532 | 12 | 53793653 | rs2947336 | 1 |
| 12 | 53788335 | rs11170532 | 12 | 53794288 | 12-53794288 | 0.832104 |
| 12 | 53788335 | rs11170532 | 12 | 53794787 | rs57676448 | 1 |
| 12 | 53789763 | 12-53789763 | 12 | 53790296 | rs2608302 | 0.832061 |
| 12 | 53789763 | 12-53789763 | 12 | 53790450 | rs7300593 | 0.832061 |
| 12 | 53789763 | 12-53789763 | 12 | 53790985 | rs7133236 | 0.832805 |
| 12 | 53789763 | 12-53789763 | 12 | 53792914 | 12-53792914 | 0.831411 |
| 12 | 53789763 | 12-53789763 | 12 | 53793209 | rs35969688 | 0.831411 |
| 12 | 53789763 | 12-53789763 | 12 | 53793653 | rs2947336 | 0.831411 |
| 12 | 53789763 | 12-53789763 | 12 | 53794288 | 12-53794288 | 1 |
| 12 | 53789763 | 12-53789763 | 12 | 53794787 | rs57676448 | 0.831411 |
| 12 | 53790296 | rs2608302 | 12 | 53790450 | rs7300593 | 1 |
| 12 | 53790296 | rs2608302 | 12 | 53790985 | rs7133236 | 1 |
| 12 | 53790296 | rs2608302 | 12 | 53792914 | 12-53792914 | 1 |
| 12 | 53790296 | rs2608302 | 12 | 53793209 | rs35969688 | 1 |
| 12 | 53790296 | rs2608302 | 12 | 53793653 | rs2947336 | 1 |
| 12 | 53790296 | rs2608302 | 12 | 53794288 | 12-53794288 | 0.832147 |
| 12 | 53790296 | rs2608302 | 12 | 53794787 | rs57676448 | 1 |
| 12 | 53790296 | rs2608302 | 12 | 53796998 | rs2947337 | 1 |
| 12 | 53790450 | rs7300593 | 12 | 53790985 | rs7133236 | 1 |
| 12 | 53790450 | rs7300593 | 12 | 53792914 | 12-53792914 | 1 |
| 12 | 53790450 | rs7300593 | 12 | 53793209 | rs35969688 | 1 |
| 12 | 53790450 | rs7300593 | 12 | 53793653 | rs2947336 | 1 |
| 12 | 53790450 | rs7300593 | 12 | 53794288 | 12-53794288 | 0.832147 |
| 12 | 53790450 | rs7300593 | 12 | 53794787 | rs57676448 | 1 |
| 12 | 53790450 | rs7300593 | 12 | 53796998 | rs2947337 | 1 |
| 12 | 53790450 | rs7300593 | 12 | 53798768 | 12-53798768 | 0.832147 |
| 12 | 53790985 | rs7133236 | 12 | 53792914 | 12-53792914 | 1 |
| 12 | 53790985 | rs7133236 | 12 | 53793209 | rs35969688 | 1 |
| 12 | 53790985 | rs7133236 | 12 | 53793653 | rs2947336 | 1 |
| 12 | 53790985 | rs7133236 | 12 | 53794288 | 12-53794288 | 0.83289 |
| 12 | 53790985 | rs7133236 | 12 | 53794787 | rs57676448 | 1 |
| 12 | 53790985 | rs7133236 | 12 | 53796998 | rs2947337 | 1 |
| 12 | 53790985 | rs7133236 | 12 | 53798768 | 12-53798768 | 0.83289 |
| 12 | 53790985 | rs7133236 | 12 | 53799895 | rs784882 | 1 |
| 12 | 53792914 | 12-53792914 | 12 | 53793209 | rs35969688 | 1 |
| 12 | 53792914 | 12-53792914 | 12 | 53793653 | rs2947336 | 1 |
| 12 | 53792914 | 12-53792914 | 12 | 53794288 | 12-53794288 | 0.832323 |
| 12 | 53792914 | 12-53792914 | 12 | 53794787 | rs57676448 | 1 |
| 12 | 53792914 | 12-53792914 | 12 | 53796998 | rs2947337 | 1 |
| 12 | 53792914 | 12-53792914 | 12 | 53798768 | 12-53798768 | 0.832323 |
| 12 | 53792914 | 12-53792914 | 12 | 53799895 | rs784882 | 1 |
| 12 | 53792914 | 12-53792914 | 12 | 53801461 | 12-53801461 | 1 |
| 12 | 53793209 | rs35969688 | 12 | 53793653 | rs2947336 | 1 |
| 12 | 53793209 | rs35969688 | 12 | 53794288 | 12-53794288 | 0.832323 |
| 12 | 53793209 | rs35969688 | 12 | 53794787 | rs57676448 | 1 |
| 12 | 53793209 | rs35969688 | 12 | 53796998 | rs2947337 | 1 |
| 12 | 53793209 | rs35969688 | 12 | 53798768 | 12-53798768 | 0.832323 |
| 12 | 53793209 | rs35969688 | 12 | 53799895 | rs784882 | 1 |
| 12 | 53793209 | rs35969688 | 12 | 53801461 | 12-53801461 | 1 |
| 12 | 53793209 | rs35969688 | 12 | 53803633 | rs7968637 | 1 |
| 12 | 53793653 | rs2947336 | 12 | 53794288 | 12-53794288 | 0.832323 |
| 12 | 53793653 | rs2947336 | 12 | 53794787 | rs57676448 | 1 |
| 12 | 53793653 | rs2947336 | 12 | 53796998 | rs2947337 | 1 |
| 12 | 53793653 | rs2947336 | 12 | 53798768 | 12-53798768 | 0.832323 |
| 12 | 53793653 | rs2947336 | 12 | 53799895 | rs784882 | 1 |
| 12 | 53793653 | rs2947336 | 12 | 53801461 | 12-53801461 | 1 |
| 12 | 53793653 | rs2947336 | 12 | 53803633 | rs7968637 | 1 |
| 12 | 53793653 | rs2947336 | 12 | 53804307 | rs12817984 | 0.957842 |
| 12 | 53794288 | 12-53794288 | 12 | 53794787 | rs57676448 | 0.832323 |
| 12 | 53794288 | 12-53794288 | 12 | 53796998 | rs2947337 | 0.832323 |
| 12 | 53794288 | 12-53794288 | 12 | 53798768 | 12-53798768 | 1 |
| 12 | 53794288 | 12-53794288 | 12 | 53799895 | rs784882 | 0.839577 |
| 12 | 53794288 | 12-53794288 | 12 | 53801461 | 12-53801461 | 0.831999 |
| 12 | 53794288 | 12-53794288 | 12 | 53803633 | rs7968637 | 0.832323 |
| 12 | 53794288 | 12-53794288 | 12 | 53804307 | rs12817984 | 0.789726 |
| 12 | 53794787 | rs57676448 | 12 | 53796998 | rs2947337 | 1 |
| 12 | 53794787 | rs57676448 | 12 | 53798768 | 12-53798768 | 0.832323 |
| 12 | 53794787 | rs57676448 | 12 | 53799895 | rs784882 | 1 |
| 12 | 53794787 | rs57676448 | 12 | 53801461 | 12-53801461 | 1 |
| 12 | 53794787 | rs57676448 | 12 | 53803633 | rs7968637 | 1 |
| 12 | 53794787 | rs57676448 | 12 | 53804307 | rs12817984 | 0.957842 |
| 12 | 53794787 | rs57676448 | 12 | 53811034 | rs10876450 | 0.984512 |
| 12 | 53796998 | rs2947337 | 12 | 53798768 | 12-53798768 | 0.832323 |
| 12 | 53796998 | rs2947337 | 12 | 53799895 | rs784882 | 1 |
| 12 | 53796998 | rs2947337 | 12 | 53801461 | 12-53801461 | 1 |
| 12 | 53796998 | rs2947337 | 12 | 53803633 | rs7968637 | 1 |
| 12 | 53796998 | rs2947337 | 12 | 53804307 | rs12817984 | 0.957842 |
| 12 | 53796998 | rs2947337 | 12 | 53811034 | rs10876450 | 0.984512 |
| 12 | 53796998 | rs2947337 | 12 | 53811667 | 12-53811667 | 0.816256 |
| 12 | 53796998 | rs2947337 | 12 | 53813402 | rs17098950 | 0.75399 |
| 12 | 53798768 | 12-53798768 | 12 | 53799895 | rs784882 | 0.839577 |
| 12 | 53798768 | 12-53798768 | 12 | 53801461 | 12-53801461 | 0.831999 |
| 12 | 53798768 | 12-53798768 | 12 | 53803633 | rs7968637 | 0.832323 |
| 12 | 53798768 | 12-53798768 | 12 | 53804307 | rs12817984 | 0.789726 |
| 12 | 53798768 | 12-53798768 | 12 | 53811034 | rs10876450 | 0.81684 |
| 12 | 53798768 | 12-53798768 | 12 | 53811667 | 12-53811667 | 0.982696 |
| 12 | 53798768 | 12-53798768 | 12 | 53813402 | rs17098950 | 0.914856 |
| 12 | 53798768 | 12-53798768 | 12 | 53813424 | rs10876451 | 0.816415 |
| 12 | 53799895 | rs784882 | 12 | 53801461 | 12-53801461 | 1 |
| 12 | 53799895 | rs784882 | 12 | 53803633 | rs7968637 | 1 |
| 12 | 53799895 | rs784882 | 12 | 53804307 | rs12817984 | 0.957446 |
| 12 | 53799895 | rs784882 | 12 | 53811034 | rs10876450 | 0.984376 |
| 12 | 53799895 | rs784882 | 12 | 53811667 | 12-53811667 | 0.823258 |
| 12 | 53799895 | rs784882 | 12 | 53813402 | rs17098950 | 0.760311 |
| 12 | 53799895 | rs784882 | 12 | 53813424 | rs10876451 | 0.984376 |
| 12 | 53799895 | rs784882 | 12 | 53813812 | 12-53813812 | 0.984376 |
| 12 | 53801461 | 12-53801461 | 12 | 53803633 | rs7968637 | 1 |
| 12 | 53801461 | 12-53801461 | 12 | 53804307 | rs12817984 | 0.95708 |
| 12 | 53801461 | 12-53801461 | 12 | 53811034 | rs10876450 | 0.984236 |
| 12 | 53801461 | 12-53801461 | 12 | 53811667 | 12-53811667 | 0.815635 |
| 12 | 53801461 | 12-53801461 | 12 | 53813402 | rs17098950 | 0.752974 |
| 12 | 53801461 | 12-53801461 | 12 | 53813424 | rs10876451 | 0.982808 |
| 12 | 53801461 | 12-53801461 | 12 | 53813812 | 12-53813812 | 0.984236 |
| 12 | 53801461 | 12-53801461 | 12 | 53814380 | rs11170549 | 0.984236 |
| 12 | 53803633 | rs7968637 | 12 | 53804307 | rs12817984 | 0.957842 |
| 12 | 53803633 | rs7968637 | 12 | 53811034 | rs10876450 | 0.984512 |
| 12 | 53803633 | rs7968637 | 12 | 53811667 | 12-53811667 | 0.816256 |
| 12 | 53803633 | rs7968637 | 12 | 53813402 | rs17098950 | 0.75399 |
| 12 | 53803633 | rs7968637 | 12 | 53813424 | rs10876451 | 0.983108 |
| 12 | 53803633 | rs7968637 | 12 | 53813812 | 12-53813812 | 0.984512 |
| 12 | 53803633 | rs7968637 | 12 | 53814380 | rs11170549 | 0.984512 |
| 12 | 53803633 | rs7968637 | 12 | 53814714 | rs11170550 | 0.984501 |
| 12 | 53804307 | rs12817984 | 12 | 53811034 | rs10876450 | 0.94385 |
| 12 | 53804307 | rs12817984 | 12 | 53811667 | 12-53811667 | 0.774788 |
| 12 | 53804307 | rs12817984 | 12 | 53813402 | rs17098950 | 0.716146 |
| 12 | 53804307 | rs12817984 | 12 | 53813424 | rs10876451 | 0.942222 |
| 12 | 53804307 | rs12817984 | 12 | 53813812 | 12-53813812 | 0.94385 |
| 12 | 53804307 | rs12817984 | 12 | 53814380 | rs11170549 | 0.94385 |
| 12 | 53804307 | rs12817984 | 12 | 53814714 | rs11170550 | 0.943834 |
| 12 | 53804307 | rs12817984 | 12 | 53814853 | rs2683524 | 0.943162 |
| 12 | 53811034 | rs10876450 | 12 | 53811667 | 12-53811667 | 0.831176 |
| 12 | 53811034 | rs10876450 | 12 | 53813402 | rs17098950 | 0.768822 |
| 12 | 53811034 | rs10876450 | 12 | 53813424 | rs10876451 | 0.998587 |
| 12 | 53811034 | rs10876450 | 12 | 53813812 | 12-53813812 | 1 |
| 12 | 53811034 | rs10876450 | 12 | 53814380 | rs11170549 | 1 |
| 12 | 53811034 | rs10876450 | 12 | 53814714 | rs11170550 | 1 |
| 12 | 53811034 | rs10876450 | 12 | 53814853 | rs2683524 | 1 |
| 12 | 53811034 | rs10876450 | 12 | 53815974 | rs7970368 | 1 |
| 12 | 53811034 | rs10876450 | 12 | 53817237 | rs2002555 | 1 |
| 12 | 53811667 | 12-53811667 | 12 | 53813402 | rs17098950 | 0.932242 |
| 12 | 53811667 | 12-53811667 | 12 | 53813424 | rs10876451 | 0.830642 |
| 12 | 53811667 | 12-53811667 | 12 | 53813812 | 12-53813812 | 0.831176 |
| 12 | 53811667 | 12-53811667 | 12 | 53814380 | rs11170549 | 0.831176 |
| 12 | 53811667 | 12-53811667 | 12 | 53814714 | rs11170550 | 0.831066 |
| 12 | 53811667 | 12-53811667 | 12 | 53814853 | rs2683524 | 0.829152 |
| 12 | 53811667 | 12-53811667 | 12 | 53815974 | rs7970368 | 0.830998 |
| 12 | 53811667 | 12-53811667 | 12 | 53817237 | rs2002555 | 0.830998 |
| 12 | 53813402 | rs17098950 | 12 | 53813424 | rs10876451 | 0.768496 |

# Supplementary Table

**Supplementary Table 5.**

In this study, we calculated the pairwise linkage disequilibrium expressed as the square correlation coefficient (*r^2^*) among SNPs in the genetic region associated with cold sensitivity. In the case of *r^2^*> 0.5, it is shown in the table, and the results displayed in bold are rs11170510 and rs58123204 selected and tested within the region
